# Comparative evaluation of soil DNA extraction kits for long read metagenomic sequencing

**DOI:** 10.1099/acmi.0.000868.v3

**Published:** 2024-09-27

**Authors:** Harry T. Child, Lucy Wierzbicki, Gabrielle R. Joslin, Richard K. Tennant

**Affiliations:** 1 Geography, Faculty of Environment, Science and Economy, University of Exeter, Amory Building, Rennes Drive, Exeter, Devon, EX4 4RJ, UK

**Keywords:** DNA extraction, long-read sequencing, metagenomics, microbiome, Oxford nanopore, soil

## Abstract

Metagenomics has been transformative in our understanding of the diversity and function of soil microbial communities. Applying long read sequencing to whole genome shotgun metagenomics has the potential to revolutionise soil microbial ecology through improved taxonomic classification, functional characterisation and metagenome assembly. However, optimisation of robust methods for long read metagenomics of environmental samples remains undeveloped. In this study, Oxford Nanopore sequencing using samples from five commercially available soil DNA extraction kits was compared across four soil types, in order to optimise read length and reproducibility for comparative long read soil metagenomics. Average extracted DNA lengths varied considerably between kits, but longer DNA fragments did not translate consistently into read lengths. Highly variable decreases in the length of resulting reads from some kits were associated with poor classification rate and low reproducibility in microbial communities identified between technical repeats. Replicate samples from other kits showed more consistent conversion of extracted DNA fragment size into read length and resulted in more congruous microbial community representation. Furthermore, extraction kits showed significant differences in the community representation and structure they identified across all soil types. Overall, the QIAGEN DNeasy PowerSoil Pro Kit displayed the best suitability for reproducible long-read WGS metagenomic sequencing, although further optimisation of DNA purification and library preparation may enable translation of higher molecular weight DNA from other kits into longer read lengths. These findings provide a novel insight into the importance of optimising DNA extraction for achieving replicable results from long read metagenomic sequencing of environmental samples.

**Impact Statement**

Metagenomic sequencing enables an insight into the diversity and function of soil microbial communities, revolutionising our understanding of their contribution to ecosystem function including plant health, nutrient cycling and carbon sequestration. Long read sequencing can improve metagenomic analysis by improving our ability to identify microbes in the soil and characterise the function of genes encoded by their genomes. DNA extraction methods have yet to be optimised for long read metagenomics of environmental samples. The length and purity of DNA extracted using different protocols can vary, potentially impacting their utility for long read sequencing. In this study, five commercial soil DNA extraction kits were compared for long read sequencing of four soil types. Significant differences were identified between the kits in terms of the extracted DNA length, read length and detected microbial communities, as well as the reproducibility of these factors between replicates. These findings highlight the importance of optimisation and standardisation of DNA extraction procedures for long read metagenomics, particularly for difficult sample types like soil.

## Data Summary

Sequence reads have been deposited in the NCBI Sequence Read Archive under BioProject accession PRJNA1090675. Individual sample accession numbers are available in the supplementary information.

## Introduction

Metagenomics provides a powerful tool for studying soil ecosystems, facilitating characterisation of the structure and function of the most diverse microbial communities on the planet [1]. This allows us to investigate how soil microorganisms respond to ecosystem drivers, such as land management and climate change [2,3], and contributions to plant health, nutrient cycling and carbon sequestration [4]. The study of soil metagenomes has also revealed the high proportion of microbial species in soils that are unculturable and therefore overlooked by previous isolate-based methods, revolutionising our understanding of the diversity of subterranean habitats and uncovering a previously untapped wealth of genomic resources for pursuits such as improving soil health, antimicrobial discovery and bioremediation [5,8].

Despite its widespread uptake in the study of environmental samples, challenges still remain for metagenomic sequencing of soil samples. These methodological challenges are often associated with the high complexity of the sample matrix and the microbial communities they contain [9], as well as the remote location of sampling sites, considering the impacts of sample storage on observed microbial communities [10]. Variation in sample collection and processing methods often lead to sources of variability between studies, leading to difficulty in comparative interpretation and meta-analysis. Such sources of variability in sample preparation include the conditions of soil sample storage [11] and the method of nucleic acid extraction [12]. Sources of methodological variability also extend into sequencing and bioinformatics techniques employed, including library preparation, which can introduce bias associated with PCR drift and marker gene primer specificity [13,14], and read classification, associated with analysis tools used and the choice and completeness of the reference database [15].

Addressing challenges pertaining to nucleic acid extraction are particularly important for metagenomics, as this represents the crucial step in obtaining an accurate representation of the microbial community of interest in the analysed genomic diversity of the extracted sample. Nucleic acid extraction efficiency varies between different taxa due to variation in cell surface structures, meaning there is no ‘one-size fits all’ approach to cell lysis. Therefore, the composition of extracted DNA will never be completely consistent with actual microbial community, and leads to variation in the detection and measured abundance of taxa between samples extracted with different methods [12], even in the analysis of homogenous mock communities [16,17]. Furthermore, impurities from soil samples, such as humic acids, act as inhibitor of downstream processes including PCR [18], leading to the requirement for additional purification methods specific to soil [19].

Although most environmental metagenomics has previously utilised PCR-based metabarcoding approaches, namely 16S and 18S ribosomal RNA (rRNA) gene sequencing, advances in sequencing technology, bioinformatic tools and microbial genome databases have increased interest in whole genome shotgun (WGS) metagenomics [20]. As well as circumventing the biases associated with marker gene approaches, WGS metagenomics enables functional characterisation of microbial communities [21], taxonomic classification at higher phylogenetic resolution [22] and assembly of high-quality metagenome assembled genomes from unculturable organisms [23]. Furthermore, WGS approaches capture all organisms in the samples, including viruses, fungi and other eukaryotes, which play critical roles in soil ecosystem function [24,25], but are overlooked by studies using solely 16S rRNA sequencing.

‘Third generation’ long read sequencing technologies, pioneered by PacBio and Oxford Nanopore Technologies (ONT), provide further advantages for WGS metagenomics by enabling more contiguous *de novo* genome assembly, enhanced functional and structural genomic analysis and more accurate taxonomic classification [26,30] compared to short read sequencing. Moreover, long read sequencing can provide benefits for rRNA metabarcoding, enabling sequencing of the full 16S rRNA gene and ITS1/2 gene regions to improve the accuracy and phylogenetic resolution of taxonomic classification [13,31]. Alongside these benefits, recent simultaneous advances in the accuracy and cost efficiency of ONT long read sequencing, as well as the portability and low capital cost of the instruments [32], pave the way for the advancement of in-field metagenomics [10].

It is well established that the choice of extraction method impacts the yield, purity and microbial community representation of DNA from environmental samples, including soil [12,33]. However, many previous studies assessing impact of soil DNA extraction method have focused on DNA yield, and those which assessed microbial diversity have used bacterial 16S rRNA gene sequencing, disregarding crucial impacts on fungal community representation. Furthermore, few studies have assessed the impacts of extraction method simultaneously on multiple soils to uncover general and contrasting trends across heterogeneous sample types [33]. Considering the potential benefits of long read metagenomics and the potential for in-field sequencing with ONT platforms, optimisation of replicable DNA extraction for long read sequencing remains an important topic for investigation. Indeed, balancing the trade-offs between high integrity of extracted DNA and effective lysis of heterogeneous cell types, along with effective removal of inhibitive impurities for reproducible WGS sequencing, represents a considerable challenge.

This study characterises the impact of DNA extraction methods on long read WGS metagenomic sequencing of microbial communities from four diverse soil types. Rather than bespoke extraction protocols, we compared five commercially available soil DNA extraction kits, aiming to improve reproducibility between studies and increase throughput. Quality control of extracted DNA identified variation in fragment length and purity between extraction kits. Variation in extracted DNA length did not consistently correlate with length of sequencing reads, suggesting variability in the maintenance of fragment length across the library preparation between the extraction kits. Variation was identified between the microbial community captured in samples from different kits across all soil types, a significant proportion of which could be explained by the differences in presence-absence of microbial families. Overall, these findings emphasise the importance of optimisation and standardisation of DNA extraction method for WGS metagenomics, and highlight that long fragments of extracted DNA do not necessarily ensure consistent read lengths through long read sequencing.

## Methods

### Soil sample collection and analysis

**Fig. 1.**
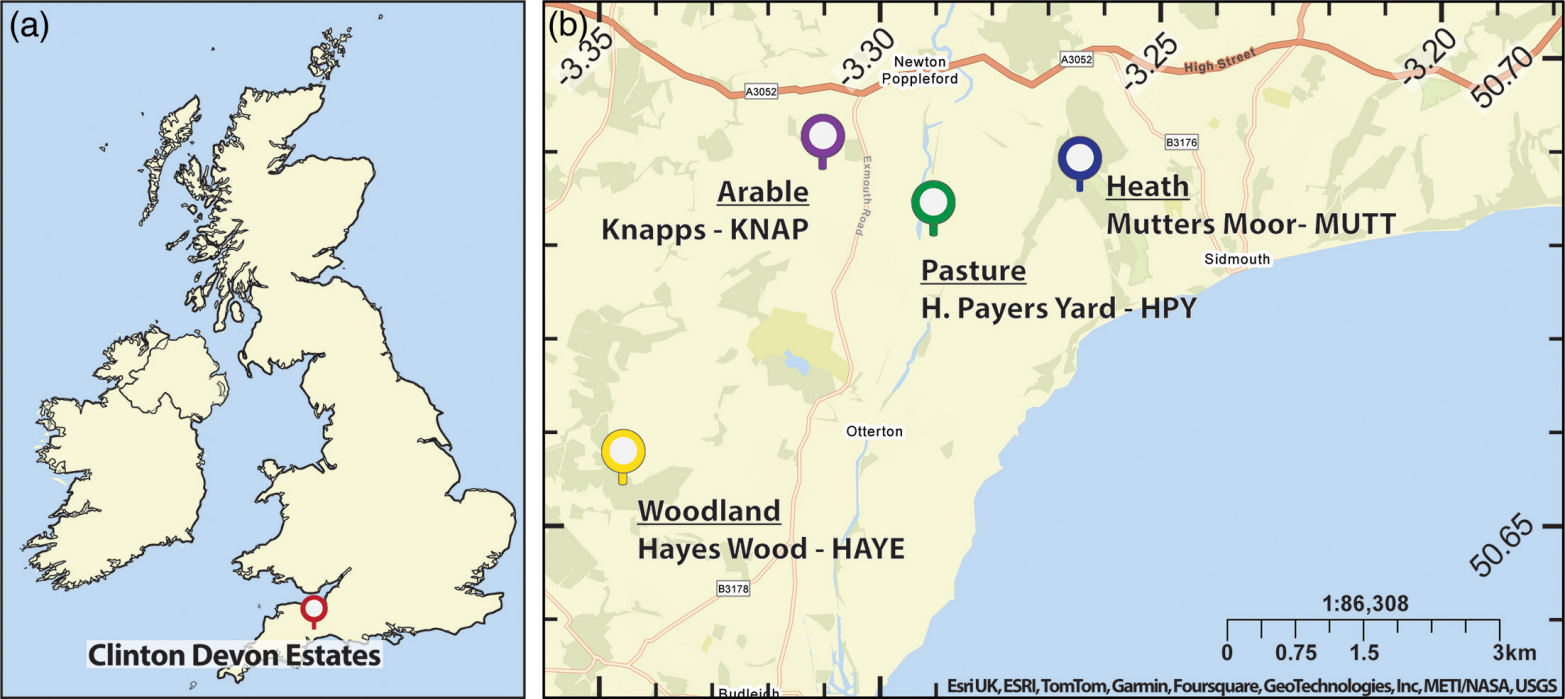
Locations of sampling sites. Map displaying location of Clinton Devon Estates (a) and precise soil sampling locations (b).

Soils were collected from four sites on the Clinton Devon Estates, Devon, UK (Fig. 1; Table 1) between 11 : 00-13 : 00 on 30 June 2023. Sampling sites were chosen to encompass a range of land management strategies and soil types, including dry heath (Mutters Moor; MUTT), ancient deciduous woodland (Hayes Wood; HAYE), improved permanent pasture (H. Pavers Yard; HPY) and an arable field planted at the time with spring Triticale (Knapps Left third; KNAP). Soil was taken from the topsoil (A horizon) of each site to sample the most active microbial community. Soil was passed through a 2 mm sieve and remaining roots were manually removed before being stored at 4 °C for up to 14 days, prior to DNA extraction.

**Table 1.** Soil characteristics at each sampling site

Sample	Site name	Dominant plant species	Sampling depth (cm)	Soil pH	Organic matter content (%)	Carbon content (%)	Field capacity (%)	>250 µm (%)	**63–250 µm (%**)	< 63 µm (%)	Moisture content (%)	NATMAP soil associations
Arable	Knapps Left third (KNAP)	Spring Triticale	5–10	5.91 (SD=0.09)	3.66	2.12	27.72 (SD=0.46)	28.2	25.68	46.12	15.14	Deep loam
Heath	Mutters Moor (MUTT)	Common heather (*Calluna vulgaris*)	5–7	3.87 (SD=0.06)	52.57	30.49	48.34 (SD=2.41)	33.62	21.72	44.66	39.20	Loam over red sandstone
Pasture	Pavers Yard (HPY)	Mixed grass (dominant Ryegrass *Lolium perenne*)	5–12	6.17 (SD=0.06)	5.27	3.04	36.19 (SD=1.43)	12.17	36.63	51.19	21.25	Seasonally wet deep silty
Woodland	Hayes Wood (HAYE)	Oak (*Quercus robur*) and Beech (*Fagus sylvatica*)	3–5	3.5 (SD=0.04)	12.95	7.51	41.80 (SD=1.80)	36.36	38.4	25.24	23.30	Deep loam

Organic matter content was measured by loss on ignition in a furnace, which was converted to carbon content using the van Bemmelen factor:

CarbonContent(%)=OrganicMatterContent(%)x0.58

Soil pH was measured on a 1 : 2.5 (soil:deionised water) suspension of soil, with the mean of triplicate measurements taken. Field capacity was measured by saturating approximately 3 g of soil a Whatman no.4 filter paper in a funnel. Three consecutive water additions were carried out with excess and the soil allowed to fully drain in between. Samples were oven-dried at 105 °C until the mass ceased to change, and the moisture content calculated as:

$$Watercontent=\left(\frac{WetWeight-DryWeight}{DryWeight}\right)\text{x 100\%}$$

Field capacity was taken as the mean water content of three replicates of each soil type. Soil texture was measured by sonication of soil samples at 200 J ml^−1^ to break down aggregates, with subsequent wet sieving to measure particle size composition. Soil characteristics for each site were also accessed from the National Soil Map of England and Wales (NATMAP).

### DNA extraction

DNA extractions were carried out using five soil-specific commercial kits, including FastDNA SPIN Kit for Soil (MP Biomedicals, UK), SPINeasy DNA Pro Kit for Soil (MP Biomedicals, UK), MagBeads FastDNA Kit for Soil (MP Biomedicals, UK), DNeasy PowerSoil Pro Kit (Qiagen, UK) and Zymo Research Quick-DNA Faecal/Soil Microbe MiniPrep^TM^ Kit (Cambridge Bioscience, UK), detailed in Table 2. Ten aliquots of ~250 mg for each soil type were measured into the lysis tubes for each extraction kit and frozen at −20 °C, along with four lysis tubes to be used for negative controls. DNA was extracted by two users for each kit (five replicates of each soil type and two negative controls for each kit), with User A following the aforementioned order of kits and User B following the reverse, with extractions on consecutive days over 5 days. Extractions were carried out as per the manufacturer’s protocols, the utilised versions of which are included in Supplementary Material. Details of lysis conditions and minor modifications are detailed below.

**Table 2.** Details of DNA extraction kits and parameters used

Kit abbreviation	Full product name	Supplier catalogue no.	Supplier	Lysis conditions	Input soil wt (mg)	Elution vol. (µl)	DNA isolation method
FastDNA	FastDNA SPIN Kit for Soil	SKU: 116 560 300	MP Biomedicals, UK	40 s at 6 m s^−1^	250	100 (200 for user B)	Suspended DNA binding matrix
SPINeasy	SPINeasy DNA Pro Kit for Soil	SKU: 116 546 050	MP Biomedicals, UK	40 s at 6 m s^−1^	250	100	Column DNA binding matrix
MagBeads	MagBeads FastDNA Kit for Soil	SKU: 116 561 050	MP Biomedicals, UK	40 s at 6 m s^−1^	250	100	Magnetic beads
PowerSoil	DNeasy PowerSoil Pro Kit	Cat. No. 47 014	Qiagen, UK	40 s at 6 m s^−1^	250	100	Column DNA binding matrix
Zymo	Zymo Research Quick-DNA Faecal/Soil Microbe MiniPrep^TM^ Kit	D6010	Cambridge Bioscience, UK	5 m at 6.5 m s^−1^	250 (100 for MUTT samples)	100	Column DNA binding matrix

Mechanical lysis was carried out using a FastPrep-24 5G (MP Biomedicals, UK) bead beater, with samples lysed for 40 s at 6 m s^−1^ for all extraction kits apart from Zymo, for which five consecutive rounds of 1 min at 6.5 m s^−1^ and 1 min incubation on ice was used, as per the manufacturer’s recommendations. Negative controls were prepared by adding 200 µl nuclease-free water to the lysis tubes. Extraction of the heath soil using the Zymo kit was repeated with 100 mg input due to difficulty recovering the highly viscous supernatant of the lysate when using 250 mg. DNA samples were eluted in 100 µl of the respective elution buffers, apart from FastDNA extractions carried out by User B which were eluted in 200 µl due to difficulty resuspending the binding matrix in a lower volume.

DNA concentration was quantified using the Invitrogen Qubit dsDNA Broad Range Quantification Kit (Fisher Scientific, UK) on an Invitrogen Qubit Flex Fluorometer (Fisher Scientific, UK). Integrity and size of extracted DNA fragments was quantified using the 4200 TapeStation System (Agilent, UK) with the Genomic DNA ScreenTape assay (Agilent, UK). DNA purity was assessed by measuring the 260 : 280 ratio of a 2 µl droplet using an LVis Plate (BMG Labtech, UK) on a FLUOstar Omega (BMG Labtech, UK) microplate reader.

### Library preparation and sequencing

Library preparation was carried out using the Ligation sequencing V14 kit with PCR barcoding expansion (SQK-LSK114 with EXP-PBC096; Oxford Nanopore Technologies [ONT], UK), following the manufacturer's protocol. DNA samples were randomised across three 96-well plates, and samples QIA_KNAP_6 and QIA_HPY_7 were omitted due to a technical error. A total of 200 fmol DNA (or 50 µl of eluted DNA if the concentration was too low) was used in the end-repair reaction. PCR barcoding was carried out in 50 µl reactions using 20 ng DNA (or 15 µl of eluted DNA if the concentration was too low) with LongAmp Taq 2X Master Mix (New England Biolabs, UK). Equimolar pools of barcoded samples were prepared, sequencing adapters were ligated and final libraries were sequenced across two PromethION Flow Cells (R10.4.1) and one MinION Flow Cell (R10.4.1; ONT, UK) using an ONT GridION coupled to an ONT PromethION P2 Solo.

### Bioinformatics

High-accuracy basecalling was performed using Guppy v6.3.9 and demultiplexing carried out with Guppy v6.3.7. Sequencing statistics for each sample were generated using the ‘stats’ function in SeqKit v2.5.0 [34]. Reads were classified using DIAMOND v2.1.7 [35] against the NCBI nr database downloaded on second August 2022, using the parameters ‘--long-reads --block-size 6 --threads 20’, before being processed with daa-meganizer using MEGAN Community Edition v6.24.22 [36], using the parameters ‘--minSupport 10 --longReads --readAssignmentMode readCount --mdb megan-map-Feb2022-ue.db’. Taxonomic classification of reads was carried out using the DIAMOND +MEGAN long read pipeline [37], with read files randomly subsampled to 50  000 reads to enhance analysis efficiency. Samples with fewer than 2000 aligned reads were omitted from downstream analysis, as these tended to have very low complexity which considerably skewed ordination analyses. Count tables of microbial families were generated and filtered to remove those with supporting alignments from fewer than two samples for any combination of soil sample and extraction kit. Rarefaction analysis revealed that subsampling did not limit the family richness encompassed in each sample, with all samples plateauing before 50  000 reads (Fig. S1, available in the online Supplementary Material). Beta diversity matrices were generated using the avgdist function in the vegan (v2.6–4) R package [38], using the parameters ‘avgdist(dmethod = ‘bray’, sample=min_count_sum)’ for Bray-Curtis distances and ‘avgdist(dmethod = ‘jaccard’, sample=min_count_sum)’ for Jaccard distances, where ‘min_sum_count’ is set to the sample with the lowest summed counts. Permutational multivariate analysis of variance (PERMANOVA) was carried out on these distance matrices using the adonis2 function in vegan, while pairwise PERMANOVA was carried out using the pairwise.adonis2 function [39] with the parameters ‘p.method = ‘BH’, nperm=999’. Analysis of multivariate homogeneity of group dispersions was carried out using the betadisper function in vegan. Taxa with significantly different relative abundance between groups were calculated using the ANCOMBC2 version 2.4.0 package [40] with a *P*-value cut-off of 0.01.

## Results

### Characterisation of soil samples

Topsoil (A horizon) samples were taken from four sites (Fig. 1), including two agricultural soils (permanent pasture and arable fields) and two natural habitats (dry heath and deciduous woodland), representing a range of physical and chemical soil characteristics (Table 1). This included acidic soils from the heath and woodland sites and more neutral soils from the agricultural sites (Table 1). The samples also varied dramatically in organic matter content, with high organic matter in the heath sample (52.57%) and lower organic matter in agricultural soils (3.66% in arable and 5.27% in pasture samples; Table 1). Soil texture was finer for the pasture sample and coarser for the woodland sample, assessed using data from the NATMAP database and corroborated with laboratory texture analysis (Table 1). Furthermore, with the range in land management and habitat types and their associated plant species, a diverse range of microbial communities is likely to be present for assessment of DNA extraction efficiency.

### Quantity, quality, and purity of DNA varies between extraction kits

**Fig. 2.**
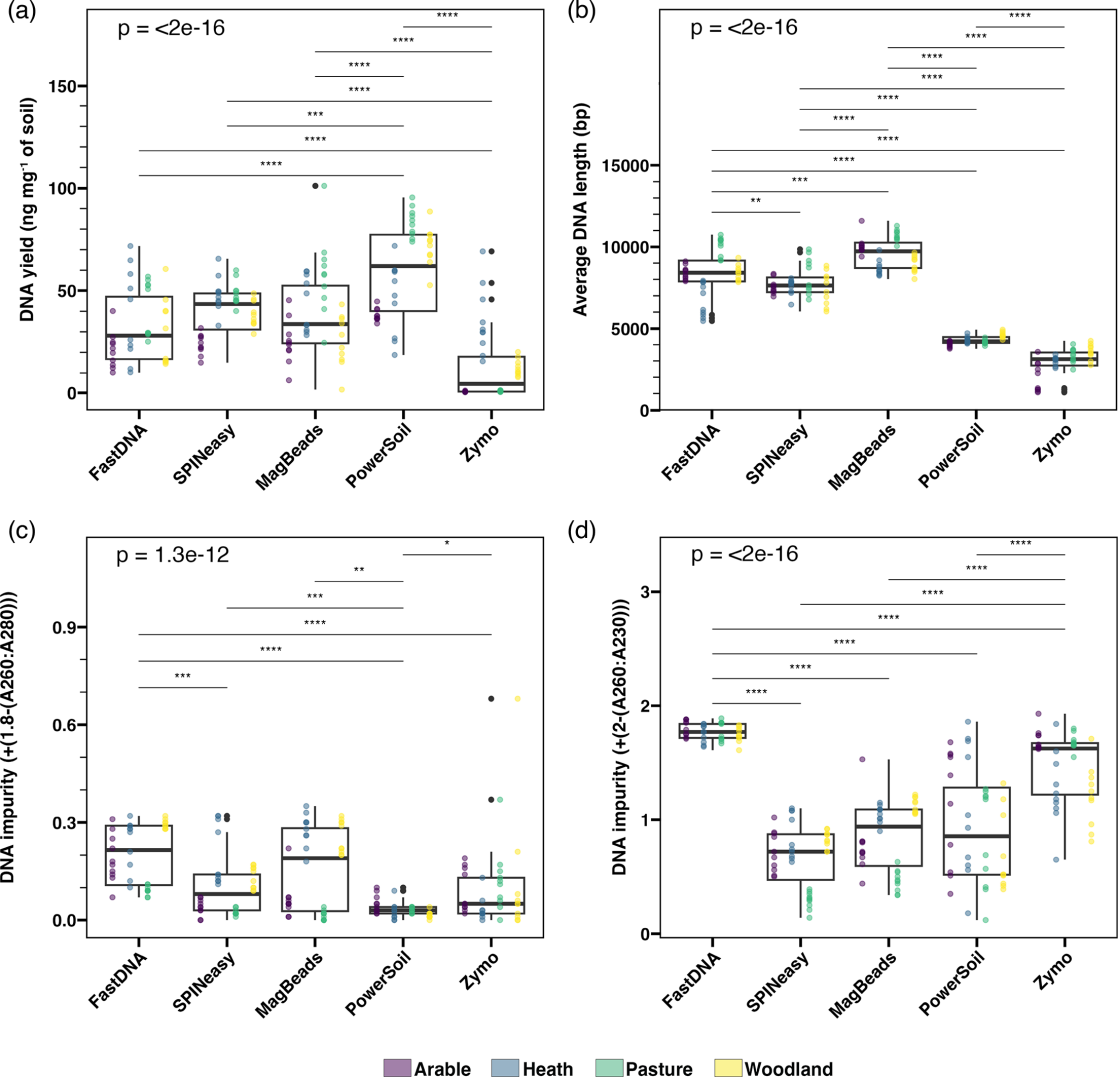
Extracted DNA varies in yield, purity and integrity between extraction kits. Boxplots of (a) DNA yield and (b) average DNA length calculated using TapeStation traces (c) purity, calculated by the absolute difference between the 260 : 280 ratio of the sample from 1.8 and (d) purity, calculated by the absolute difference between the 260 : 230 ratio of the sample from 2.0. Coloured points show data points from arable (purple), heath (blue), pasture (green) and woodland (yellow) soil types. *P*-values are given for the results of a Kruskal-Wallis test across all groups, as well as significant pairwise comparisons using Wilcoxon signed-rank test (*: *P*≤0.05; **: *P*≤0.01; ***: *P*≤0.001; ****: *P*≤0.0001).

DNA was extracted from all soil types using five different extraction kits: FastDNA, SPINeasy, MagBeads, PowerSoil and Zymo (Table 2). No significant difference in the DNA yield per milligram of soil was identified between the FastDNA (32±17 ng mg^−1^), SPINeasy (40±12 ng mg^−1^) and MagBeads (38±20 ng mg^−1^) kits overall (Fig. 2a), or within each soil type (Fig. S3a). The DNA yield was significantly higher from the PowerSoil kit (60±21 ng mg^−1^), and significantly lower from the Zymo kit (12±16 ng mg^−1^) compared to all other kits (Fig. 2a). These patterns were largely consistent across all soil types, apart from the dry heath soil, which displayed no significant difference in yield between extraction kits (Fig. S3a). All negative controls yielded undetectable quantities of DNA.

DNA yield was significantly higher for User B when using the FastDNA kit (Fig. S3a). This is likely caused by the higher elution volume used by User B due to difficulty resuspending the binding matrix, which is manually pipetted into the column after resuspension and can therefore vary in volume between extractions. The MagBeads kit also displayed significant variation in DNA yield between users (Fig. S4a), which may be caused by the presence of more manual resuspension and elution steps in this protocol.

The level of DNA impurity was assessed for each sample by calculating the absolute difference of the measured A260:A280 value from 1.8 and the A260:A280 value from 2, based on the expected values for pure DNA (Fig. 2c, d). Samples extracted using the PowerSoil kit showed significantly higher A260:A280 purity compared to all other kits (Fig. 2c), which was consistent across all soil types (Fig. S3b). Furthermore, samples from the FastDNA kit showed significantly lower A260:A280 purity compared to the SPINeasy and Zymo kits overall (Fig. 2c), and to the MagBeads kit for the arable and pasture soils (Fig. S2b). The MagBeads kit showed mixed performance in terms of A260:A280 purity, yielding high purity from arable and pasture soils but low purity from heath and woodland samples (Fig. S2b). The Zymo kit displayed the opposite of this trend, although the A260:A280 purity of samples was only significantly lower when compared to other kits for the pasture soil (Fig. S3b). The SPINeasy kit yielded DNA with good A260:A280 purity from all samples apart from the heath soil (Fig. S3b). No significant difference in DNA A260:A280 purity was observed between the users for any of the extraction kits (Fig. S4b).

Similarly, the FastDNA kit resulted in significantly lower A260:A230 purity than all other kits, while the Zymo kit also showed poor A260:A230 purity in contrast to the results from A260:A280 analysis (Fig. 2d). With all soil types considered, the SPINeasy, MagBeads and PowerSoil kits displayed no significant difference in A260:A230 purity (Fig. 2d), while the SPINeasy kit showed the highest A260:A230 purity of the three from the pasture soil and significantly higher purity than the MagBeads kit in woodland samples (Fig. S3c). A significant difference in A260:A230 purity was found between users for the FastDNA and PowerSoil kits (Fig. S4c). Generally, extractions showed worse A260:A230 than A260:A280 ratios, which is not surprising considering the absorbance of humic acids at <250 nm [41]. Overall, these findings indicate that the FastDNA yielded the lowest purity DNA, while the PowerSoil and SPINeasy kits showed the highest purity.

Due to the importance of DNA fragment length for long-read sequencing, the quality of DNA extracts was assessed using the DNA Integrity Number (DIN) and average DNA fragment length. The MagBeads kit resulted in the best DNA quality, with significantly higher DIN and average fragment length (9609±951 bp) than all other kits (Figs 2b and S2).

This was closely followed in terms of average fragment length by the FastDNA (8414±1346 bp) and SPINeasy kits (7720±857 bp; Fig. 2b), which also displayed similarly high DINs (Fig. S2). The average fragment length of DNA samples extracted with the PowerSoil Kit (4286±272 bp) was significantly lower than the aforementioned kits (Fig. 2b). The Zymo kit displayed the worst quality DNA extracts in terms of DIN and average fragment length (3004±816 bp; Figs 2 and S2). These results were consistently replicated when separating the results by input soil type (Fig. S3c, d). Significant differences in DIN were identified between users for the SPINeasy and PowerSoil kits, although the effect sizes were minimal in both cases (Fig. S4c). The significant difference in DIN between users for the Zymo kit is likely caused by the extremely low DNA yield for the arable and pasture soils (Fig. S3a), leading to many samples not receiving a DIN value (Fig. S4c). Average DNA length was consistent between users apart from the SPINeasy kit, which resulted in significantly lower fragment size for User B (Fig. S4d).

Overall, these results suggest that while the PowerSoil kit resulted in the highest DNA yield and purity, the quality and fragment length of DNA was higher for extracts from the MagBeads, FastDNA and SPINeasy kits. These three kits gave similarly good yields, but varied in the purity of resulting DNA, with the SPINeasy kit resulting in the best purity and the FastDNA kit the worst purity of these kits. The Zymo kit resulted in the lowest quality and quantity of DNA, despite giving extracts with generally high purity.

### The maintenance of DNA fragment length during library preparation is not consistent between kits

**Fig. 3.**
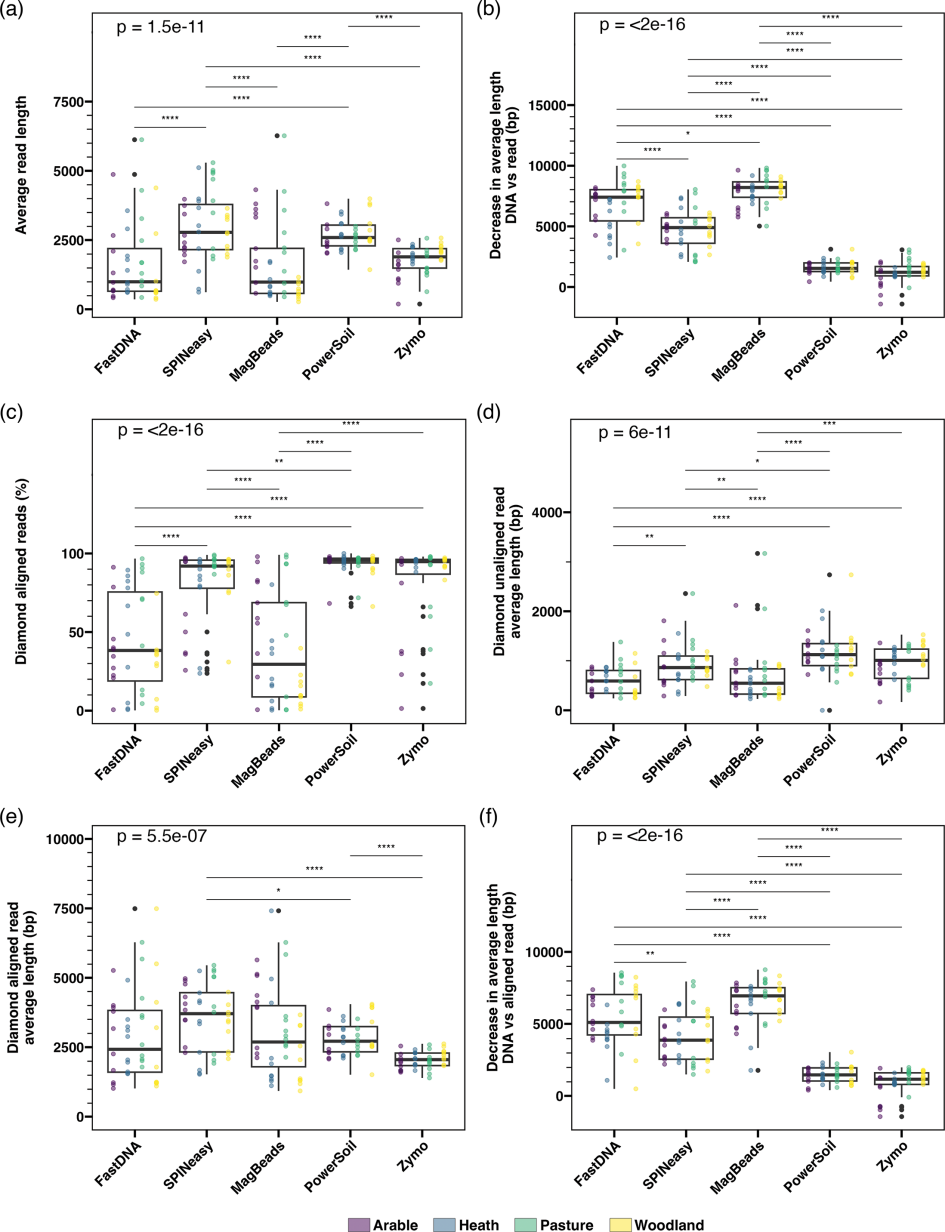
Extraction method impacts read lengths and taxonomic classification rates. Boxplots of (**a**) average read length, (**b**) the decrease in average length between extracted DNA and reads, (**c**) percentage of reads aligned to NCBI nr using DIAMOND, average length of unaligned (**d**) and aligned (**e**) reads, and (**f**) the decrease in average length between extracted DNA and aligned reads. Coloured points show data points from arable (purple), heath (blue), pasture (green) and woodland (yellow) soil types. *P*-values are given for the results of a Kruskal-Wallis test across all groups, as well as significant pairwise comparisons using Wilcoxon signed-rank test (*: *P*≤0.05; **: *P*≤0.01; ***: *P*≤0.001; ****: *P*≤0.0001).

The average length of sequencing reads for each sample was compared between the kits to assess their performance for long-read metagenomic sequencing. Despite having significantly higher DNA fragment length than all other kits after DNA extraction, the FastDNA and MagBeads kits both led to significantly shorter average read lengths than those generated by the SPINeasy and PowerSoil kits (Fig. 3a). This is reflected in the difference in average fragment length between extracted DNA and sequencing reads of each sample, which was greatest for the MagBeads kit, followed by the FastDNA kit (Fig. 3b). The SPINeasy and PowerSoil kits also resulted in significantly longer reads than the Zymo kit (Fig. 3a), which started with significantly shorter extracted DNA than all other kits (Fig. 1d) but did maintain fragment length in sequencing reads relatively well (Fig. 3b). While there was no significant difference in the average read length between the PowerSoil and SPINeasy kits (Fig. 3a), the loss of fragment length was significantly greater for the SPINeasy kit (Fig. 3b), which originally produced significantly longer extracted DNA (Fig. 2d). Read lengths for samples extracted with the PowerSoil kit were also less variable than the SPINeasy kit (Fig. 3a). These patterns were observed consistently across the soil types (Fig. S5a, b).

Reads from samples extracted with the FastDNA and MagBeads kits had significantly lower alignment rates than all other kits when classified using DIAMOND, which was also true for SPINeasy samples when compared to PowerSoil samples (Fig. 3c). Investigation of the unaligned reads found them to encompass a large proportion of the smallest reads in all samples, which was particularly apparent in the samples from the FastDNA and MagBeads kits (Fig. 3d). These short sequences were largely found to consist of PCR artefacts, including repetitive sequences and concatemers of sequencing adapter and barcodes. Furthermore, the efficiency of barcoding PCR reactions was found to be significantly lower for samples from FastDNA and MagBeads kits (Fig. S5c), indicating higher PCR inhibition in these samples.

When these unaligned sequences were disregarded, the average size of aligned sequences was found to be more consistent between the extraction kits, with the only significant differences being the higher average aligned read length in SPINeasy samples compared to PowerSoil and Zymo samples, and the PowerSoil compared to the Zymo samples (Fig. 3e). Despite this, the loss in fragment length in aligned reads compared to extracted DNA was still much higher for the FastDNA, MagBeads and SPINeasy kits (Fig. 3f), indicating that this observation is true regardless of the increased presence of PCR artefacts in these samples.

**Fig. 4.**
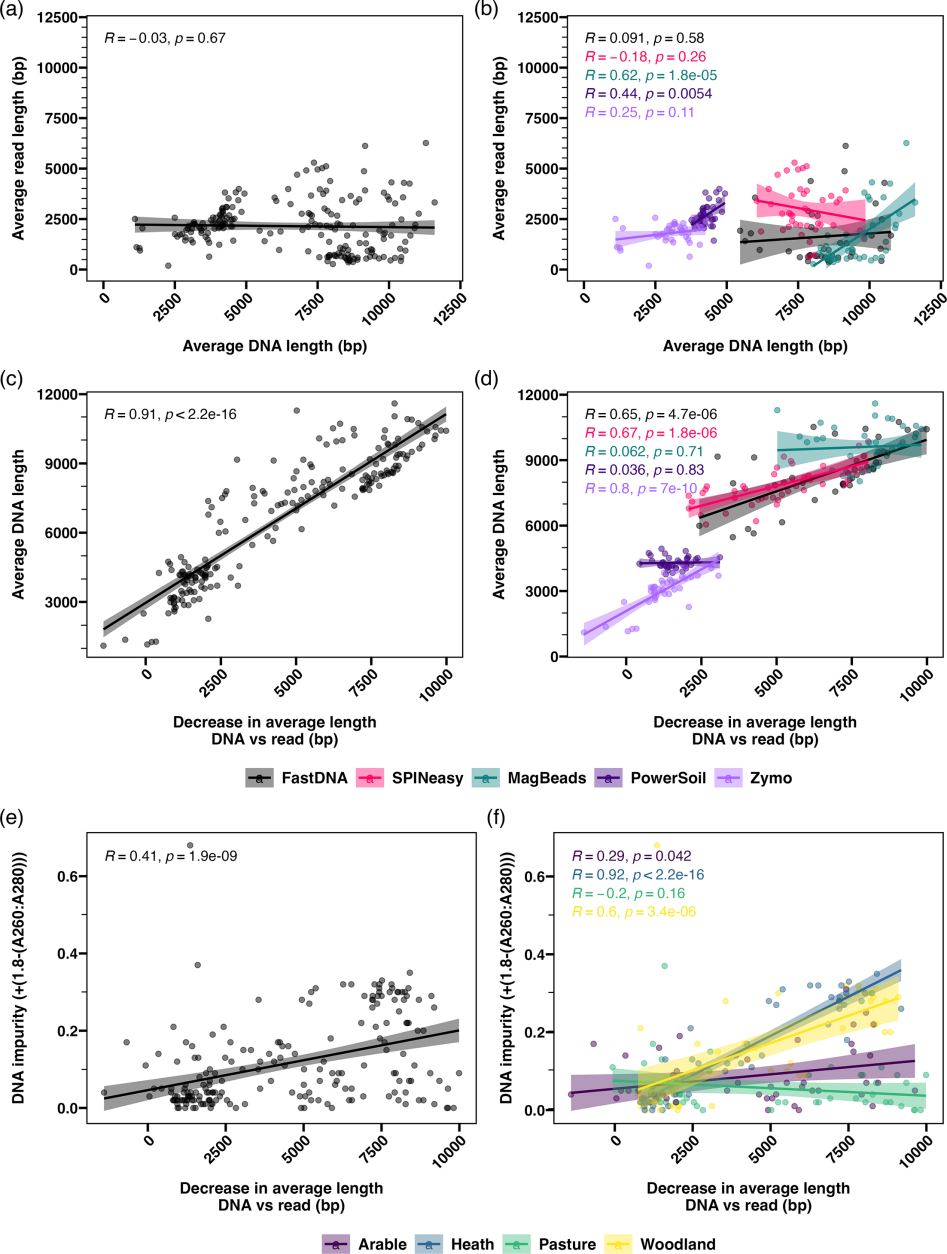
Decrease in DNA length between extracted DNA and reads correlates with fragment length and sample purity. Relationship between (a, b) average read length and extracted DNA length, (c, d) average DNA length and the decrease in average length between extracted DNA and reads, and (e, f) DNA impurity and the decrease in average length between extracted DNA and reads. Pearson correlation coefficients are calculated overall (a, c and e), within extraction kits (b and d) or within soil types (f), with R- and *P*-values provided.

Across all samples, average DNA fragment length did not correlate with average read length, confirming the lack of consistency in fragment length loss between samples and extraction kits (Fig. 4a). When extraction kits were analysed separately, the PowerSoil and MagBeads kits show a significant correlation between extracted DNA and read length (Fig. 4b), indicating that the loss in fragment length across the library preparation was consistent between samples from these methods. For the FastDNA, SPINeasy and Zymo kits, average read length did not correlate with DNA fragment length, indicating that the loss of fragment length varied between samples for these kits (Fig. 4b).

There was a strong positive correlation between the average length of extracted DNA and the length of DNA lost between extraction and sequencing across all samples (Fig. 4c), which was reflected in samples from the FastDNA, SPINeasy and Zymo kits when data was grouped by extraction kit (Fig. 4d). However, this correlation was not present amongst samples extracted with the PowerSoil and MagBeads kits (Fig. 4d), likely due to the low variability in the extracted DNA length for these kits. This indicates that, to some extent, samples with longer extracted DNA fragments showed a larger absolute decrease in DNA length across the library preparation. There was also a significant correlation between the loss of DNA fragment length between extraction and sequencing and DNA A260:A280 impurity (Fig. 4e). However, this correlation was not found when samples were grouped by extraction kit (Fig. S6), indicating that DNA impurity was not impacting loss of DNA fragment length consistently across the kits. Grouping samples by soil type revealed that this correlation was largely driven by heath and woodland soil samples (Fig. 4f), which also displayed the largest differences in DNA impurity between the kits (Fig. S3b). Furthermore, although there was no significant correlation between A260:A230 ratios and loss of DNA fragment length overall (Fig. S6b), there was a significant correlation in woodland samples when analysed separately (Fig. S6c). This suggests that DNA impurity may be influencing the loss of DNA length, but that the metrics of DNA purity applied here did not capture any impact of impurities on fragment size for certain soil types.

### Microbial community richness varies between extraction kits

**Fig. 5.**
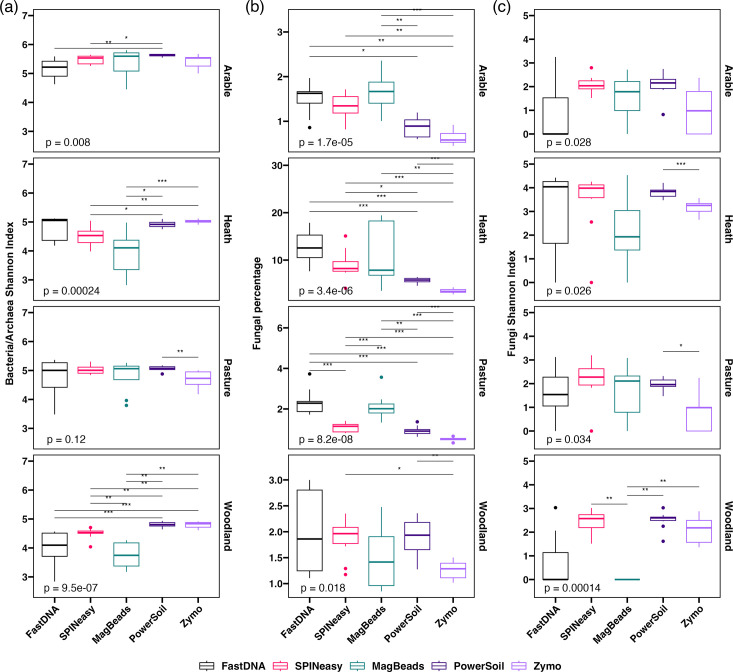
Richness of microbial communities varies across extraction methods. Boxplots of (**a**) Shannon-Weaver index of bacterial and archaeal reads, (**b**) percentage of reads classified as fungi and (**c**) Shannon-Weaver index of fungal reads, separated by soil type. *P*-values are given for the results of a Kruskal-Wallis test across all groups, as well as significant pairwise comparisons using Wilcoxon signed-rank test (*: *P*≤0.05; **: *P*≤0.01; ***: *P*≤0.001; ****: *P*≤0.0001).

Bacterial and archaeal alpha diversity at family level was consistently high in samples from the PowerSoil kit (Fig. 5a). This was particularly apparent in the heath and woodland soils, with significantly higher alpha diversity in PowerSoil samples compared to the MagBeads and SPINeasy kits, as well as the FastDNA kit in the woodland soil (Fig. 5a). Samples extracted with the Zymo kit also had similarly high prokaryotic alpha diversity in these soil types, but were less consistent than the PowerSoil samples in the pasture and arable soils (Fig. 5a). Furthermore, samples from the FastDNA and MagBeads kits showed higher variation in prokaryotic alpha diversity (Fig. 5a), likely linked to the low percentage of aligned reads in many of these samples.

Due to the difficulty in lysing fungal cells during DNA extraction from soil, the percentage of reads assigned to fungi was assessed. The fungal read percentage was much higher in heath soil samples than other soil types, which is expected for this fungal dominated ericaceous ecosystem. Overall, samples extracted with the Zymo kit displayed the lowest fungal percentage across all soil types (Fig. 5b), as well as displaying significantly lower fungal alpha diversity than the PowerSoil kit for heath and pasture soils (Fig. 5c). However, the Zymo kit showed higher fungal diversity than the FastDNA and MagBead kits for woodland soil (Fig. 5c).

Furthermore, despite the fungal read percentage being lower for the PowerSoil kit than other kits (apart from Zymo) across most soil types, the fungal alpha diversity was equivalent or higher, as well as more consistent, in PowerSoil samples than other kits across all soil types (Fig. 5, c). This suggests that increasing fungal read percentage through extraction of higher concentrations of fungal DNA does not necessarily lead to capturing increased fungal diversity.

### Extraction method impacts the observed microbial community composition

**Fig. 6.**
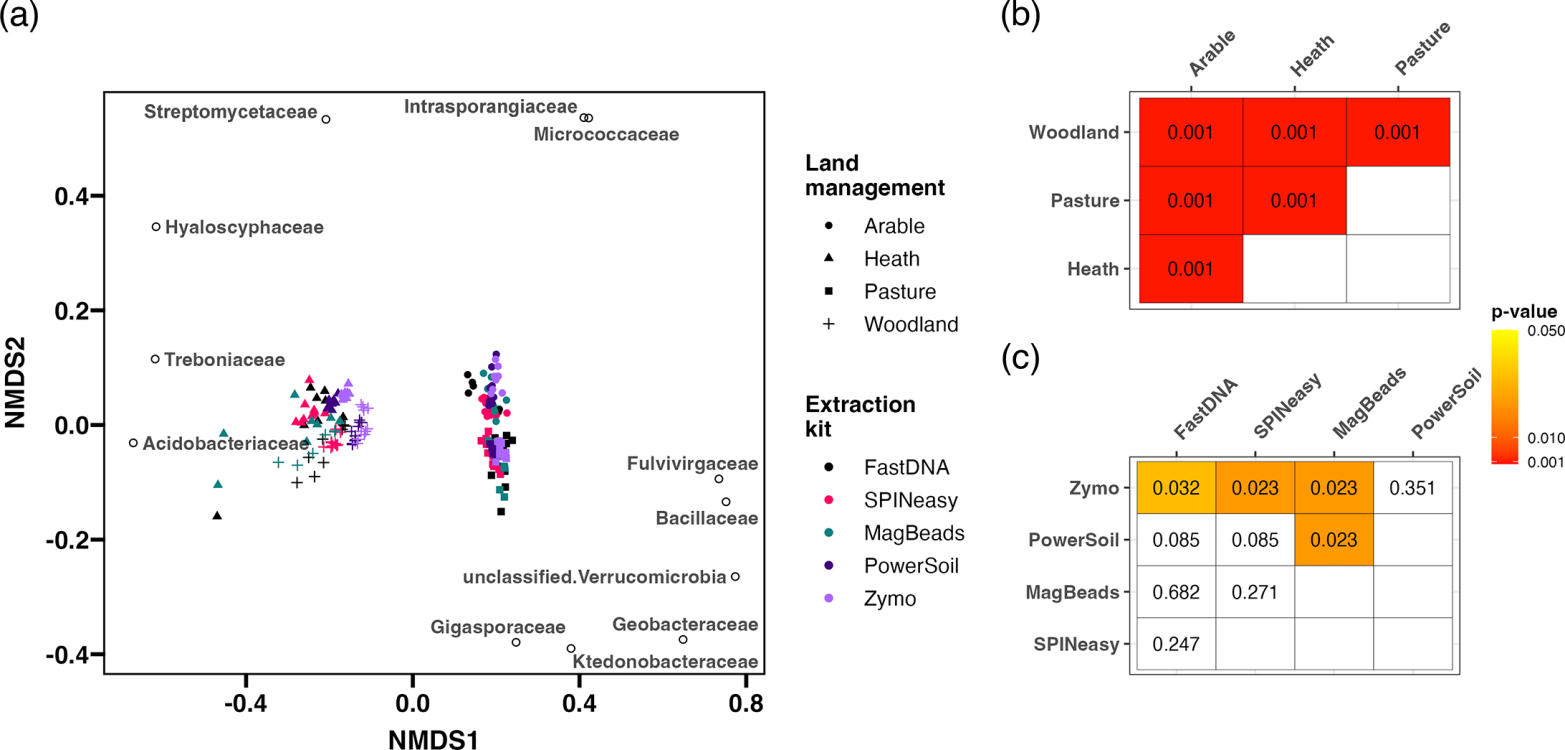
Soil type dominates variability in the measured microbial community. (**a**) Nonmetric Multidimensional Scaling (NMDS) plot based on Bray-Curtis distances between all samples, generated on count data from reads assigned to bacteria, archaea and fungi. The top three microbial families that correlate with the extremes of each NMDS axis are displayed as black circles. The stress value for this analysis was 0.08. Benjamini-Hochberg corrected *P*-values are displayed from pairwise PERMANOVA comparing soil types (**b**) and extraction kits (**c**).

Beta diversity analysis based on Bray-Curtis distances identified significant variation between the microbial communities based on soil type (Fig. 6a; PERMANOVA R^2^=0.63; *P*<0.001), with highly significant pairwise differences between the communities from each land use (Fig. 6b). Samples broadly segregated into clusters from agricultural grass-dominated habitats (arable and pasture) and natural habitats with acidic soils (heath and woodland). This was characterised by the high abundance of Acidobacteriaceae in woodland and heath samples (Fig. 6a), a bacterial family known to be abundant and grow optimally in acidic conditions such as those in these soil types [42]. Furthermore, there was a higher abundance of Verrucomicriobia in agricultural soils, particularly pasture samples (Fig. 6a), consistent with the dominance of this phylum in grassland soils [43]. Beta diversity analysis across all soil types also identified a significant difference between the microbial communities of samples from different extraction kits (Fig. 6a and c; PERMANOVA *R*^2^=0.06; *P*<0.001), although this only explained a small proportion of the variation. Furthermore, pairwise comparisons only identified significant differences between the Zymo kit and all three MPBio kits, as well as the PowerSoil kit and MagBeads kit (Fig. 6c). However, these differences were masked by the dominance of the variation between soil types, prompting analysis of each soil type individually.

The majority of negative controls resulted in minimal classified read counts below the filtering threshold. Three negative controls did result in classified read counts above the filtering threshold, which appeared to consist of community composition similar to heath and woodland samples. These reads likely result from cross contamination during the library preparation process, with minute quantities of contaminant DNA being amplified during PCR barcoding in negative controls containing unquantifiable DNA concentrations. However, due to high concentrations of DNA in all other samples, such cross contamination is unlikely to impact the observed communities. Furthermore, all samples grouped perfectly by soil type in beta diversity analysis, despite randomisation of samples across 96 well plates before library preparation, further supporting the conclusion that cross contamination had minimal impact on the results of the study.

**Fig. 7.**
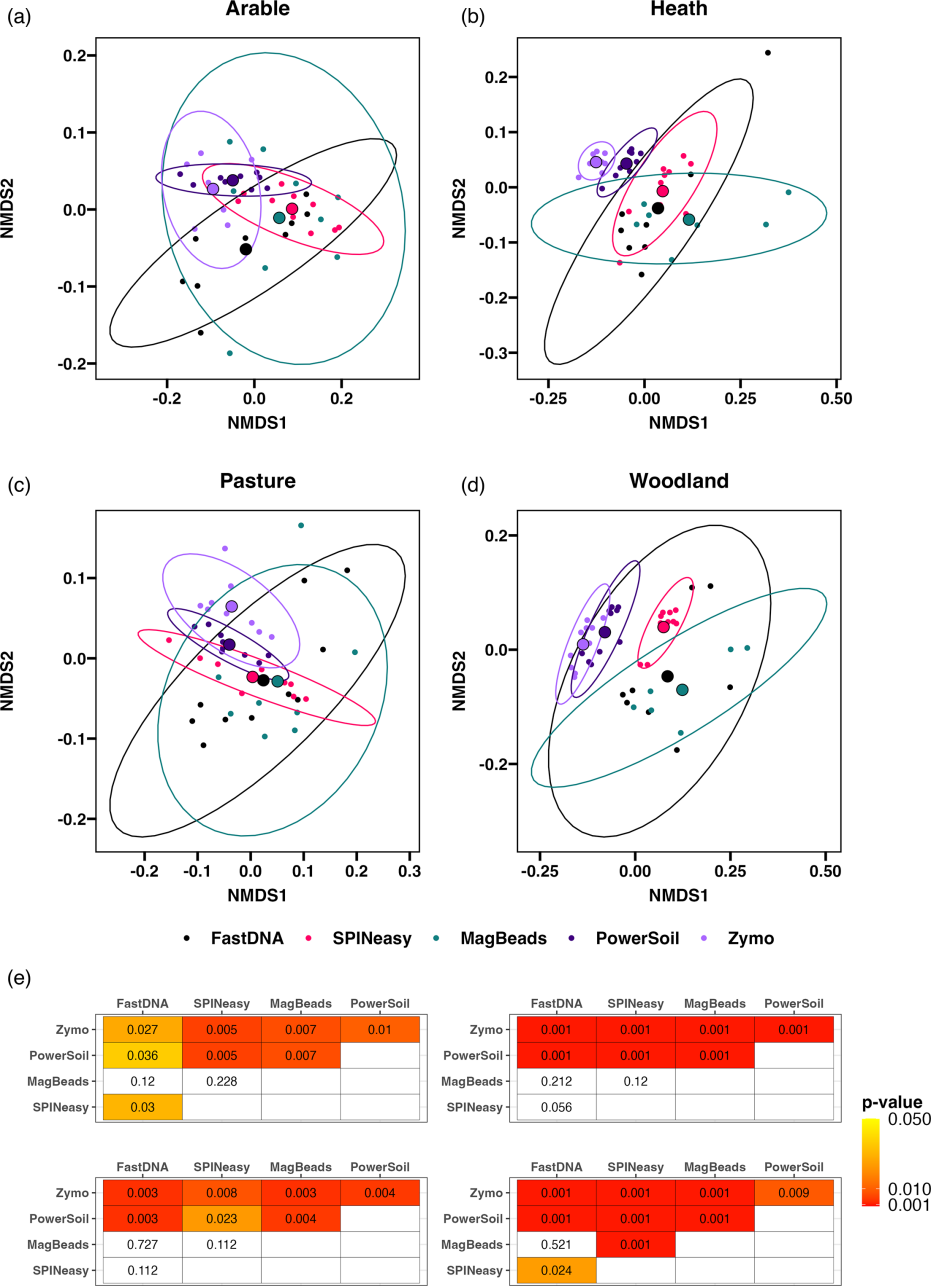
Extraction method drives variation in the measured microbial community composition within each soil type. Nonmetric Multidimensional Scaling (NMDS) plot based on Bray-Curtis distances between samples from (a) arable (stress=0.08), (b) heath (stress=0.11), (c) pasture (stress=0.11) and (d) woodland soil (stress=0.05), generated on count data from reads assigned to bacteria, archaea and fungi. (e) Benjamini-Hochberg corrected *P*-values are displayed from pairwise PERMANOVA comparing different extraction kits.

Beta diversity analysis within each soil type identified significant variation between microbial communities extracted with different kits (Fig. 7; PERMANOVA; arable *R*^2^=0.34, *P*<0.001; heath *R*^2^=0.43, *P*<0.001; pasture *R*^2^=0.29, *P*<0.001; woodland *R*^2^=0.55, *P*<0.001). Further investigation of pairwise comparisons found significant differences between both the PowerSoil and Zymo with all other kits in the study across all soil types (Fig. 7e). Otherwise, fewer significant pairwise differences were identified between the three MPBio kits, other than between the SPINeasy and FastDNA kits in the arable samples, and the SPINeasy kit with both the FastDNA and MagBeads kits in woodland samples.

Beta diversity ordinations indicated there to be considerable differences in the variability of microbial communities between kits, which could influence the differences identified by permutation analysis. Analyses of multivariate homogeneity of group dispersions within each extraction kit was carried out. Dispersion of samples from the PowerSoil and Zymo kits was consistently lower than other kits across all soil types (Fig. S7a-d), with significantly lower dispersion than both the FastDNA and MagBeads kits in the heath soil (Fig. S7b). Furthermore, PowerSoil, Zymo and SPINeasy kits all had significantly lower dispersion than the FastDNA kit for woodland soil samples (Fig. S7d). However, for the arable and pasture soils, dispersion was only significantly lower in the PowerSoil compared to FastDNA samples (Fig. S7a and c). These results suggest that differences in microbial communities identified between extraction kits are in part due to higher variability in the FastDNA and MagBeads kits, while supporting the conclusion that the PowerSoil kit provides the most consistent representation of the microbial community across replicates of all soil types.

To investigate whether presence-absence of microbial taxa is contributing to diversity between kits, beta diversity analysis was carried out using the Jaccard index on a presence-absence matrix of taxa across samples. Less significant variability in the presence-absence of taxa was observed for the arable and pasture soils compared with heath and woodland samples (Fig. S8). PowerSoil samples were significantly different from all other extraction kits in arable soils, while the PowerSoil and SPINeasy kits clustered away from the other kits in pasture samples (Fig. S8a, c and e). For both woodland and heath soils, samples from the Zymo, PowerSoil and SPINeasy kits clustered away from the MagBeads and FastDNA samples (Fig. S8b, d and e). Again, these differences were in part explained by significant variation in the group dispersions of the kits, with the PowerSoil displaying lower variation compared to other kits in all soils (Fig. S9a−d), and lower variation in the Zymo and SPINeasy kits in heath and woodland soil samples (Fig S8b and d). This indicates that there are significant differences in the presence-absence of microbial taxa between extraction kits. Furthermore, variability in community membership captured by replicate extractions of the same kit was higher for FastDNA and MagBeads kits, while the PowerSoil kit showed the most consistency in the microbial families identified.

**Fig. 8.**
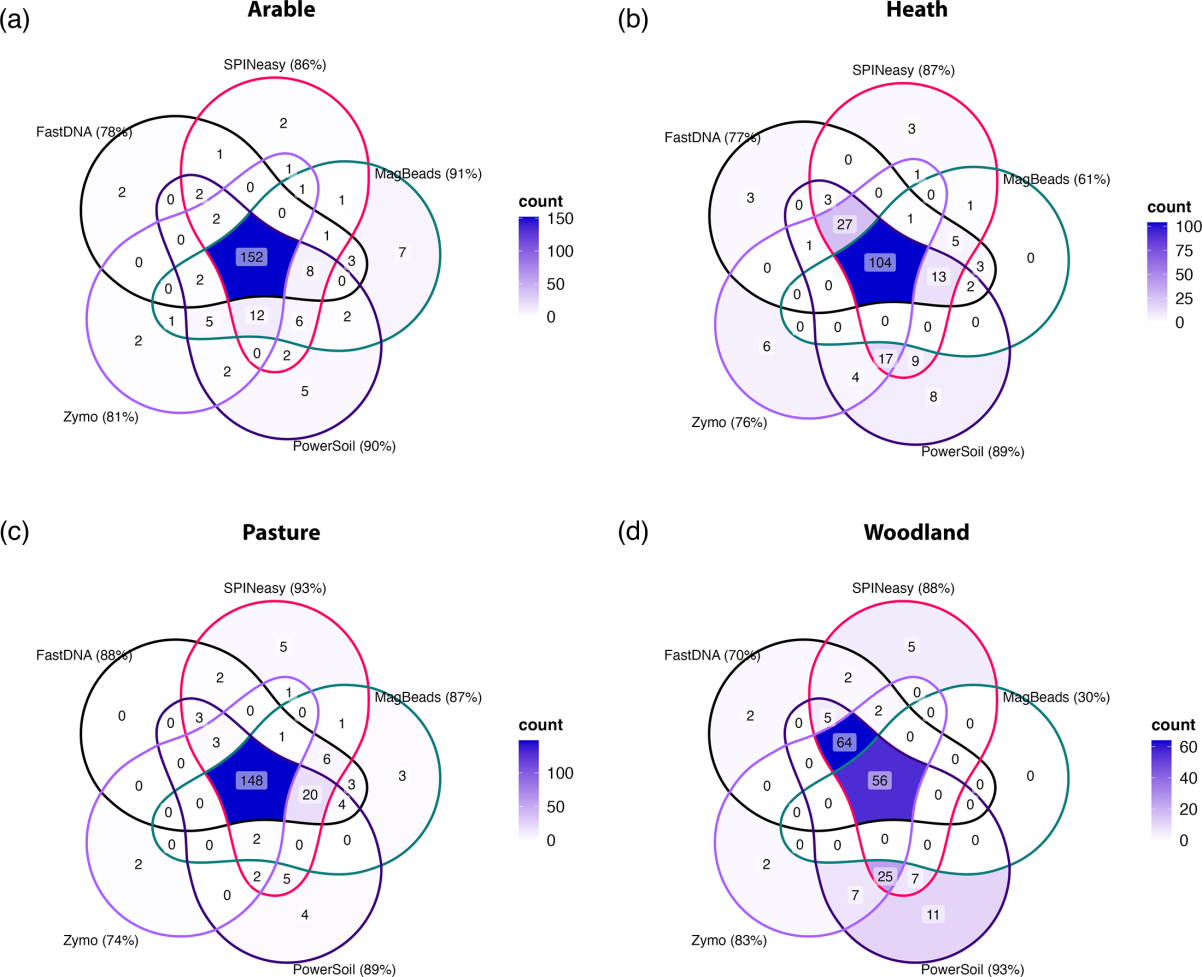
Extraction method impacts the microbial families detected by metagenomic sequencing. Venn diagrams showing crossover between the microbial families identified in samples extracted with each of the kits from (a) arable, (b) heath, (c) pasture and (d) woodland soil. The colour gradient used to fill each section represents the count of microbial families shared by the groups, with darker colours representing greater overlap in families identified between groups. Percentages represent the proportion of the total number of taxa identified in each soil type that were identified in samples from each extraction kit.

To account for variability between replicate extractions and investigate the capability of extraction kits to capture microbial diversity, microbial family counts were summed across replicates for each extraction kit within soil types and the presence-absence of each family was assessed. Overall, variability between extraction kits was lower for arable and pasture soils, with all kits capturing 68 and 69% of the microbial families identified, respectively, compared to 49 and 30% shared between all kits for the heath and woodland samples respectively (Fig. 8).

Higher variability in heath and woodland samples was largely caused by low family representation in MagBeads samples, in which they encompassed only 61 and 30% of microbial families, respectively, and to a lesser extent FastDNA and Zymo samples (Fig 8b and d). The PowerSoil kit consistently encompassed a high percentage of identified families across all soil types, along with the SPINeasy kit, which displayed slightly higher variation in the presence-absence of microbial families among replicates (Figs S8 and S9).

### The observed fungal community varies between extraction methods

**Fig. 9.**
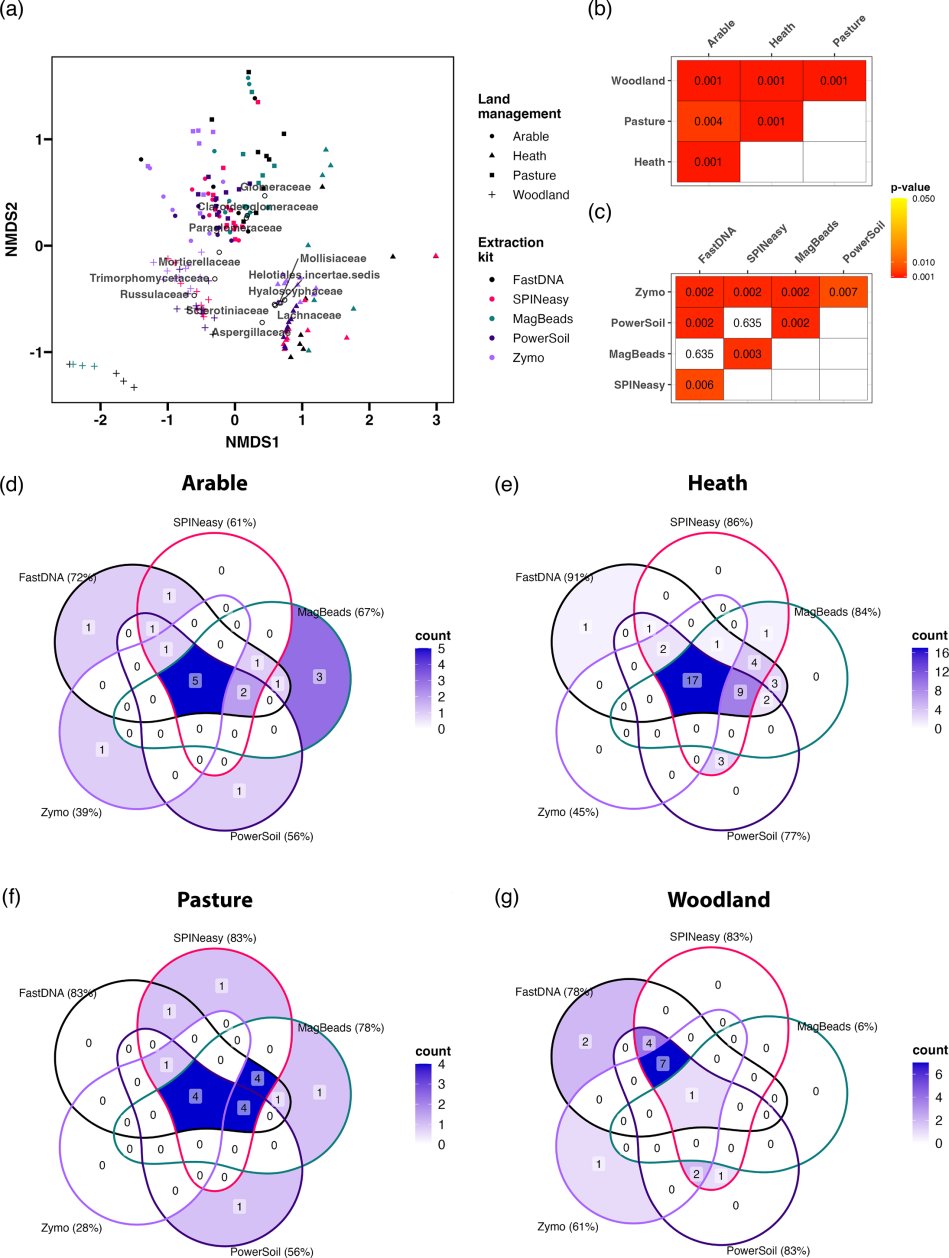
Extraction method impacts the representation of fungal communities in sequencing results. (**a**) Nonmetric Multidimensional Scaling (NMDS) plot based on Bray-Curtis distances between all samples, generated on fungal family counts. The top three microbial families that correlate with the extremes of each NMDS axis are displayed as black circles. The stress value for this analysis was 0.15. Benjamini-Hochberg corrected *P*-values are displayed from pairwise PERMANOVA comparing soil types (**b**) and extraction kits (**c**). Venn diagrams showing crossover between the fungal families identified in samples extracted with each of the kits from (**d**) arable, (**e**) heath, (**f**) pasture and (**g**) woodland soil. Percentages represent the proportion of the total number of taxa identified in each soil type that were identified in samples from each extraction kit.

Significant differences were identified in the fungal communities across soil types (Fig. 9a, b; PERMANOVA R^2^=0.62; *P*<0.001). These were driven by the dominance of ectomycorrhyzal genera of the family Russulaceae in woodland samples, the arbuscular mycorrhizal family Glomeraceae in pasture and arable samples, and various members of the order Helotiales in heath samples (Fig. 9a), largely from the *Hyaloscypha* genus known to associate with ericaceous roots [44]. Furthermore, there were significant differences between fungal communities identified by extraction kits (Fig. 9a and c; PERMANOVA *R*^2^=0.09; *P*<0.001), with significant pairwise differences between the FastDNA/MagBeads kits and all other extraction kits (Fig. 9c).

To investigate the impact of extraction method on fungal communities further, results from each soil type were analysed separately. The Zymo kit displayed consistently low representation of fungal families in all soil types (Fig. 9d–g), which was reflected in the significant difference in community composition compared to the all kits across the soil types based on Bray-Curtis distance (Fig. S10). Samples from arable, pasture and woodland soils, which contained low percentages of fungal reads (Fig. 5b), displayed high variability in community representation between extraction kits (Fig. 9d, f and g).

In particular, the MagBeads kit samples only contained one fungal family (Russulaceae) across all replicates for the woodland sample. Contrastingly, fungal family representation was much more consistent between extraction kits in heath soil samples (Fig. 9e), which had a higher fungal read percentage (Fig. 5b). These findings suggest that high variability in fungal communities identified by different extraction kits in arable, pasture and woodland soils could result from differential sampling of low abundance of fungal genomes in DNA samples. Furthermore, consistent with results from overall microbial community analysis, the variability between replicates was high for FastDNA and MagBeads samples across the soil types, while PowerSoil samples displayed more consistency in fungal community composition (Figs S10 and S11).

## Discussion

DNA extraction methods are known to influence the analysis of microbial communities using molecular techniques across multiple biomes, including the human gut [45], marine environments [46], wastewater [47] and soil [12]. Due to the heterogeneity of soil environments and their microbial communities, high spatial resolution of field sampling and sufficient biological replication within soil experiments is required. Along with the impact of environmental sample storage [10] and batch effects in metagenomics [48], these factors necessitate the reproducibility and efficient throughput of sample preparation.

DNA extraction kits were found to vary in their ability to detect microbial families, not just their relative abundance, which correlates with previous studies demonstrating the absence of up to seven bacterial phyla in extracts from some kits [12,49]. Variation in the detection of fungal families was particularly inconsistent between extraction kits, corroborating previous studies that found variability in alpha and beta diversity between extraction methods to be more pronounced in fungi than bacteria in environmental samples including soil [50,51]. Previous studies have found the use of enzymatic lysis techniques to improve the DNA yield from fungi in clinical samples [52,53], while some protocols utilise both enzymatic and mechanical methods sequentially to improve lysis of diverse and resistant cells [54]. Overall, these findings suggest that the choice of sample preparation method can dramatically impact the measured diversity of soil samples.

Furthermore, reproducibility of community structure between replicates has also been found to vary drastically between extraction methods in metabarcoding studies of soil [12,55]. In the present study, the consistency of technical replicates varied considerably between extraction kits across multiple factors, including read length, classification rate and observed microbial community, with the PowerSoil kit displaying the best reproducibility overall. Although the impact of extraction kit on the observed microbial diversity did not outweigh the variation identified between soil types here and in previous studies [56], inconsistency in DNA extraction method can impair the interpretation of treatment effects on microbial communities within experiments [51] and between studies on similar soil types. Therefore, care should be taken to trial DNA extraction methods prior to experiments on different soil types, and consideration given to the methods used in similar studies which may be included in comparative interpretation.

To maximise the benefits of long read sequencing for genome assembly, functional analysis and taxonomic classification in WGS metagenomics, the maintenance of DNA integrity during extraction is crucial. Although bespoke methods for extraction of high molecular weight DNA from soil exist [57], throughput and reproducibility favour the use of commercial kits. The fragment lengths of extracted DNA were found to vary considerably between the kits. Cell lysis through bead beating is common component of commercial soil nucleic acid extraction kits, as it enables reliable representation of microorganisms with tough cell surface structures [19]. However, bead beating leads to DNA fragmentation [19,58], creating a trade-off between cell lysis and DNA integrity. Similar to previous studies [58], the longer lysis time recommended for the Zymo kit led to highly fragmented DNA compared to other extraction methods which used the same homogenization conditions. Furthermore, the MagBeads kit resulted in the highest DNA fragment length, likely due to the lack of centrifuge column steps that are also known to fragment DNA. Interestingly, DNA extracted with the PowerSoil kit was significantly shorter than the SPINeasy samples, despite both kits following similar processes, suggesting a difference in the lysis matrix or purification column between the kits which impacts DNA fragmentation. These findings indicate that the extraction method used, including the lysis conditions and DNA purification process, has a dramatic impact on the fragment length of extracted DNA.

However, higher extracted DNA integrity did not consistently translate into longer sequencing reads, with many samples extracted with the FastDNA and Magbeads kits showing dramatic differences in read lengths compared to extracted DNA. Many processes involved in sample storage and library preparation can cause DNA fragmentation, including pipetting, vortexing and freeze-thaw cycles [59,60]. The average length of extracted DNA was found to correlate with the absolute loss of DNA fragment size in sequencing reads, which might suggest that longer fragments are more susceptible to fragmentation during library preparation. This may be due to physical shearing, although this has been demonstrated to mainly impact DNA fragments larger than those encompassed by samples in this study [59].

Alternatively, loss of fragment length may be caused during barcoding PCR. Humic substances from soil are thought to inhibit PCR through binding to both the template DNA and the DNA polymerase [18,61]. These inhibitors may therefore cause the truncation of PCR products through reduced polymerase efficiency or premature termination of DNA extension at inhibitor binding sites. Supporting this hypothesis, MagBeads and FastDNA samples showed the lowest PCR efficiencies, and samples from heath and woodland soils displayed a correlation between DNA purity and loss of DNA length. This could also explain the higher abundance or short unclassified reads, likely PCR artefacts, in MagBeads and FastDNA samples, as these may represent amplification of DNA not originating in the soil sample and therefore not bound to humic substances. Further investigation is required into the impacts of humic substances on amplification during long read library preparation, while consideration should be given to the use of PCR-free barcoding methods for long-read sequencing of environmental samples.

## Conclusions

In summary, commercial soil DNA extraction kits displayed varying suitability for long read WGS metagenomic sequencing across four soil types. DNA integrity varied between extraction methods, but extracted DNA length did not translate consistently into read length, with samples from some kits showing dramatic decreases in fragment size. This was associated with low taxonomic classification rate and inconsistent microbial community representation between replicates. Conversely, kits which displayed better retention of fragment length in sequencing reads gave more reproducible community composition, lending themselves to robust comparative analyses. Overall, for the soil types tested here, the QIAGEN DNeasy PowerSoil Pro Kit displayed the best suitability for reproducible long-read WGS metagenomic sequencing. Further investigation is required into the causes of loss in fragment length during library preparation, which is likely associated with impurities in soil DNA samples. Subsequent optimisation of additional DNA purification and library preparation may enhance the read lengths obtained from kits yielding superior integrity of extracted DNA. Finally, optimisation of DNA extraction method for soil long read metagenomics is recommended to enhance read length and reproducibility, particularly for soil types with extreme physico-chemical characteristics not represented here.

## supplementary material

10.1099/acmi.0.000868.v3 Uncited Supplementary Material 1.

## Abbreviations

HAYE: Hayes Wood
HPY: H. Pavers Yard
ITS: internal transcribed spacer
KNAP: Knapps Left 3^rd^
MUTT: mutters moor
NATMAP: National Soil Map of England and Wales
ONT: Oxford Nanopore Technologies
PCR: polymerase chain reaction
rRNA: ribosomal RNA
WGS: whole genome shotgun
