# Supplementary material for: Comparative evaluation of soil DNA extraction kits for long read metagenomic sequencing
Source: Access Microbiol. 2024 Sep 27;6(9):000868.v3. doi: 10.1099/acmi.0.000868.v3 (PMC11432601; doi:10.1099/acmi.0.000868.v3)
Supplement: Uncited Supplementary Material 1. [file acmi-6-00868-s001.pdf]

Comparative Evaluation of Soil DNA Extraction Kits for Long Read Metagenomic Sequencing – Supplementary Information

Harry T. Child[a], Lucy Wierzbicki[a], Gabrielle R. Joslin[a] & Richard K. Tennant[a#]

[a]Geography, Faculty of Environment, Science and Economy, Amory Building, Rennes Drive, Exeter, Devon, EX4 4RJ, UK.

Running title: Optimised soil DNA extraction for long read sequencing

#Address correspondence to Richard K. Tennant, R.K.Tennant@exeter.ac.uk

Keywords: Soil, long-read sequencing, metagenomics, Oxford Nanopore, DNA extraction, microbiome.

Data Availability

Individual sample SRA accession numbers are; SRR28415553, SRR28415554, SRR28415555, SRR28415556, SRR28415557, SRR28415558, SRR28415559, SRR28415560, SRR28415561, SRR28415562, SRR28415563, SRR28415564, SRR28415565, SRR28415566, SRR28415567, SRR28415568, SRR28415569, SRR28415570, SRR28415571, SRR28415572, SRR28415573, SRR28415574, SRR28415575, SRR28415576, SRR28415577, SRR28415578, SRR28415579, SRR28415580, SRR28415581, SRR28415582, SRR28415583, SRR28415584, SRR28415585, SRR28415586, SRR28415587, SRR28415588, SRR28415589, SRR28415590, SRR28415591, SRR28415592, SRR28415593, SRR28415594, SRR28415595, SRR28415596, SRR28415597, SRR28415598, SRR28415599, SRR28415600, SRR28415601, SRR28415602, SRR28415603, SRR28415604, SRR28415605, SRR28415606, SRR28415607, SRR28415608, SRR28415609, SRR28415610, SRR28415611, SRR28415612, SRR28415613, SRR28415614, SRR28415615, SRR28415616, SRR28415617, SRR28415618, SRR28415619, SRR28415620, SRR28415621, SRR28415622, SRR28415623, SRR28415624, SRR28415625, SRR28415626, SRR28415627, SRR28415628, SRR28415629, SRR28415630, SRR28415631, SRR28415632, SRR28415633, SRR28415634, SRR28415635, SRR28415636, SRR28415637, SRR28415638, SRR28415639, SRR28415640, SRR28415641, SRR28415642, SRR28415643, SRR28415644, SRR28415645, SRR28415646, SRR28415647, SRR28415648, SRR28415649, SRR28415650, SRR28415651, SRR28415652, SRR28415653, SRR28415654, SRR28415655, SRR28415656, SRR28415657, SRR28415658, SRR28415659, SRR28415660, SRR28415661, SRR28415662, SRR28415663, SRR28415664, SRR28415665, SRR28415666, SRR28415667, SRR28415668, SRR28415669, SRR28415670, SRR28415671, SRR28415672, SRR28415673, SRR28415674, SRR28415675, SRR28415676, SRR28415677, SRR28415678, SRR28415679, SRR28415680, SRR28415681, SRR28415682, SRR28415683, SRR28415684, SRR28415685, SRR28415686, SRR28415687, SRR28415688, SRR28415689, SRR28415690, SRR28415691, SRR28415692, SRR28415693, SRR28415694, SRR28415695, SRR28415696, SRR28415697, SRR28415698, SRR28415699, SRR28415700, SRR28415701, SRR28415702, SRR28415703, SRR28415704, SRR28415705, SRR28415706, SRR28415707, SRR28415708, SRR28415709, SRR28415710, SRR28415711, SRR28415712, SRR28415713, SRR28415714, SRR28415715, SRR28415716, SRR28415717, SRR28415718, SRR28415719, SRR28415720, SRR28415721, SRR28415722, SRR28415723, SRR28415724, SRR28415725, SRR28415726, SRR28415727, SRR28415728, SRR28415729, SRR28415730, SRR28415731, SRR28415732, SRR28415733, SRR28415734, SRR28415735, SRR28415736, SRR28415737, SRR28415738, SRR28415739, SRR28415740, SRR28415741, SRR28415742, SRR28415743, SRR28415744, SRR28415745, SRR28415746, SRR28415747, SRR28415748, SRR28415749, SRR28415750, SRR28415751, SRR28415752, SRR28415753, SRR28415754, SRR28415755, SRR28415756, SRR28415757, SRR28415758, SRR28415759, SRR28415760, SRR28415761, SRR28415762, SRR28415763, SRR28415764, SRR28415765, SRR28415766, SRR28415767, SRR28415768, SRR28415769, SRR28415770,

Figure S1 Rarefaction curves for microbial families identified in each sample at increasing subsampled read depths

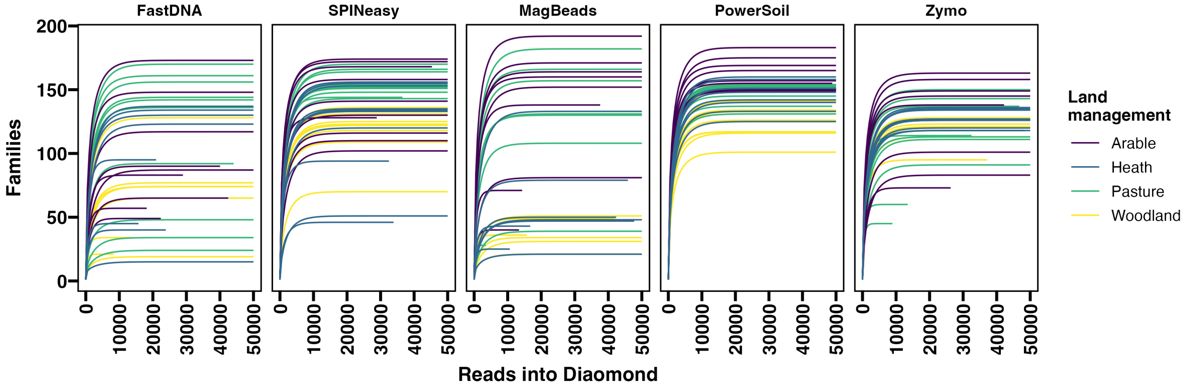

Figure S2 DNA integrity numbers of extracted DNA. Boxplots of DNA integrity numbers (DINs) measure for extracted DNA samples. Coloured points show data points from arable (purple), heath (blue), pasture (green) and woodland (yellow) soil types. P-values are given for the results of a Kruskal-Wallis test across all groups, as well as significant pairwise comparisons using Wilcoxon signed-rank test (*: p <= 0.05; **: p <= 0.01; ***: p <= 0.001; ****: p <= 0.0001).

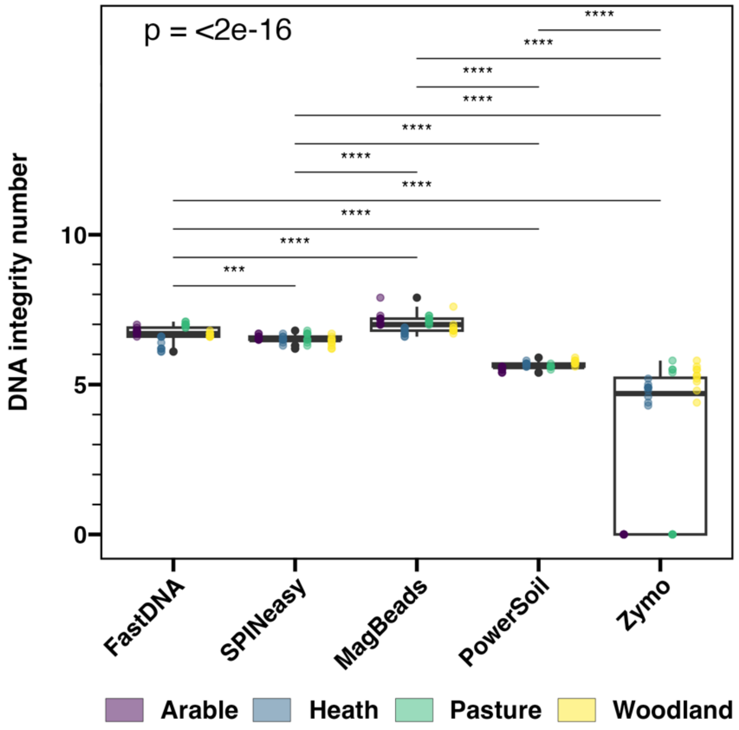

Figure S3 **Extracted DNA yield, purity and integrity separated by soil type.** Boxplots of (a) DNA yield and (b-c) purity, calculated by the absolute difference between the A260:A280 and A260:A230 ratios of the sample from 1.8 and 2, respectively, as well as (d) DNA integrity number and (e) average DNA length calculated on the TapeStation traces, separated by soil type. P-values are given for the results of a Kruskal-Wallis test across all groups, as well as significant pairwise comparisons using Wilcoxon signed-rank test (*: p <= 0.05; **: p <= 0.01; ***: p <= 0.001; ****: p <= 0.0001).

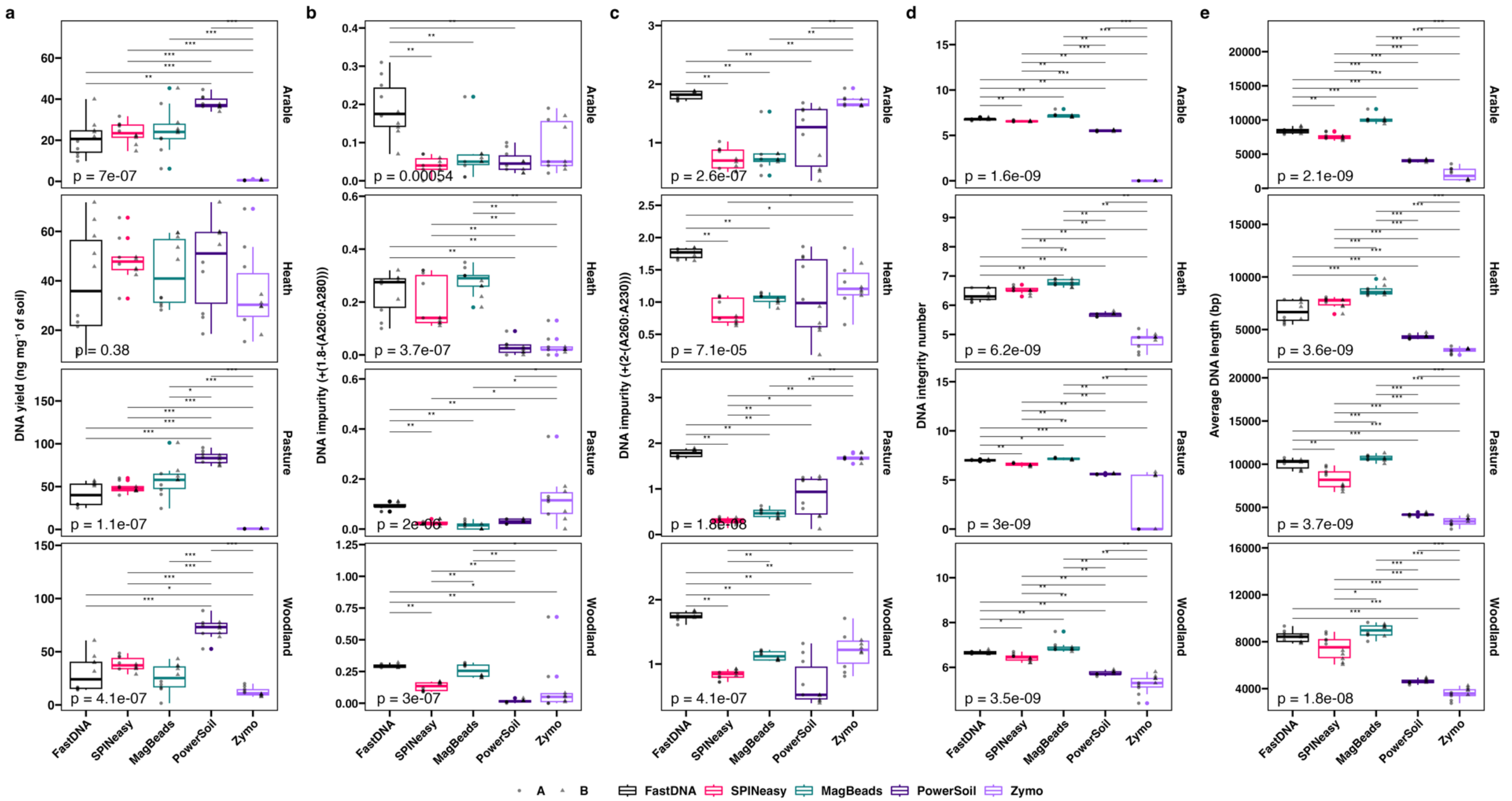

Figure S4 Impact of laboratory user on extracted DNA yield, purity and integrity. Boxplots of (a) DNA yield and (b-c) purity, calculated by the absolute difference between the 260:280 and A260:A230 ratios of the sample from 1.8 and 2, respectively, as well as (d) DNA integrity number and (e) average DNA length calculated on the TapeStation traces, across each extraction kit. Coloured points show data points from arable (purple), heath (blue), pasture (green) and woodland (yellow) soil types. P-values are given for the results of pairwise comparisons using Wilcoxon signed-rank test (*: p <= 0.05; **: p <= 0.01; ***: p <= 0.001; ****: p <= 0.0001).

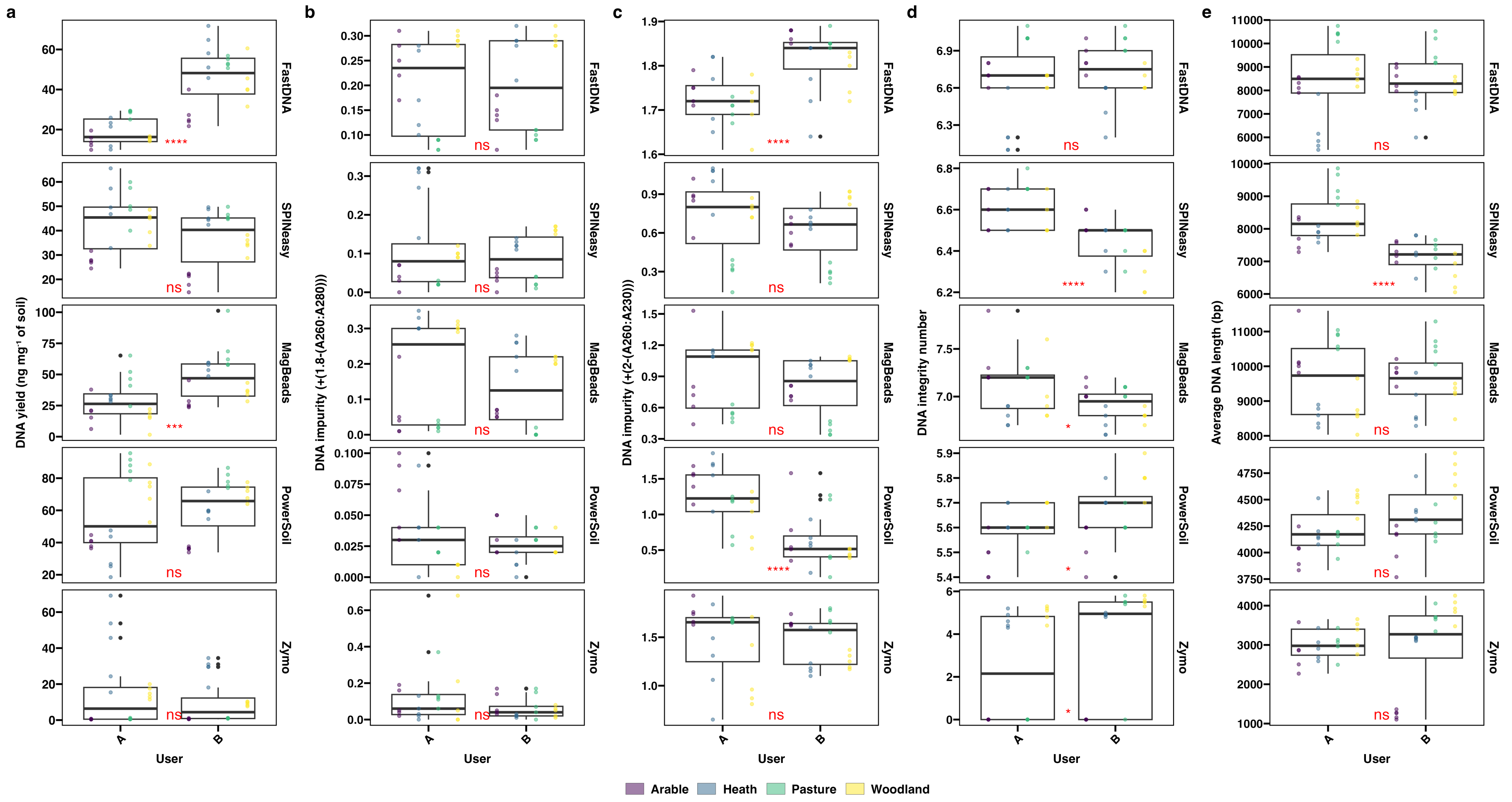

Figure S5 Impact of extraction kit on read lengths for samples of each soil type. Boxplots of (a) average read length and (b) the decrease in average length between extracted DNA and reads, separated by soil type. (c) Boxplot of the fold increase between total DNA input into barcoding PCR reaction and total DNA output. P-values are given for the results of a Kruskal-Wallis test across all groups, as well as significant pairwise comparisons using Wilcoxon signed-rank test (*: p <= 0.05; **: p <= 0.01; ***: p <= 0.001; ****: p <= 0.0001).

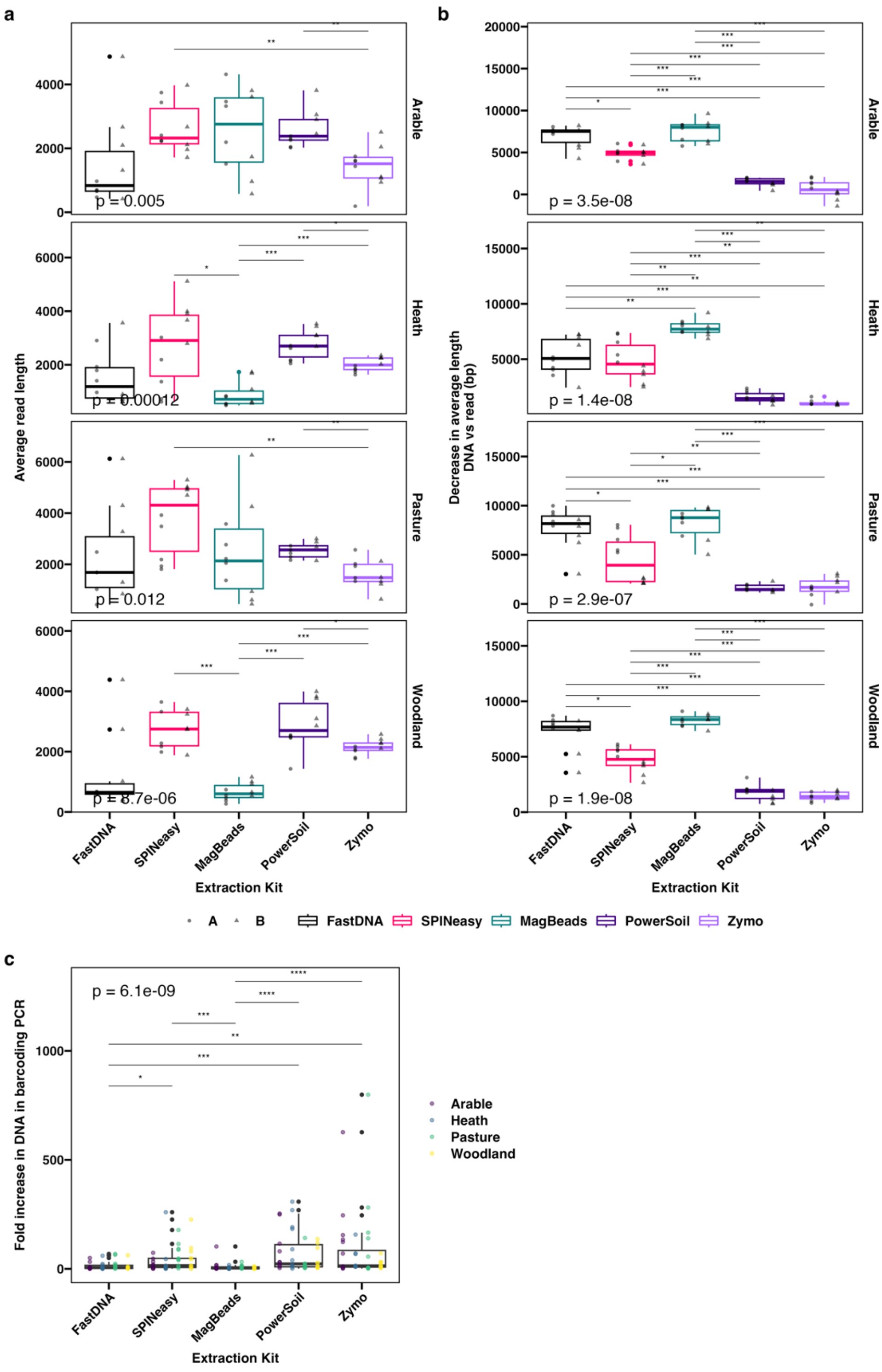

Figure S6 Relationship between DNA purity metrics and decrease in DNA fragment length between extracted DNA and reads. (a) Relationship between A260:A280 DNA purity and decrease in DNA fragment length with Pearson correlation coefficients calculated within each extraction kit. (b-c) Relationship between A260:A230 DNA purity and decrease in DNA fragment length, with Pearson correlation coefficients calculated overall and within each soil type.

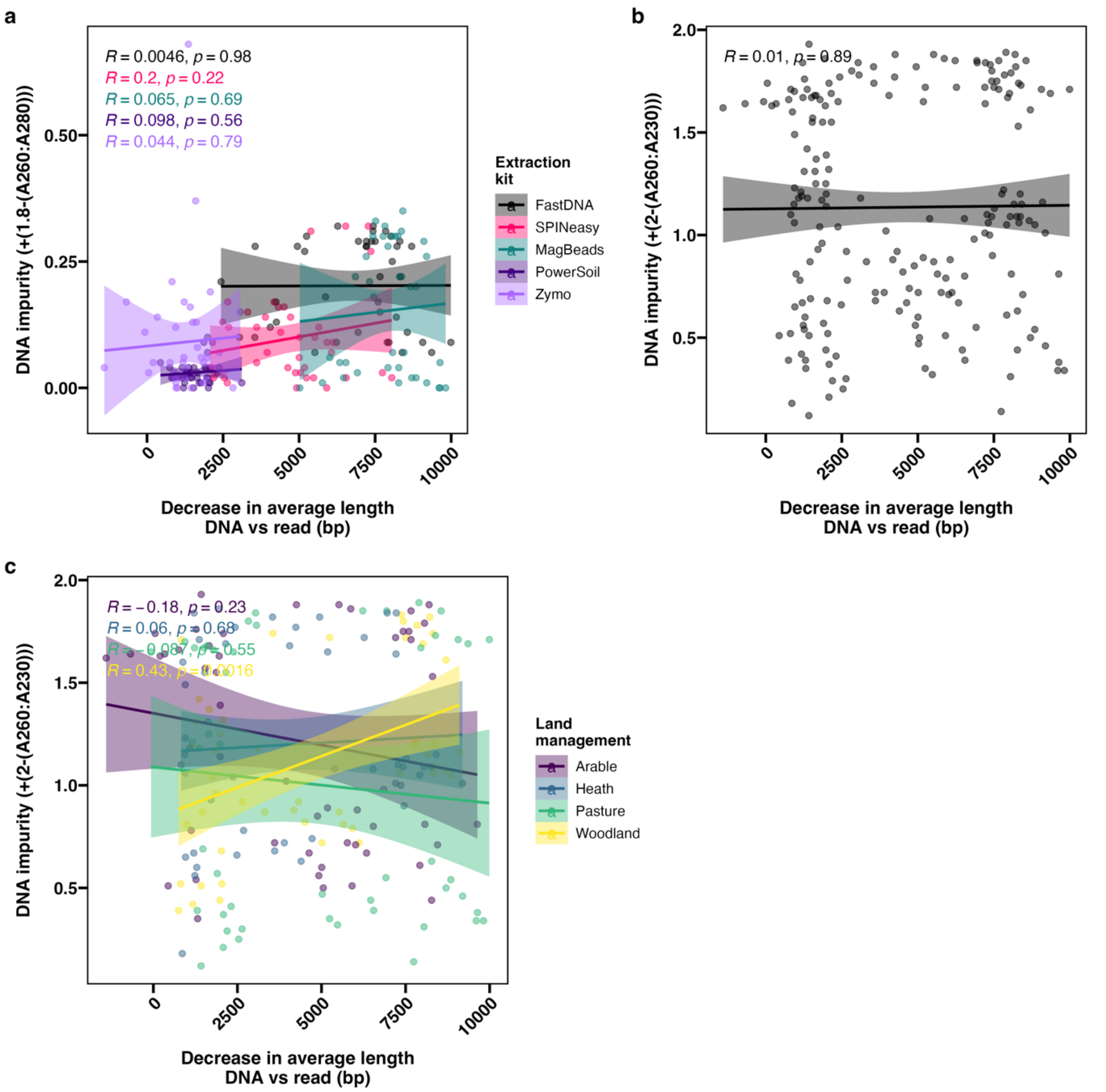

Figure S7 Beta dispersion of samples in NMDS ordinations of microbial community composition within each soil type, based on Bray-Curtis distances. Boxplots of distances from centroids for each extraction kit calculated using the *betadisper* function in the R package vegan, displayed for samples from (a) arable, (b) heath, (c) pasture and (d) woodland soils. P-values are given for the results of a Kruskal-Wallis test across all groups, as well as significant pairwise comparisons using Wilcoxon signed-rank test (*: p <= 0.05; **: p <= 0.01; ***: p <= 0.001; ****: p <= 0.0001).

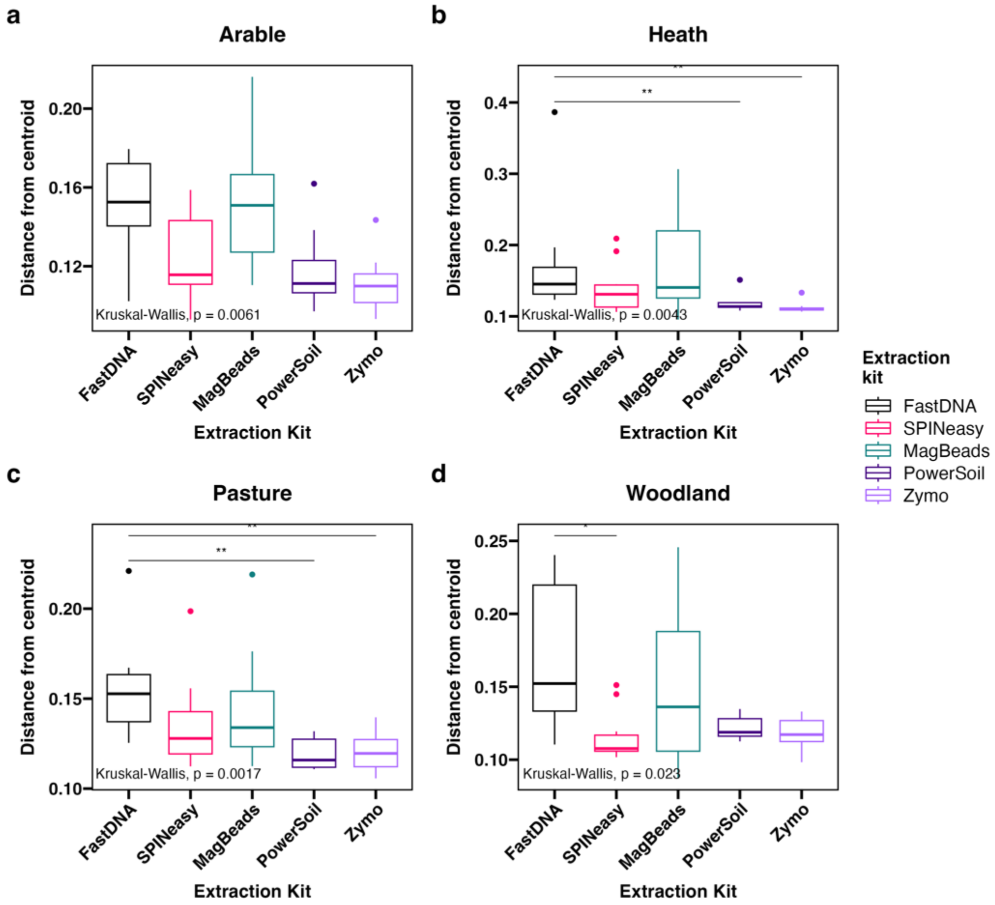

Figure S8 Extraction method drives variation in the microbial community identified in each soil type. Nonmetric Multidimensional Scaling **(**NMDS) plot based on Jaccard distances between samples from (a) arable (stress = 0.07), (b) heath (stress = 0.07), (c) pasture (stress = 0.07) and (d) woodland (stress = 0.08) soil, generated on count data from reads assigned to bacteria, archaea and fungi. (e) Benjamini-Hochberg corrected p-values are displayed from pairwise PERMANOVA comparing different extraction kits.

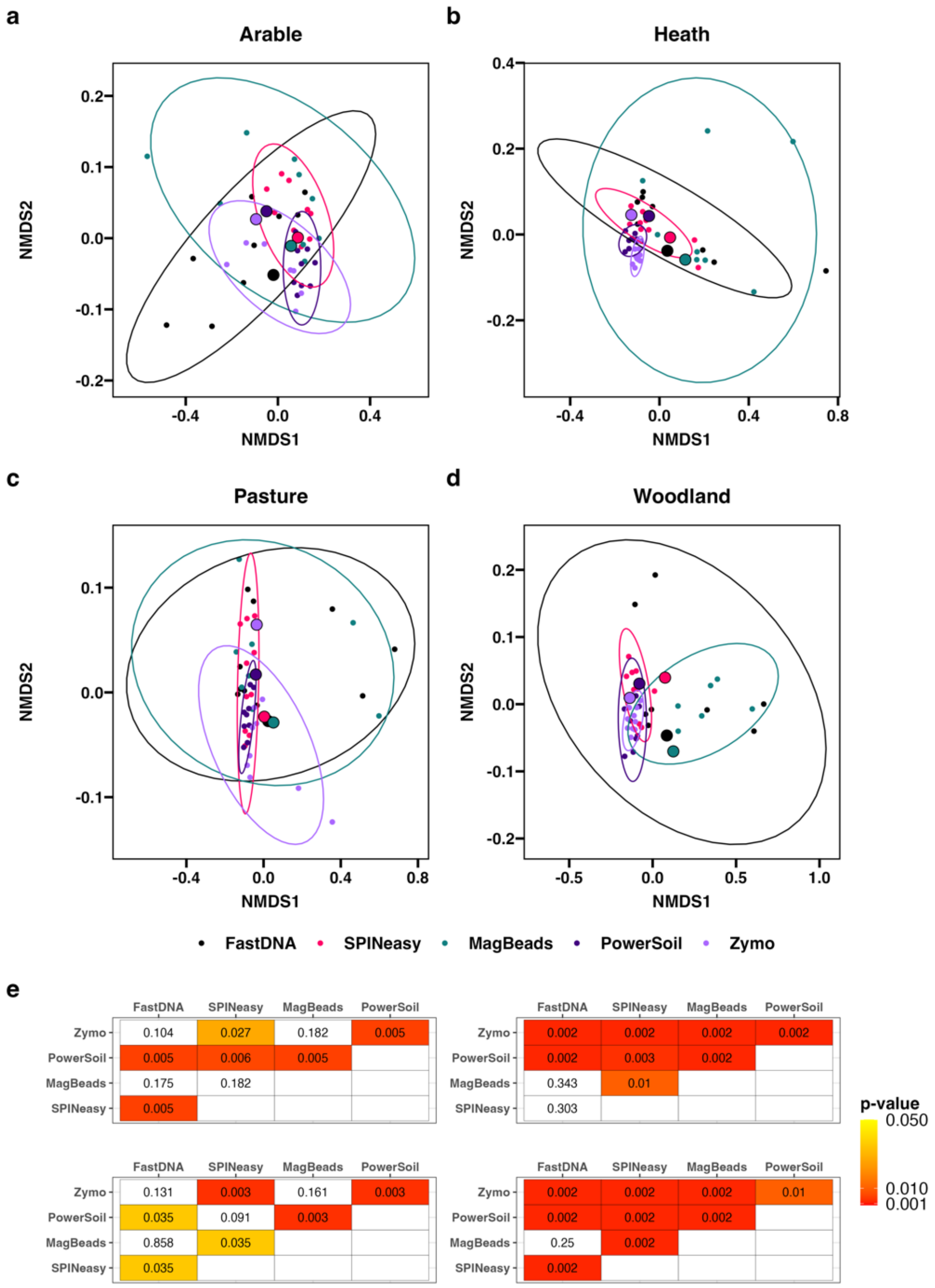

Figure S9 Beta dispersion of samples in NMDS ordinations of microbial community composition within each soil type, based on Jaccard distances. Boxplots of distances from centroids for each extraction kit calculated using the *betadisper* function in the R package vegan, displayed for samples from (a) arable, (b) heath, (c) pasture and (d) woodland soils. P-values are given for the results of a Kruskal-Wallis test across all groups, as well as significant pairwise comparisons using Wilcoxon signed-rank test (*: p <= 0.05; **: p <= 0.01; ***: p <= 0.001; ****: p <= 0.0001).

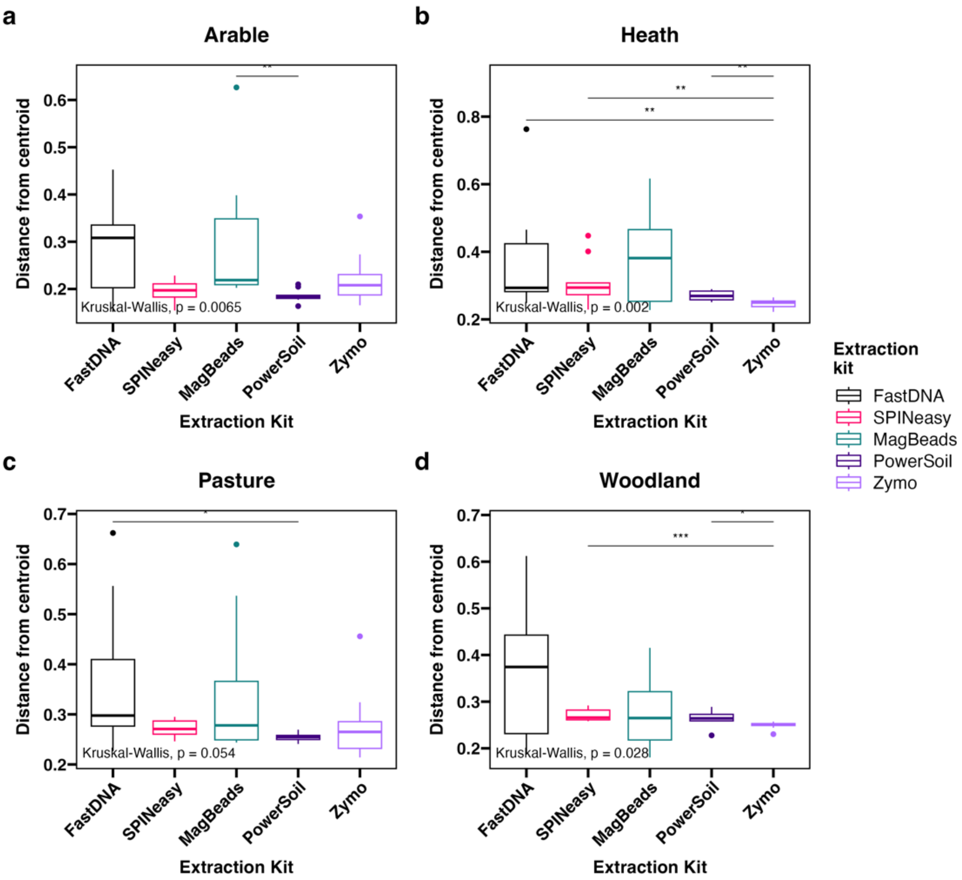

Figure S10 Extraction method drives variation in the measured fungal community composition within each soil type. Nonmetric Multidimensional Scaling **(**NMDS) plot based on Bray-Curtis distances between samples from (a) arable (stress = 0.1), (b) heath (stress = 0.04), (c) pasture (stress = 0.09) and (d) woodland (stress = 0.07) soil, generated on count data from reads assigned fungi. (e) Benjamini-Hochberg corrected p-values are displayed from pairwise PERMANOVA comparing different extraction kits.

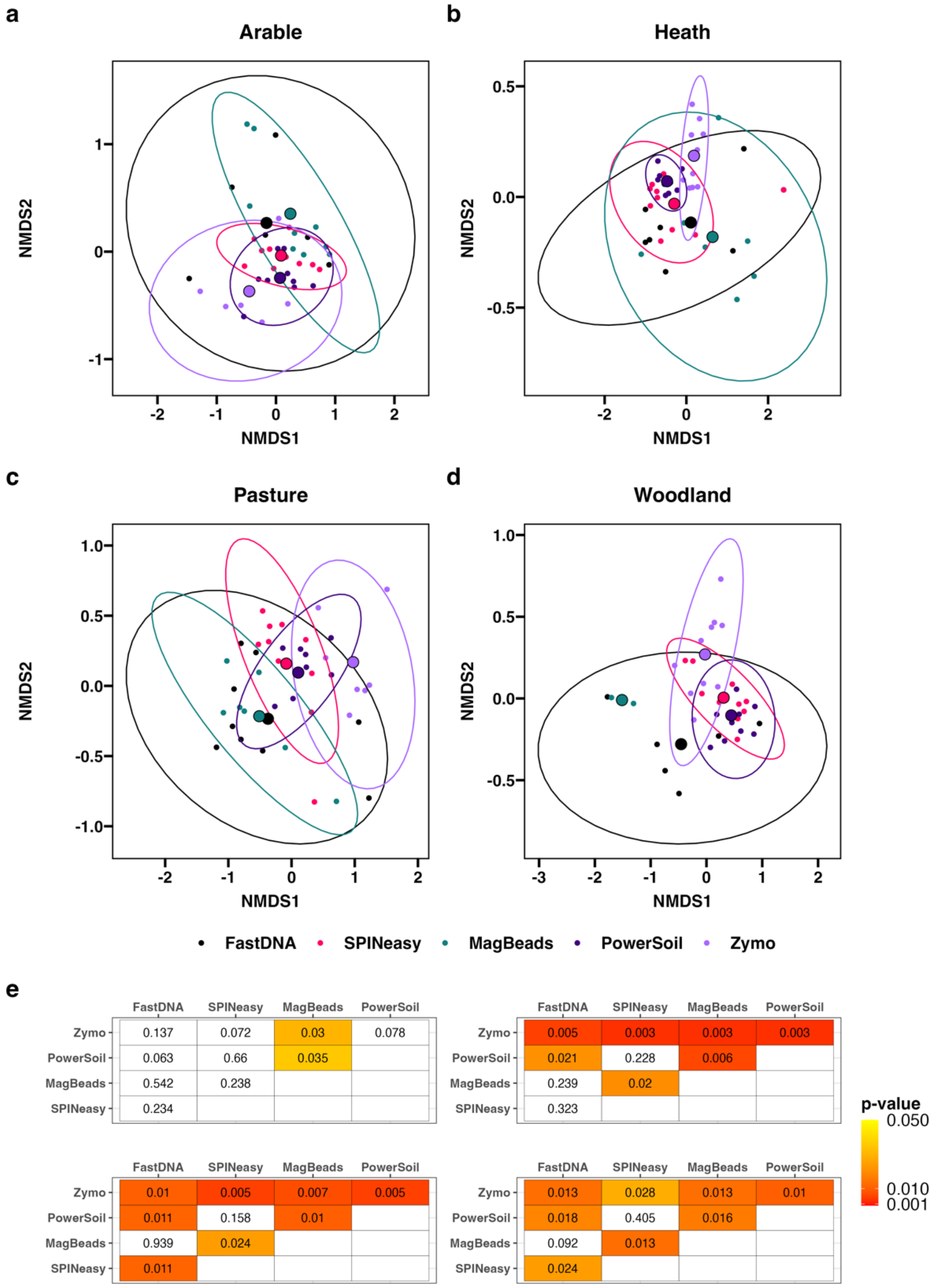

Figure S11 Beta dispersion of samples in NMDS ordinations of fungal community composition within each soil type, based on Bray-Curtis distances. Boxplots of distances from centroids for each extraction kit calculated using the *betadisper* function in the R package vegan, displayed for samples from (a) arable, (b) heath, (c) pasture and (d) woodland soils. P-values are given for the results of a Kruskal-Wallis test across all groups, as well as significant pairwise comparisons using Wilcoxon signed-rank test (*: p <= 0.05; **: p <= 0.01; ***: p <= 0.001; ****: p <= 0.0001).

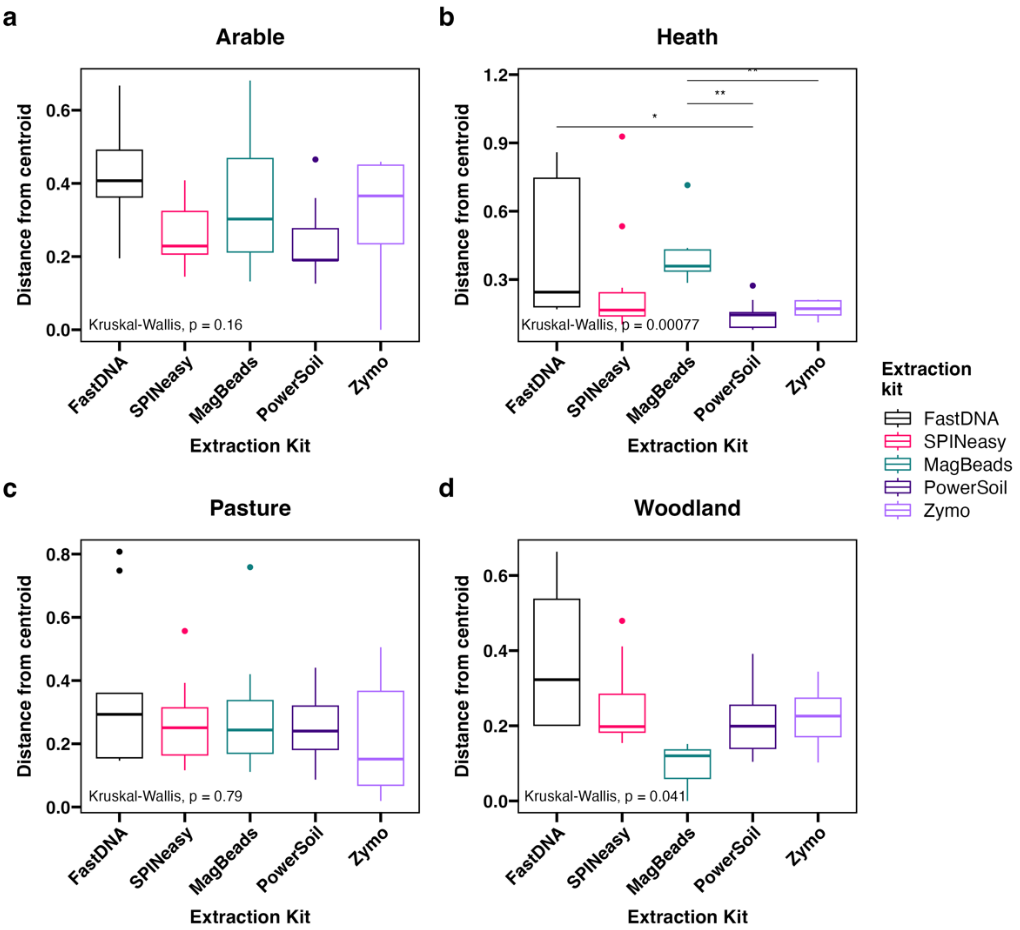

MagBeads FastDNA™ Kit for Soil

Cat. No.:116561050 (50 preps) & 116561005 (5 preps)

Quick-Start Protocol

Revision 1.0 Mar 2021

Notes before starting

- ☐ Store Magnetic Beads at 2 - 8 °C upon arrival; do not freeze.
- ☐ Expect precipitation in Lysis Buffer S1; warming the solution to 55 °C will help dissolve the precipitates.
- ☐ Add 35 mL isopropanol to Binding Buffer MS and mark on the bottle.
- ☐ Add 50 mL 100% ethanol to Wash Buffer S and mark on the bottle.
- ☐ Vortex the sample in a Lysing Matrix E tube at maximum speed for 10 mins if a FastPrep® Instrument is unavailable. Secure samples on the vortex through an adapter to ensure homogenization.
- ☐ Centrifugation speed stated in the manual will be a guideline; use the maximum speed available if 14,000 x g is not feasible.

Manual Extraction

Lyse

1 Add 100 - 500 mg soil sample to a **Lysing Matrix E** tube.
Note: After adding the sample to the tube, ensure there is still 1/3 - 1/4 empty space remaining in the tube.

2 Add 980 uL **Lysis Buffer S1**, 120 μL **Lysis Buffer S2** and 10 μL **RNase A Solution** to the sample in the Lysing Matrix E tube and vortex 5 - 10 seconds to mix.

3 Homogenize sample in a FastPrep® Instrument for 20 - 40 seconds at a speed setting of 6.0 m/s.
Note: The speed and time can be changed according to different soil samples. Vortex 5 - 10 mins at maximum speed if a FastPrep® Instrument is not available. If homogenizers from other manufacturers are used, consult the Instruction Manual or manufacturer for appropriate homogenization parameters.

4 Centrifuge at 14,000 x g for 5 mins to pellet debris.

Purify

5 Transfer the supernatant (~800 μL) to a clean 1.5 mL microcentrifuge tube. Add 250 μL **Inhibitor Removal MS** and mix by inverting the tube 20 times.

6 Centrifuge at 14,000 x g for 5 mins to pellet precipitate.

Bind

7 Transfer the supernatant (~800 μL) to a new 2.0 mL microcentrifuge tube. Add an equal volume of **Binding Buffer MS** and 5 μL **Magnetic Beads** to the supernatant. Vortex or invert the tube to mix.
Note: Ensure Magnetic Beads are thoroughly mixed before transferring to the supernatant.

8 Place the tube on a shaker for 5 mins to allow binding.

9 Place the tube on a magnetic rack for 3 - 5 mins, allow Magnetic Beads to settle, then discard supernatant.
Note: If the supernatant is too turbid or Magnetic Beads are attracting slowly, extend attraction time.

Wash

10 Add 800 μL **Wash Buffer S** to the tube and place on the shaker for 3 mins.

11 Place the tube on the magnetic rack for 1 min, allow Magnetic Beads to settle, then discard supernatant.

12 Repeat step 10 to step 11 for a second wash step.

13 Air dry Magnetic Beads for 5 - 10 mins at 55 ℃ by placing the tube on a heat block.
Note: This is for removal of residual ethanol and ensure Magnetic Beads are completely dry.

Elute

14 Add 100 μL **DES Buffer** to resuspend Magnetic Beads and incubate on a heat block at 55 ℃ for 5 mins.

15 Place the tube on a magnetic rack for 3 - 5 mins until Magnetic Beads have settled, and transfer the supernatant (eluted DNA) to a clean 1.5 mL microcentrifuge tube. DNA is now ready for PCR and other downstream applications. Store at - 20 ℃ for extended periods.
Note: If the supernatant is too turbid or there are still Magnetic Beads remaining, please centrifuge at 14,000 x g for 3 - 5 mins and transfer the supernatant again.

MagBeads FastDNA™ Kit for Soil

Cat. No.:116561050 (50 preps) & 116561005 (5 preps)

Quick-Start Protocol

Revision 1.0 Mar 2021

Automated Extraction

Lyse

1 Add 100 - 500 mg soil sample to a **Lysing Matrix** E tube.
Note: After adding the sample to the tube, ensure there is still 1/3 - 1/4 empty space remaining in the tube.

2 Add 980 μL **Lysis Buffer S1**, 120 μL **Lysis Buffer S2** and 10 μL **RNase A Solution** to the sample in the Lysing Matrix E tube and vortex 5 - 10 seconds to mix.

3 Homogenize sample in a FastPrep® Instrument for 20 - 40 seconds at a speed setting of 6.0 m/s.
Note: The speed and time can be changed according to different soil samples. Vortex 5 - 10 mins at maximum speed if a FastPrep® Instrument is not available. If homogenizers from other manufacturers are used, consult the Instruction Manual or manufacturer for appropriate homogenization parameters.

4 Centrifuge at 14,000 x g for 5 mins to pellet debris.

Purify

5 Transfer the supernatant (~800 μL) to a clean 1.5 mL microcentrifuge tube. Add 250 μL **Inhibitor Removal MS** and mix by inverting the tube 20 times.

6 Centrifuge at 14,000 x g for 5 mins to pellet precipitate.

Auto Bind, Wash & Elute

7 Transfer 400 μL supernatant to well 2 and well 3 of a 96 - well plate. Add additional reagents into the respective wells as shown below.

Well	Reagents	Volume (μL)
1	Deionized water	800
	Magnetic Beads	5
2	Sample supernatant	400
	Binding Buffer MS	400
3	Sample supernatant	400
	Binding Buffer MS	400
4	Wash Buffer S	800
5	Wash Buffer S	800
6	DES Buffer	100

8 Run the instrument according to following settings.

Step	Well	Process	Time (s) Mix	Time (s) Wait	Time (s) Attract	Mixing Speed	Temp (°C)
1	1	Magnetic Beads Preparation	60	0	120	Medium	RT
2	2	Bind	300	0	120	Medium	RT
3	3	Bind	300	0	120	Medium	RT
4	4	Wash 1	180	0	120	Medium	RT
5	5	Wash 2	180	0	120	Medium	RT
6	5	Dry	0	600	0	-	RT
7	6	Elute	300	0	150	Medium	55

9 Transfer eluted DNA from well 6 into a clean 1.5 mL microcentrifuge tube. DNA is now ready for PCR and other downstream applications. Store at -20℃ for extended periods.
Note: If there are still Magnetic Beads remaining in eluted DNA, please centrifuge at 14,000 x g for 3 - 5 mins and transfer the supernatant again.

Order Information

Product	Package	Cat. No.
MagBeads FastDNA™ Kit for Soil	50preps	116561050
FastPrep - 24™ 5G Instrument Including 24 x 2 mL samples adapter	1ea	116005500
MP Magnetic Rack 24	1ea	116570413
MP Magnetic Rack 8	1ea	116570426

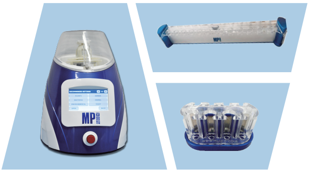

Instruction Manual
Research Use Only

SPINeasy DNA Kit for Feces / Soil

For the isolation of genomic DNA from fecal and soil samples

Size: 50 and 5 preps
Storage: 15-25 °C
Cat. No.:116547050 (50 preps) / 116547000 (5 preps)
Content Version: July 2022

www.mpbio.com

Table of Contents

1. Introduction to SPINeasy DNA Kit for Feces / Soil 3
2. Kit Components and User Supplied Materials 4
3. Storage and Kit Stability 5
4. Important Consideration Before Use 5
5. Safety Precautions 5
6. Protocol 6
7. Flow Chart 8
8. Data 9
9. Troubleshooting 11
10. Product Use Limitation & Warranty 13

1. Introduction to SPINeasy DNA Kit for Feces /Soil

Soil and fecal samples display distinct processing challenges. The composition of fecal samples is largely dependent on diet which includes fibers, undigested particle, bilirubin, complex polysaccharides, and lipids. Those compounds impair the sample homogenization, decrease both the quantity and quality of the DNA extracted. Conversely, soil samples often contain a large amount of inhibitory compounds such as humic acid, heavy metals, and other aromatic components which may be co-purified with the DNA and interfere with downstream applications.

The SPINeasy DNA Kit for Feces / Soil was designed to tackle these challenges by providing an efficient way for rapidly isolating high-quality DNA from both feces and soil samples. Irrespective of their complexities, all samples are optimally homogenized by bead beating with the new Lysing Matrix YB and lysis Buffer SF1. Subsequent treatment with Buffer SF2 effectively removes humic acid and other contaminants. The chemistry included in Buffer SF3 enables the specific binding of DNA without co-purification of RNA, eliminating the need for RNase A treatment. DNA obtained from heavily contaminated soil samples showed no inhibition in PCR and was immediately ready to be used for downstream applications, including long fragment PCR, qPCR, and next-generation sequencing (16S and whole genome) without the need for a further inhibitor removal step.

Visit www.mpbio.com to explore additional products to support your research.

Kit Specifications at A Glance

Technology	Silica membrane technology
Format	Mini spin column
Vacuum manifold	No
Sample	Fecal (omnivore/ herbivore/ carnivore diets) and soil
Sample amount	250mg
Typical yield	up to 50ug (sample dependent)
Elution volume	30–100 µL
Preparation time	<30 min (6 preps)

2. Kit Components and User Supplied Materials

2.1 SPINeasy DNA Kit for Feces /Soil Component

Product	50 reactions (Cat. No.116547050)		5 reactions (Cat. No.116547000)	
	Package	Cat. No.	Package	Cat. No.
Lysing Matrix YB	50 ea	116547051	5 ea	116547001
Buffer SF1	45 mL	116547052	4.5 mL	116547002
Buffer SF2	12 mL	116547053	1.2 mL	116547003
Buffer SF3	35 mL	116547054	3.5 mL	116547004
Buffer SF4	40 mL	116547055	4 mL	116547005
Buffer SF5	40 mL	116547056	4 mL	116547006
Buffer SF6	7.5 mL	116547057	1 mL	116547007
Column SF	50 ea	116547058	200 ea	116547008
2mL Collection Tubes	100 ea	116543059	10 ea	116543009
1.5 mL microcentrifuge tubes	50 ea	116547060	5 ea	116547010
Quick-start Protocol	1 ea	-	1 ea	-
Instruction Manual	Available www.mpbio.com	-	Available www.mpbio.com	-
MSDS & CoA	Available www.mpbio.com	-	Available www.mpbio.com	-

2.2 User Supplied Materials

- FastPrep® Instrument - FastPrep-24™ 5G (Cat. No.116005500) or vortex mixer
- Microcentrifuge (speed ≥ 15,000 x g)
- Single-channel pipettors (2 µL–1000 µL)
- Nuclease-free tips

3. Storage and Kit Stability

All the SPINeasy DNA Kit for Feces / Soil components are guaranteed for 12 months upon receipt when stored at room temperature (15-25˚C). For extended storage, store the Column SF between 4-8˚C to maintain its performance. Column SF stored above 25 ˚C may result in reduced performance. The Buffer SF2 should be stored at 4-8°C upon arrival.

4. Important Consideration Before Use

- ❑ Store the Buffer SF2 at 4-8˚C once received.
- ❑ A centrifuge able to generate at least 15,000 g is required to obtain optimal results. For step 2 (homogenization) and 3 (contaminant removal), perform centrifugation at the highest speed available.
- ❑ It is not recommended to use this kit with a vacuum manifold as this may decrease the overall purity.
- ❑ The SPINeasy DNA Kit for Feces / Soil has been optimized to process 250 mg of sample. Larger sample amount (up to 450-500 mg) can be processed with the kit to obtain higher yield (+30-40%). However, this may affect the eluent purity depending on sample complexity.
- ❑ The samples can be transferred from tube to tube by pouring during the homogenization and binding procedure to reduce plastic waste. Alternatively, pipette tips can be used to maximise recovery.

5. Safety Precautions

Wear personal protective equipment (gloves, lab coat and eye protection) to prevent contact with the skin or mucous membranes. Consult the Material Safety Data Sheet at www.mpbio.com for additional details. The Buffer SF2 contains components that may cause irritation when in contact with human tissue. Buffer SF3 and Buffer SF4 include chaotropic salts, which can form highly reactive compounds when combined with bleach. Buffer SF4 and Buffer SF5 are flammable.

6. Protocol

Weigh up to 250mg of the soil or feces and add it to a Lysing Matrix YB tube.

Note: For hard and large stool samples, such as those obtained from goat, use a mortar or a pestle to fractionate the sample into small particles prior to addition to the Lyisng Matrix YB tube.

1. Homogenization

- Add **800 µL of Buffer SF1**. Homogenize using a **Fastprep® at 5m/s for 35 sec** or **vortex at 2500-3000rpm for 20min**.

  Note: The performance of the DNA output (yield, purity and DNA integrity) obtained using a vortex is highly dependent on the model of vortex used. The condition stated above can be used as starting point. The homogenization time and speed should be optimized by the user. Avoid using tape or holding the matrix tubes with your hands, which can result in reduced homogenization efficiency, inconsistent results, and reduced yields.

- Centrifuge for **2 min** @ ≥**15,000 g**.

  Note: For herbivore feces enriched in plant particles like grass and seeds, increase the centrifugation speed (≥17, 000 g) and/or time (2-5min).

2. Inhibitor removal

- Transfer the supernatant (~500-600µL) into 2mL centrifuge tube (provided).

  Optional: The matrix tube may be re-centrifuged after transfer of the supernatant, using a tip to recover any additional lysate.

- Add **200µL** of **Buffer SF2** to precipitate contaminants, vortex for 1 sec and centrifuge for **1min** @ ≥ **15,000 g**.

  Note: Supernatants recovered from animal stools such as herbivore may include many particles. To increase the DNA purity, perform an additional centrifugation (1 min @ ≥15,000 g) and transfer the supernatant into a new 2 mL tube before addition of Buffer SF2.

3. Binding

- Transfer the supernatant (~500-600µL) into a 2 mL centrifuge tube (provided).

  Optional: The tube including the pellet may be re-centrifugated for 10 sec @ 15,000 g using a tip to recover any additional lysate.

- Add **600 µL** of **Buffer SF3** to allow binding of the DNA to the Column SF and vortex for 1 sec.

  Optional: a short spin may be performed to recover the mixtures found on the tubing lid and wall using a tip.

- Apply ~**650 uL** of the lysate to the Column SF, **centrifuge for 10 sec** @ **15,000 g** and discard the flow-through. Transfer the remaining mixture to the column.
- Repeat the process until all the lysate has passed through.

4. 1st Wash

- Transfer the Column SF into a new 2 mL collection tube (provided). Add **700 µL** of **Buffer SF4** to the center of the column, **centrifuge for 10 sec** @ **15,000 g**. Discard the flow-through and place the Column back into the same 2 mL Collection Tube

5. 2nd Wash

- Add **700 µL of Buffer SF5** to the center of the Column SF and **centrifuge @ 15,000 g for 1 min**

6. **Drying**

- Transfer Column SF into a new 2mL Collection Tube (provided), **centrifuge @ ≥15,000 g for 2 min.**

7. **Elution**

- Transfer Column SF into a new 1.5mL Collection Tube (provided). Add **35-50 µL of Buffer SF6** to the center of the membrane column, centrifuge @ **≥15,000 g for 10 sec**. Recollect the eluate and reload onto the column to get a maximum concentration or add **35-50 µL of fresh Buffer SF6** to achieve maximum yield. Incubate 5 min and spin for **2 min @ ≥15,000 g**.

  Note: The nucleic acid concentration of the sample is calculated from its UV absorbance at 260 nm where an absorbance of 1 (1 cm path length) is equivalent to 50 µL DNA/mL. Contamination with RNA, protein, salt, ethanol and humic acids or other non-nucleic acid contaminants contributes to the total absorption at 260 nm and therefore leads to an overestimation of the real DNA concentration. When measured using a UV spectroscopy, a ratio of A260/A280 between 1.80-1.90 and A260/A230 >1.8 indicates pure DNA. A260/A280 and A260/A230 ratio above 2.0 indicate RNA contamination. Conversely, an A260/A280 ratio below 1.8 indicates protein contamination. Additionally, a low A260 / A230 ratio indicates the possible presence of humic acids, proteins, saccharides, ethanol, salt, and other contaminants which may inhibit subsequent enzymatic reaction.

7. Flow Chart

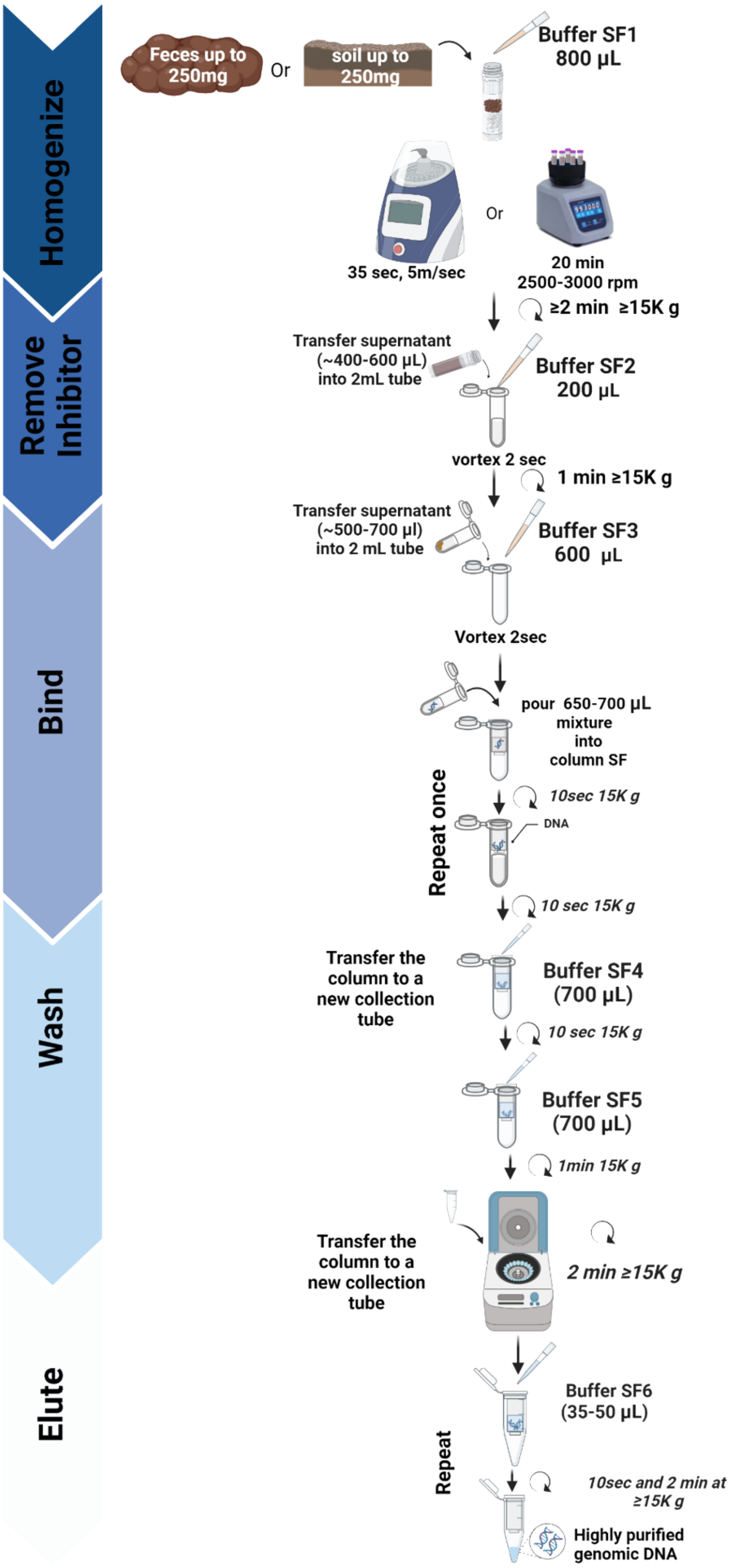

Homogenize
Remove Inhibitor
Bind
Wash
Elute
Feces up to 250mg
Or
soil up to 250mg
Buffer SF1
800 µL
35 sec, 5m/sec
Or
20 min
2500-3000 rpm
≥2 min ≥15K g
Transfer supernatant
(~400-600 µL)
into 2mL tube
Buffer SF2
200 µL
vortex 2 sec
1 min ≥15K g
Transfer supernatant
(~500-700 µl)
into 2 mL tube
Buffer SF3
600 µL
Vortex 2sec
pour 650-700 µL
mixture
into
column SF
Repeat once
10sec 15K g
DNA
10 sec 15K g
Transfer the
column to a
new collection
tube
Buffer SF4
(700 µL)
10 sec 15K g
Buffer SF5
(700 µL)
1min 15K g
Transfer the
column to a
new collection
tube
2 min ≥15K g
Buffer SF6
(35-50 µL)
Repeat
10sec and 2 min at
≥15K g
Highly purified
genomic DNA

8. Data

The SPINeasy DNA Kit for Feces / Soil has been developed and extensively optimized to handle both fecal and soil samples. When used on various feces samples, the SPINeasy DNA Kit for Feces / Soil provided high DNA yields along with optimal A260/A280 and A260/A230 ratio, indicating a high extraction performance across a wide range of sample compositions (Figure 1).

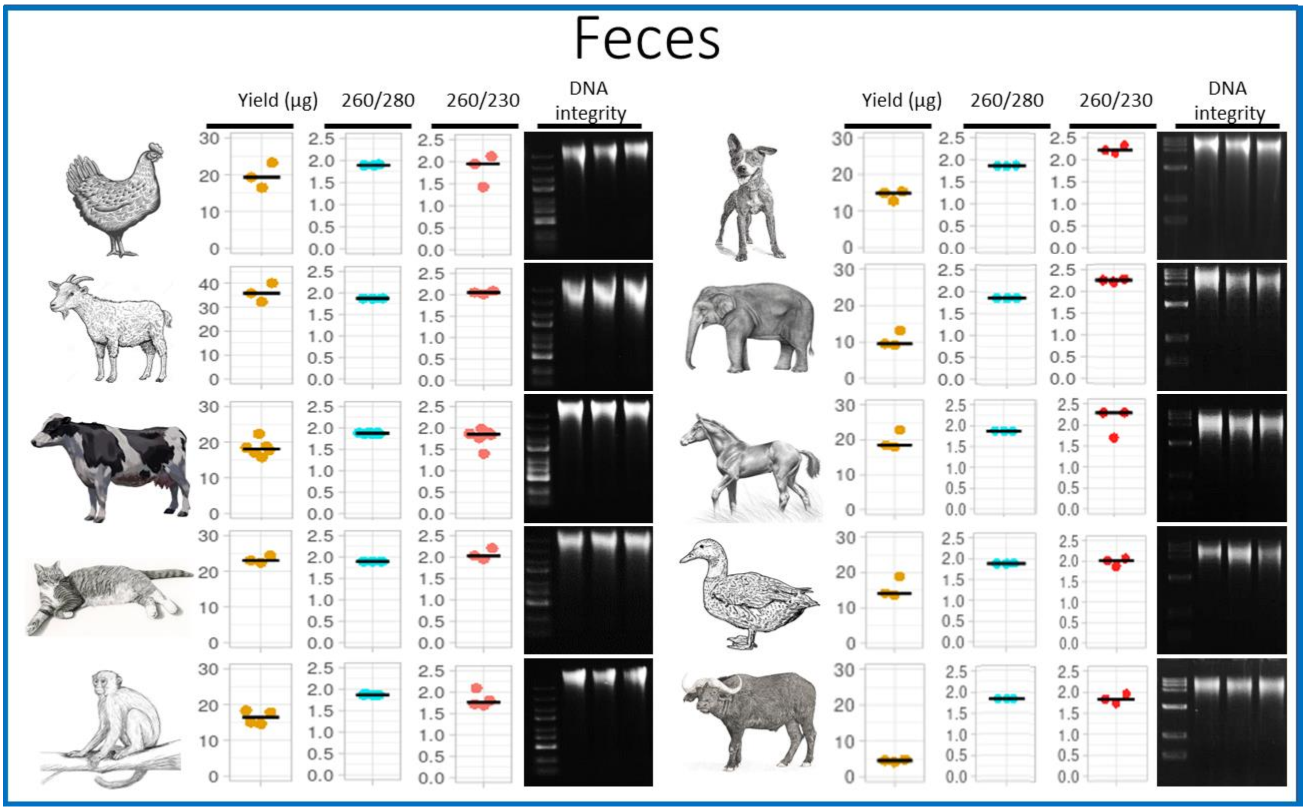

Figure 1. The SPINeasy™ for Feces / Soil kit provides high-performance DNA from various fecal samples

Soil DNA samples prepared using SPINeasy DNA Kit for Feces / Soil also showed optimal yield and purity when compared to a competitor Q pro kit (Figure 2, DNA quality) and can be readily used in long fragment PCR and qPCR without inhibition observed (Figure 2, amplifiability). The unbiased analysis of the microbial community found in samples prepared using various methods revealed that samples obtained using the SPINeasy DNA kit for Feces / Soil included more of difficult to lyse gram+ bacteria such as firmicutes (Figure 2, 16s microbial analyses, left). Moreover, the total number of bacteria identified, and the alpha diversity was higher using SPINeasy DNA kit for feces / Soil samples when compared to all other methods tested, including the competitor Q pro kit (Figure 2, 16s microbial analyses, right).

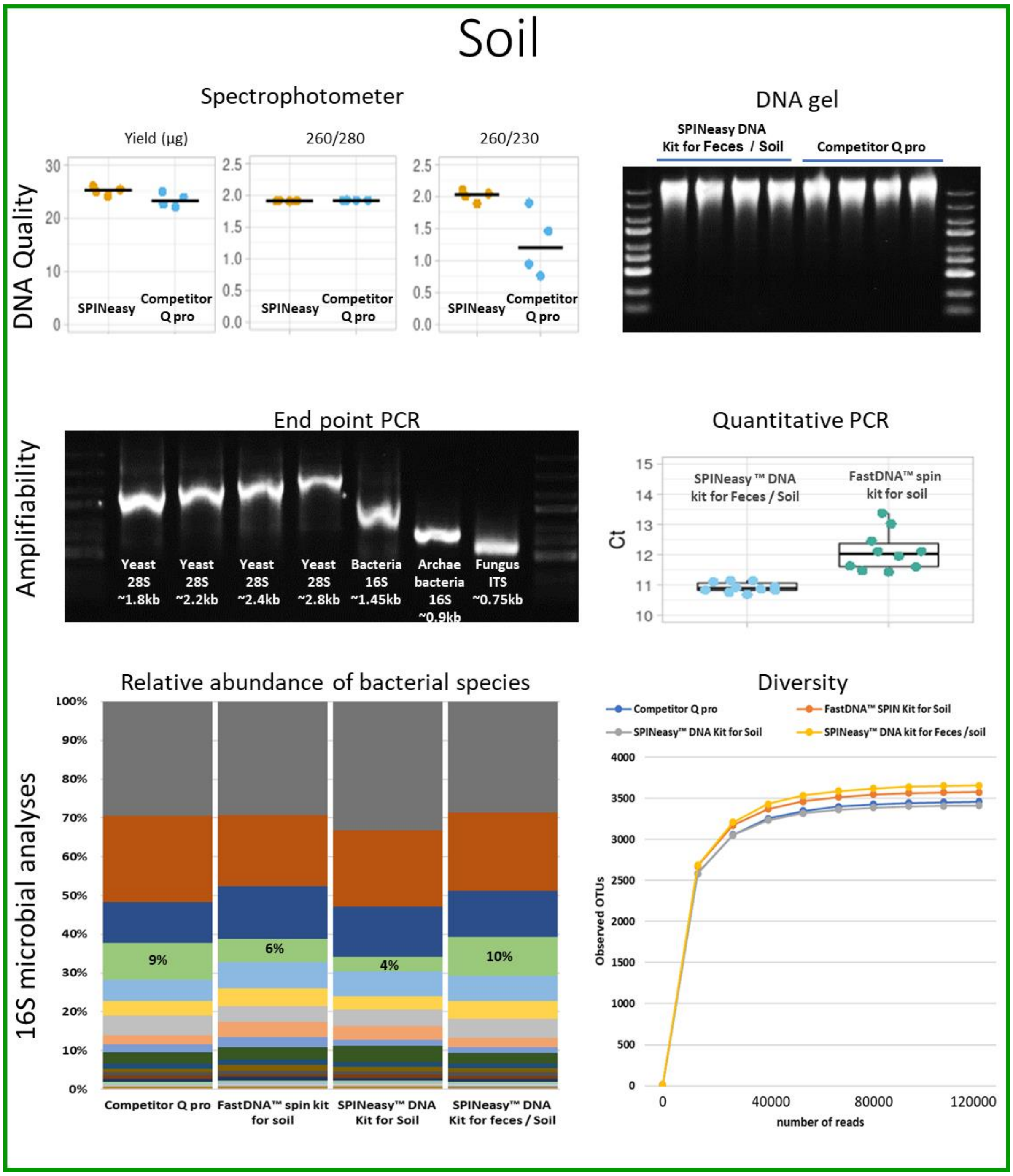

Figure 2. DNA extracted from high biomass / contaminant soil samples using SPINeasy DNA Kit for Feces / Soil and a competitor kit (Q Pro). *DNA quality.* The DNA yield, purity (A260/A280 and A260/A230 ratio) and integrity were assessed using spectrophotometer in quadruplicate and DNA gel, respectively. Each dot of the plot represents a single extraction. The horizontal bars indicate the median value. *Amplifiability.* DNA obtained from soil is more prone to inhibitor contamination. The absence of inhibitor in soil samples obtained using SPINeasy DNA Kit for Feces / Soil kit was confirmed using inhibitor-sensitive PCR and undiluted sample as well as quantitative PCR. *16s microbial analyses.* The hypervariable region V4 of the bacterial 16S rRNA gene was amplified using DNA extracted using the 4 extraction kits described in the legend. Sequences were obtained using a NovaSeq PE250 platform and analyzed using the Qiime 2 pipeline. The relative abundance of bacterial species compilated from 4 technical replicates is shown on the left. The percentage indicates the average proportion of gram positive firmicutes (green) found in DNA samples derived from the same soil sample. The rarefaction curves corresponding to each method are depicted on the right. The alpha diversity was measured by the number of operational taxonomic units (OTUs) identified (vertical axis) following the sequencing depth (horizontal axis).

9. Troubleshooting

Problem	Possible Cause	Recommendation
Wet samples		Remove the beads from the Lysing Matrix YB tubes and transfer into another clean microcentrifuge tube. Add the sample to the empty matrix YB tube and centrifuge for 30 seconds at 15,000 x g. Remove as much liquid as possible with a pipette tip. Add the beads back to the Lysing Matrix YB Tube and resume protocol from step 2.
Low DNA Yield / reduced DNA integrity	Absence of second elution with the same eluate or fresh buffer	An increase of DNA yield by ~20% can be expected by eluting twice.
	Aged sample	Fresh sample is preferred to obtain optimal yield and integrity.
	Elution using water	The elution can be performed using water, but freeze/thaw cycles may degrade DNA. It is recommended to use the elution buffer provided which is compatible with enzymatic reaction or use 5 mM Tris-HCl buffer pH 8.0.
Low A260/A230 or A260/A280 ratios	High level of contaminants in the sample	Perform an additional wash using Buffer SF5 (700 µL).
	Clogged column	Reduce the amount of sample.
	Particles trapped on the membrane	Debris may remain in the supernatant despite the inhibitor removal step and get loaded into the column. Those debris can be removed by inverting the column after drying and tapping the column against the bench.
	Contamination of the column's membrane	Ensure that all traces of wash buffer are removed from the column's rim prior to elution. A kleenwipe ™ or a tip connected to the vacuum manifold may be used.

High A260/A280 or A260/A230 ratios and low molecular weight contaminants observed in DNA gel	Possible RNA contamination	The optimized binding condition employed in the SPINeasy DNA Kit for Feces /Soil enables specific binding of gDNA and thus, the amount of RNA contaminant that is co-purified is low. These low amounts are usually not detectable using canonical detection technique and do not affect most downstream applications. However, a RNase treatment can be performed during the homogenization step by adding 25 µL of RNase A (10 mg/mL) along with Buffer SF1 prior to bead beating homogenization.
Poor PCR Performance	High concentration of DNA	Dilute the DNA sample. Large amount of DNA sample is inhibitory for PCR. Large amount of DNA molecule in the confined space of the reaction vessel is known to lead to false priming, exhaustion of the magnesium ions, primer(s), dNTP(s) and obstruct the passage of the large Taq polymerase molecules. If PCR using undiluted sample is required, check enzyme specification and manufacturer instruction or choose alternative PCR enzyme with strong strand displacement activity. If PCR can be done using diluted sample, the amount of DNA to be used is specific to each PCR enzyme and may need to be optimized by the user. However, genes in multiple copies in the genome such as ribosomal genes require much lesser DNA input. SPINeasy DNA Kit for Feces / Soil allow positive amplifications from various samples using as much as 200 ng or as little as 0.20 ng of DNA per 20 µL of PCR reaction.
	Suboptimal PCR condition.	Verify PCR reagents and protocol with positive control; adjustment on reaction/cycle conditions or primer selection may be necessary following manufacturer recommendation.

10. Product Use Limitation & Warranty

The products presented in this instruction manual are for research or manufacturing use only. They are not to be used as drugs or medical devices in order to diagnose, cure, mitigate, treat or prevent diseases in humans or animals, either as part of an accepted course of therapy or in experimental clinical investigation. These products are not to be used as food, food additives or general household items. Purchase of MP Biomedicals products does not grant rights to reproduce, modify, or repackage the products or any derivative thereof to third parties. MP Biomedicals makes no warranty of any kind, expressed or implied, including merchantability or fitness for any particular purpose, except that the products sold will meet our specifications at the time of delivery.

Buyer’s exclusive remedy and the sole liability of MP Biomedicals hereunder shall be limited to, at our discretion, no replacement or compensation, product credits, refund of the purchase price of, or the replacement of materials that do not meet our specification. By acceptance of the product, Buyer indemnifies and holds MP Biomedicals harmless against, and assumes all liability for, the consequence of its use or misuse by the Buyer, its employees, or others, including, but not limited to, the cost of handling. Said refund or replacement is conditioned on Buyer notifying within thirty (30) days of receipt of product. Failure of Buyer to give said notice within thirty (30) days shall constitute a waiver by the Buyer of all claims hereunder with respect to said material(s).

Australia
Tel: +61 2.8824.2100
Tel: +61 1800.249.998
Email: custserv.au@mpbio.com

Austria & Germany
Tel: 0800.426.67.337
Tel: 00800.7777.9999
Email: custserv.de@mpbio.com

Belgium
Tel: 00800.7777.9999
Email: custserv.be@mpbio.com

Canada
Tel: +1 800.854.0530
Email: custserv.ca@mpbio.com

China
Tel: +86 400.150.0680
Email: custserv.cn@mpbio.com

Europe
Tel: +33 3.88.67.54.25
Tel: +33 00800.7777.9999
Email: custserv.eur@mpbio.com

France
Tel: +33 3.88.67.54.25
Email: custserv.fr@mpbio.com

India
Tel: +91 22.27636921/22/25
Email: custserv.in@mpbio.com

Italy
Tel: 00800.7777.9999
Email: custserv.it@mpbio.com

Japan
Tel: +81 3.6667.0730
Email: custserv.jp@mpbio.com

Latin America
Tel: +1 800.854.0530
Tel: +1 440.337.1200
Email: custserv.la@mpbio.com

New Zealand
Tel: +64 9.912.2460
Email: custserv.nz@mpbio.com

North America
Tel: +1 800.854.0530
Tel: +1 440.337.1200
Email: custserv.na@mpbio.com

Poland
Tel: 00800.7777.9999
Email: custserv.po@mpbio.com

Russia
Tel: +7 495 604.13.44
Email: custserv.rs@mpbio.com

Serbia
Tel: +381 11.242.1972
Email: custserv.se@mpbio.com

Singapore/ APAC
Tel: +65 6775.0008
Tel: +65 6394.7675
Email: custserv.ap@mpbio.com

South Korea
Tel: +82 2.425.5991
Email: custserv.kr@mpbio.com

Switzerland
Tel: 00800.7777.9999
Email: custserv.ch@mpbio.com

The Netherlands
Tel: 00800.7777.9999
Email: custserv.nl@mpbio.com

United Kingdom
Tel: 0800.282.474
Email: custserv.uk@mpbio.com

www.mpbio.com

March 2021

DNeasy® PowerSoil® Pro Kit Handbook

For the isolation of microbial genomic DNA from all soil types, including difficult samples such as compost, sediment, and manure

Sample to Insight

QIAGEN

Contents

Kit Contents 3
Storage 4
Intended Use 4
Safety Information 5
Quality Control 5
Introduction 6
Principle and procedure 6
Automated purification of DNA on the QIAcube instruments 9
Equipment and Reagents to Be Supplied by User 10
Protocol: Experienced User 11
Protocol: Detailed 13
Troubleshooting Guide 17
Ordering Information 19
Document Revision History 22

Kit Contents

DNeasy PowerSoil Pro Kit<br>Catalog no.<br>Number of preps	(50)<br>47014<br>50	(250)<br>47016<br>250
PowerBead Pro Tubes	50	250
MB Spin Columns	50	250
Solution CD1	40 ml	200 ml
Solution CD2	15 ml	60 ml
Solution CD3	35 ml	175 ml
Solution EA	36 ml	175 ml
Solution C5	30 ml	175 ml
Solution C6	9 ml	66 ml
Microcentrifuge Tubes (2 ml)	100	500
Elution Tubes (1.5 ml)	50	250
Collection Tubes (2 ml)	100	500
Quick-Start Protocol	1	1

Storage

Solution CD2 should be stored at 2–8°C upon arrival. All other components and reagents of the DNeasy PowerSoil Pro Kit can be stored at room temperature (15–25°C) until the expiration date printed on the box label.

Intended Use

All DNeasy products are intended for molecular biology applications. These products are not intended for the diagnosis, prevention, or treatment of a disease.

The QIAcube® Connect is designed to perform fully automated purification of nucleic acids and proteins in molecular biology applications. The system is intended for use by professional users trained in molecular biological techniques and the operation of the QIAcube Connect.

All due care and attention should be exercised in the handling of the products. We recommend all users of QIAGEN® products to adhere to the NIH guidelines that have been developed for recombinant DNA experiments, or to other applicable guidelines.

Safety Information

When working with chemicals, always wear a suitable lab coat, disposable gloves, and protective goggles. For more information, please consult the appropriate safety data sheets (SDSs). These are available online in convenient and compact PDF format at **www.qiagen.com/safety** where you can find, view, and print the SDS for each QIAGEN kit and kit component.

**WARNING**	Solution EA and Solution C5 are flammable.

**CAUTION**	DO NOT add bleach or acidic solutions directly to the sample preparation waste.

Solution CD1 and Solution CD3 contain chaotropic salts, which can form highly reactive compounds when combined with bleach. If liquid containing these buffers is spilt, clean with a suitable laboratory detergent and water. If the spilt liquid contains potentially infectious agents, clean the affected area first with laboratory detergent and water, and then with 1% (v/v) sodium hypochlorite.

Quality Control

In accordance with QIAGEN's ISO-certified Quality Management System, each lot of DNeasy PowerSoil Pro Kits is tested against predetermined specifications to ensure consistent product quality.

Introduction

The DNeasy PowerSoil Pro Kit comprises a novel and proprietary method for isolating microbial genomic DNA from environmental samples. The kit uses QIAGEN's second-generation Inhibitor Removal Technology® (IRT) and is intended for use with environmental samples containing high humic acid content, including difficult soil types such as compost, sediment, and manure. Other more common soil and stool types have also been used successfully with this kit. Improved IRT combined with more efficient bead beating and lysis chemistry yields high-quality DNA that can be used immediately in downstream applications, including PCR, qPCR, and next-generation sequencing (16S and whole genome).

Principle and procedure

The DNeasy PowerSoil Pro Kit is effective at removing PCR inhibitors from even the most difficult soil types. Environmental samples are added to a bead-beating tube for rapid and thorough homogenization. Cell lysis occurs by mechanical and chemical methods. Total genomic DNA is captured on a silica membrane in a spin column format. DNA is then washed and eluted from the membrane and ready for NGS, PCR, and other downstream applications.

Bead-beating options

The DNeasy PowerSoil Pro Kit does not require homogenization using a high-velocity bead beater. However, if the microorganism of interest requires stronger homogenization than provided by a vortex, or if using a bead beater is desired, the DNeasy PowerSoil Pro Kit contains bead tubes suitable for high-powered bead beating and may be used in conjunction with the PowerLyzer® 24 Homogenizer (110/220V) (cat. no. 13155) or the TissueLyser II (cat. no. 85300) using a 2 ml Tube Holder Set (cat no. 11993).

The PowerLyzer 24 Homogenizer: Optimized for complete homogenization of any sample

The PowerLyzer 24 Homogenizer is a highly efficient bead-beating system that allows for optimal DNA extraction from a variety of biological samples. The instrument's velocity and proprietary motion combine to provide the fastest homogenization time possible, minimizing the time spent processing the samples. The programmable display enables hands-free, walk-away homogenization with up to 10 cycles of bead beating for as long as 5 minutes per cycle. Even the toughest and most difficult samples, such as pine needles, seeds, spores, and fungal mats are easily and effectively lysed. For more information and protocols, please contact QIAGEN Technical Service at **support.qiagen.com**.

The TissueLyser II: Optimized for medium- to high-throughput sample disruption

The TissueLyser II simultaneously disrupts multiple biological samples through high-speed shaking in plastic tubes with stainless steel, tungsten carbide, or glass beads. Using the appropriate adapter set, up to 48 or 192 samples can be processed at the same time. Alternatively, a grinding jar set can be used to process large samples. A range of beads, bead dispensers, and collection microtubes and caps are also available.

High-throughput options

For high-throughput options, we offer the DNeasy 96 PowerSoil Pro Kit (384) (cat. no. 47017) for processing up to 2 x 96 samples using a centrifuge capable of spinning two stacked 96-well blocks (13 cm x 8 cm x 5.5 cm) at 4500 x *g.* For 96-well homogenization of soil, we offer the TissueLyser II and Plate Adapter Set (cat. no. 85300 and 11990, respectively). We also offer the DNeasy 96 PowerSoil Pro QIAcube HT Kit (5) (cat. no. 47021) and the QIAsymphony PowerFecal® Pro Kit (192) (cat. no. 938036) for automatic processing.

DNeasy PowerSoil Pro Kit Procedure

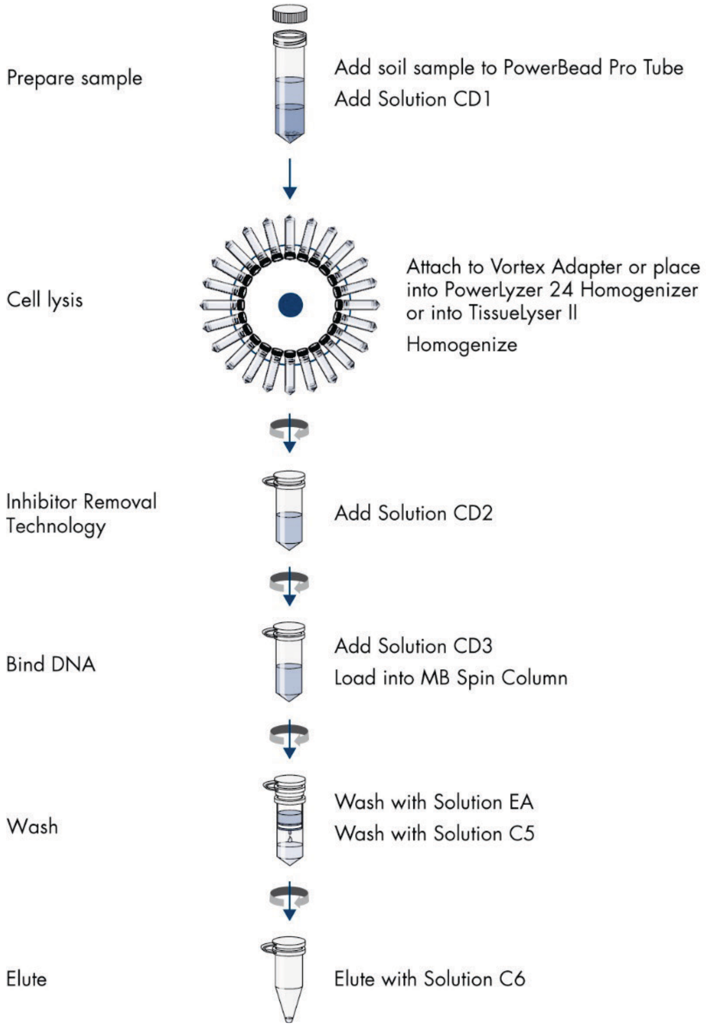

Automated purification of DNA on the QIAcube instruments

Purification of DNA can be fully automated on the QIAcube Connect or the classic QIAcube. The innovative QIAcube instruments use advanced technology to process QIAGEN spin columns, enabling seamless integration of automated, low-throughput sample prep into your laboratory workflow. Sample preparation using the QIAcube instruments follows the same steps as the manual procedure (i.e., lyse, bind, wash, and elute), enabling you to continue using the DNeasy PowerSoil Pro Kit for purification of high-quality DNA.

The QIAcube instruments are preinstalled with protocols for purification of plasmid DNA, genomic DNA, RNA, viral nucleic acids and proteins, plus DNA and RNA cleanup. The range of protocols available is continually expanding, and additional QIAGEN protocols can be downloaded free of charge at **www.qiagen.com/qiacubeprotocols**.

Note: The lyse and remove inhibitors step is not automated in the QIAcube Connect nor in the classic QIAcube and must be performed manually.

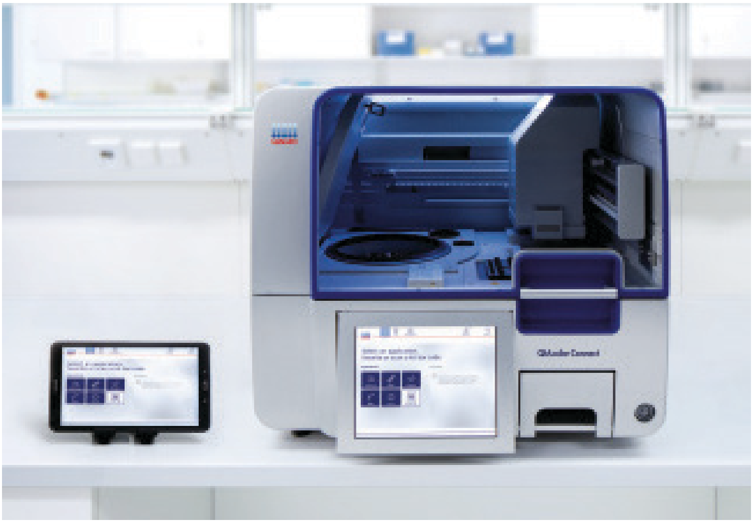

QIAcube Connect.

Equipment and Reagents to Be Supplied by User

When working with chemicals, always wear a suitable lab coat, disposable gloves, and protective goggles. For more information, consult the appropriate safety data sheets (SDSs) available from the product supplier.

- Microcentrifuge (up to16,000 x *g*)
- Pipettor (50–1000 µl)
- Vortex-Genie® 2
- Vortex Adapter for 24 (1.5–2 ml) tubes (cat. no. 13000-V1-24)

Protocol: Experienced User

Important notes before starting

- Ensure that the PowerBead Pro Tubes rotate freely in the centrifuge without rubbing.
- If Solution CD3 has precipitated, heat at 60°C until precipitate dissolves.
- Perform all centrifugation steps at room temperature (15–25°C).

Procedure

1. Spin the PowerBead Pro Tube briefly to ensure that the beads have settled at the bottom. Add up to 250 mg of soil and 800 µl of Solution CD1. Vortex briefly to mix.
2. Secure the PowerBead Pro Tube horizontally on a Vortex Adapter for 1.5–2 ml tubes (cat. no. 13000-V1-24). Vortex at maximum speed for 10 min.

   Note: If using the Vortex Adapter for more than 12 preps simultaneously, increase the vortexing time by 5–10 min.

   Note: For alternative ways to homogenize samples, see the detailed protocol on page 13–14.
3. Centrifuge the PowerBead Pro Tube at 15,000 x *g* for 1 min.
4. Transfer the supernatant to a clean 2 ml Microcentrifuge Tube (provided).

   Note: Expect 500–600 µl. The supernatant may still contain some soil particles.
5. Add 200 µl of Solution CD2 and vortex for 5 s.
6. Centrifuge at 15,000 x *g* for 1 min. Avoiding the pellet, transfer up to 700 µl of supernatant to a clean 2 ml Microcentrifuge Tube (provided).

   Note: Expect 500–600 µl.
7. Add 600 µl of Solution CD3 and vortex for 5 s.
8. Load 650 µl of lysate to an MB Spin Column. Centrifuge at 15,000 x *g* for 1 min.
9. Discard the flow-through and repeat step 8 to ensure that all of the lysate has passed through the MB Spin Column.

10. Carefully place the MB Spin Column into a clean 2 ml Collection Tube (provided). Avoid splashing any flow-through onto the MB Spin Column.
11. Add 500 µl of Solution EA to the MB Spin Column. Centrifuge at 15,000 x *g* for 1 min.
12. Discard the flow-through and place the MB Spin Column back into the same 2 ml Collection Tube.
13. Add 500 µl of Solution C5 to the MB Spin Column. Centrifuge at 15,000 x *g* for 1 min.
14. Discard the flow-through and place the MB Spin Column into a new 2 ml Collection Tube (provided).
15. Centrifuge at up to 16,000 x *g* for 2 min. Carefully place the MB Spin Column into a new 1.5 ml Elution Tube (provided).
16. Add 50–100 µl of Solution C6 to the center of the white filter membrane.
17. Centrifuge at 15,000 x *g* for 1 min. Discard the MB Spin Column. The DNA is now ready for downstream applications.

    Note: We recommend storing the DNA frozen (–30 to –15°C or –90 to –65°C) as Solution C6 does not contain EDTA. To concentrate DNA, refer to the Troubleshooting Guide.

Protocol: Detailed

Important notes before starting

- Ensure that the PowerBead Pro Tubes rotate freely in the centrifuge without rubbing.
- If Solution CD3 has precipitated, heat at 60°C until precipitate dissolves.
- Perform all centrifugation steps at room temperature (15–25°C).

Procedure

1. Spin the PowerBead Pro Tube briefly to ensure that the beads have settled at the bottom. Add up to 250 mg of soil and 800 µl of Solution CD1. Vortex briefly to mix.

   Note: After the sample has been loaded into the PowerBead Pro Tube, the next step is a homogenization and lysis procedure. The PowerBead Pro Tube contains a buffer that will (a) help disperse the soil particles, (b) begin to dissolve humic acids, and (c) protect nucleic acids from degradation. Gentle vortexing mixes the components in the PowerBead Pro Tube and begins to disperse the sample in the buffer.

2. Homogenize samples thoroughly using one of the following methods:

   2a. Secure the PowerBead Pro Tube horizontally on a Vortex Adapter for 1.5–2 ml tubes (cat. no. 13000-V1-24). Vortex at maximum speed for 10 min.
   Note: If using Vortex Adapter for more than 12 preps simultaneously, increase the vortexing time by 5–10 min.

   Note: Using the Vortex Adapter will maximize homogenization, which can lead to higher DNA yields. Avoid using tape, which can become loose and result in reduced homogenization efficiency, inconsistent results, and reduced yields.

2b. Use a PowerLyzer 24 Homogenizer. PowerBead Pro Tubes must be properly balanced in the tube holder of the PowerLyzer 24 Homogenizer. We recommend homogenizing the soil at 2000 rpm for 30 s, pausing for 30 s, then homogenizing again at 2000 rpm for 30 s.

Note: Homogenizing samples at higher speeds (up to 4000 rpm) may increase yields but result in more fragmented DNA.

2c. Use a TissueLyser II. Place the PowerBead Pro Tube into the TissueLyser Adapter Set 2 x 24 (cat. no. 69982) or 2 ml Tube Holder (cat. no. 11993) and Plate Adapter Set (cat. no. 11990). Fasten the adapter into the instrument and shake for 5 min at speed 25 Hz. Reorient the adapter so that the side that was closest to the machine body is now furthest from it. Shake again for 5 min at a speed of 25 Hz.

Note: Vortexing/shaking is critical for complete homogenization and cell lysis. Cells are lysed by a combination of chemical agents from step 1 and mechanical shaking introduced at this step. Randomly shaking the beads in the presence of disruption agents will cause the beads to collide with microbial cells and lead to the cells breaking open.

3. Centrifuge the PowerBead Pro Tube at 15,000 x *g* for 1 min.
4. Transfer the supernatant to a clean 2 ml Microcentrifuge Tube (provided).

   Note: Expect 500–600 µl. The supernatant may still contain some soil particles.
5. Add 200 µl of Solution CD2 and vortex for 5 s.

   Note: Solution CD2 contains IRT, which is a reagent that can precipitate non-DNA organic and inorganic material including humic substances, cell debris, and proteins. It is important to remove contaminating organic and inorganic matter that may reduce DNA purity and inhibit downstream DNA applications.

6. Centrifuge at 15,000 x *g* for 1 min. Avoiding the pellet, transfer up to 700 µl of supernatant to a clean 2 ml Microcentrifuge Tube (provided).

   Note: Expect 500–600 µl.

   Note: The pellet at this point contains non-DNA organic and inorganic material including humic acids, cell debris, and proteins. For best DNA yields and quality, avoid transferring any of the pellet.

7. Add 600 µl of Solution CD3 and vortex for 5 s.

   Note: Solution CD3 is a high-concentration salt solution. Because DNA binds tightly to silica at high salt concentrations, Solution CD3 will adjust the DNA solution salt concentration to allow binding of DNA, but not non-DNA organic and inorganic material that may still be present at low levels, to the MB Spin Column filter membrane.

8. Load 650 µl of the lysate onto an MB Spin Column and centrifuge at 15,000 x *g* for 1 min.

   Note: DNA is selectively bound to the silica membrane in the MB Spin Column in the presence of high salt solution. Contaminants pass through the filter membrane, leaving only DNA bound to the membrane.

9. Discard the flow-through and repeat step 8 to ensure that all of the lysate has passed through the MB Spin Column.

10. Carefully place the MB Spin Column into a clean 2 ml Collection Tube (provided). Avoid splashing any flow-through onto the MB Spin Column.

11. Add 500 µl of Solution EA to the MB Spin Column. Centrifuge at 15,000 x *g* for 1 min.

    Note: Solution EA is a wash buffer that removes protein and other non-aqueous contaminants from the MB Spin Column filter membrane.

12. Discard the flow-through and place the MB Spin Column back into the same 2 ml Collection Tube.

13. Add 500 µl of Solution C5 to the MB Spin Column. Centrifuge at 15,000 x *g* for 1 min.

    Note: Solution C5 is an ethanol-based wash solution used to further clean the DNA that is bound to the silica filter membrane in the MB Spin Column. This wash solution removes residual salt, humic acid, and other contaminants while allowing the DNA to stay bound to the silica membrane.

14. Discard the flow-through and place the MB Spin Column into a new 2 ml Collection Tube (provided).

15. Centrifuge at up to 16,000 x *g* for 2 min. Carefully place the MB Spin Column into a new 1.5 ml Elution Tube (provided).

    Note: This spin removes residual Solution C5. It is critical to remove all traces of Solution C5 because the ethanol in it can interfere with downstream DNA applications, such as PCR, restriction digests, and gel electrophoresis.

16. Add 50–100 µl of Solution C6 to the center of the white filter membrane.

    Note: Placing Solution C6 in the center of the small white membrane will make sure the entire membrane is wet. This will result in a more efficient and complete release of the DNA from the MB Spin Column filter membrane. As Solution C6 passes through the silica membrane, DNA that was bound in the presence of high salt is selectively released by Solution C6 (10 mM Tris), which lacks salt.

17. Centrifuge at 15,000 x *g* for 1 min. Discard the MB Spin Column. The DNA is now ready for downstream applications.

    Note: We recommend storing the DNA frozen (–30 to –15°C or –90 to –65°C) as Solution C6 does not contain EDTA. To concentrate DNA, refer to the Troubleshooting Guide.

Troubleshooting Guide

This troubleshooting guide may be helpful in solving any problems that may arise. For more information, see also the Frequently Asked Questions page at our Technical Support Center: **www.qiagen.com/FAQ/FAQList.aspx**. The scientists in QIAGEN Technical Services are always happy to answer any questions you may have about either the information and/or protocols in this handbook or sample and assay technologies. For contact information, visit **www.qiagen.com**.

		Comments and suggestions
**Soil Processing**		
a)	Amount of soil to process	The QIAGEN DNeasy PowerSoil Pro Kit is designed to process 0.25 g of soil. For inquiries regarding the use of larger sample amounts, please contact Technical Support for suggestions.
b)	Soil sample is high in water content	Remove contents from the PowerBead Pro Tube (beads) and transfer into another sterile microcentrifuge tube (not provided). Add soil sample to PowerBead Pro Tube and centrifuge at room temperature (15–25°C) for 30 s at 10,000 x *g*. Remove as much liquid as possible with a pipette tip. Add beads back to PowerBead Pro Tube and resume protocol from step 2.
**DNA**		
a)	DNA does not amplify	Ensure that you check DNA yields by gel electrophoresis or spectrophotometer reading. An excess amount of DNA will inhibit a PCR reaction.<br>Diluting the template DNA should not be necessary with DNA isolated using the DNeasy PowerSoil Pro DNA Kit. However, it should still be attempted.<br>If DNA will still not amplify after trying the steps above, then PCR optimization may be needed.
b)	Eluted DNA is brown	If you observe coloration in your samples, please contact Technical Support for suggestions.

		Comments and suggestions
c)	Concentrating eluted DNA	The final volume of eluted DNA will be 50–100 µl. The DNA may be concentrated by adding 5–10 µl of 3 M NaCl and inverting 3–5 times to mix. Next, add 100 µl of 100% cold ethanol and invert 3–5 times to mix. Incubate at –30 to –15°C for 30 min and centrifuge at 10,000 x *g* for 5 min at room temperature. Decant all liquid. Briefly dry residual ethanol in a speed vac or ambient air. Avoid over-drying the pellet or resuspension may be difficult. Resuspend precipitated DNA in desired volume of 10 mM Tris (Solution C6).
d)	DNA floats out of a well when loading a gel	This usually occurs because residual Solution C5 remains in the final sample. Prevent this by being careful in step 15 and not transferring liquid onto the bottom of the spin filter basket. Ethanol precipitation (described in "Concentrating eluted DNA") is the best way to remove residual Solution C5.
e)	Storing DNA	DNA is eluted in Solution C6 (10 mM Tris) and must be stored at −30 to −15 or −90 to −65°C to prevent degradation. DNA can be eluted in TE without loss, but the EDTA may inhibit downstream reactions such as PCR and automated sequencing. DNA may also be eluted in sterile, DNA-free PCR-grade water (cat. no. 17000-10).
**Alternative lysis methods**		
a)	Cells are difficult to lyse	After adding Solution CD1 and prior to the bead-beating step, incubate at 65°C for 10 min. Resume protocol from step 2.
b)	Reduction of shearing of DNA	After adding Solution CD1, vortex 3–4 seconds, then heat to 70°C for 5 min. Repeat once. This alternative procedure will reduce shearing but may also reduce yield.

Ordering Information

Product	Contents	Cat. no.
DNeasy PowerSoil Pro Kit (50)	For 50 preps: Isolate microbial genomic DNA from all soil types	47014
DNeasy PowerSoil Pro Kit (250)	For 250 preps: Isolate microbial genomic DNA from all soil types	47016
**QIAcube Connect — for fully automated nucleic acid extraction with QIAGEN spin-column kits**		
QIAcube Connect*	Instrument, connectivity package, 1-year warranty on parts and labor	9002864
Starter Pack, QIAcube	Filter-tips, 200 µl (1024), 1000 µl filter-tips (1024), 30 ml reagent bottles (12), rotor adapters (240), elution tubes (240), rotor adapter holder	990395
**Related products**		
DNeasy 96 PowerSoil Pro Kit (384)	For 384 preps: Isolate microbial genomic DNA from all soil types in a 96-well format	47017
DNeasy 96 PowerSoil Pro QIAcube HT Kit (480)	For 480 prep: High-throughput isolation of microbial genomic DNA from all soil types, optimized for QIAcube HT	47021
QIAsymphony PowerFecal Pro DNA Kit (192)	For the isolation of microbial genomic DNA from stool and soil on the QIAsymphony	938036

Product	Contents	Cat. no.
PowerBead Pro Tubes (2 ml) (50)	Bead tubes ready for rapid and reliable biological sample lysis from a wide variety of starting materials, 2 ml	19301
DNeasy PowerMax® Soil Kit (10)	For 10 preps: Isolate microbial DNA from large quantities of soil; great for samples with low microbial load	12988-10
MagAttract® PowerSoil Pro DNA Kit (384)	For 384 preps: Hands-free isolation of DNA from soil and stool using automated processing and liquid-handling systems	47109
Vortex Adapter	For vortexing 1.5 ml or 2 ml tubes using the Vortex-Genie 2	13000-V1-24
PowerLyzer 24 Homogenizer (110/220V)	For complete lysis and homogenization of any biological sample	13155
TissueLyser II	For medium- to high-throughput sample disruption for molecular analysis	85300
TissueLyser Adapter Set (2 x 24)	2 sets of adapter plates and 2 racks for use with 2 ml microcentrifuge tubes on the TissueLyser II	69982
2 ml Tube Holder Set	For sample homogenization in 2 ml bead tubes on a TissueLyser II	11993

Product	Contents	Cat. no.
UCP Multiplex PCR Kit (100)	For 100 reactions: For highly specific and sensitive multiplex PCR with minimized background using nucleic acid-depleted reagents	206742
UCP Multiplex PCR Kit (500)	For 500 reactions: For highly specific and sensitive multiplex PCR with minimized background using nucleic acid-depleted reagents	206744

* All QIAcube Connect instruments are provided with a region-specific connectivity package, including tablet and equipment necessary to connect to the local network. Further, QIAGEN offers comprehensive instrument service products, including service agreements, installation, introductory training, and preventive subscription. Contact your local sales representative to learn about your options.

For up-to-date licensing information and product-specific disclaimers, see the respective QIAGEN kit handbook or user manual. QIAGEN kit handbooks and user manuals are available at **www.qiagen.com** or can be requested from QIAGEN Technical Services or your local distributor.

Document Revision History

Date	Changes
05/2019	Added storage condition for Solution CD2 in the Storage section; Deleted Figure 1 QIAcube instrument; Added QIAcube Connect as reference in the Principle and Procedure topic; Updated Protocol: Experience User and Protocol: Detailed sections to provide information on bead-beating methods. Revised Ordering Information section to add DNeasy 96 PowerSoil Pro QIAcube HT Kit (480), replace DNeasy PowerSoil HTP 96 Kit with DNeasy 96 PowerSoil Pro Kit, and change QIAcube (110V) to QIAcube Connect; Layout updates
01/2020	Updated storage information, high-throughput options, homogenization steps in protocol and cross-references to homogenization sections. Removed statement about sterility of Solution C6. Updated text, ordering information and intended use for QIAcube Connect.
03/2021	Changed “tissue” sample to “soil” sample and in section “Protocol: Detailed”, procedure item 2b. Corrected the catalog number of Plate Adapter Set in section “Protocol: Detailed”, procedure item 2c. Included the catalog number of the QIAcube Connect; updated the product name of PowerLyzer 24 Homogenizer (110/220V); updated the product name, description, and cat. no. of MagAttract PowerSoil Pro DNA Kit (384); and removed RNeasy PowerSoil Total RNA Kit in the Ordering Information section.

Limited License Agreement for DNeasy PowerSoil Pro Kit

Use of this product signifies the agreement of any purchaser or user of the product to the following terms:

1. The product may be used solely in accordance with the protocols provided with the product and this handbook and for use with components contained in the kit only. QIAGEN grants no license under any of its intellectual property to use or incorporate the enclosed components of this kit with any components not included within this kit except as described in the protocols provided with the product, this handbook, and additional protocols available at www.qiagen.com. Some of these additional protocols have been provided by QIAGEN users for QIAGEN users. These protocols have not been thoroughly tested or optimized by QIAGEN. QIAGEN neither guarantees them nor warrants that they do not infringe the rights of third-parties.
2. Other than expressly stated licenses, QIAGEN makes no warranty that this kit and/or its use(s) do not infringe the rights of third-parties.
3. This kit and its components are licensed for one-time use and may not be reused, refurbished, or resold.
4. QIAGEN specifically disclaims any other licenses, expressed or implied other than those expressly stated.
5. The purchaser and user of the kit agree not to take or permit anyone else to take any steps that could lead to or facilitate any acts prohibited above. QIAGEN may enforce the prohibitions of this Limited License Agreement in any Court, and shall recover all its investigative and Court costs, including attorney fees, in any action to enforce this Limited License Agreement or any of its intellectual property rights relating to the kit and/or its components.

For updated license terms, see **www.qiagen.com**.

Trademarks: QIAGEN®, Sample to Insight®, QIAcube®, DNeasy®, Inhibitor Removal Technology®, MagAttract®, PowerFecal®, PowerLyzer®, PowerMax®, PowerSoil®, RNeasy® (QIAGEN Group); Vortex-Genie® (Scientific Industries). Registered names, trademarks, etc. used in this document, even when not specifically marked as such, are not to be considered unprotected by law.

03/2021 HB-2495-005 © 2021 QIAGEN, all rights reserved.

ZYMO RESEARCH

DNA Purification Made Simple

Quick-DNA™ Fecal/Soil Microbe Miniprep Kit

DNA from fecal, soil, and microbial samples.

Highlights

- Rapid method for the isolation of inhibitor-free, PCR-quality DNA (up to 25 µg/prep) from microbes including Gram-positive and Gram-negative bacteria, fungi, algae, protozoa, etc. in fecal and soil samples in as little as 20 minutes.
- State-of-the-art, ultra-high density **BashingBeads™** are fracture resistant and chemically inert.
- Omits the use of organic denaturants as well as proteinases.

Catalog Numbers:
D6010

Scan with your smart-phone camera to view the online protocol/video.

tech@zymoresearch.com www.zymoresearch.com Toll Free: (888) 882-9682

Table of Contents

Product Contents 01
Specifications 02
Product Description....................................... 03
Protocol 06
Appendix... 08
Ordering Information 09
Guarantee 11

Product Contents

Quick-DNA™ Fecal/Soil Microbe Miniprep Kit	**D6010** (50 Preps)	**Storage Temperature**
ZR BashingBead™ Lysis Tubes (0.1 & 0.5 mm)	50	Room Temp.
BashingBead™ Buffer	40 ml	Room Temp.
Genomic Lysis Buffer[1]	100 ml	Room Temp.
DNA Pre-Wash Buffer[2]	15 ml	Room Temp.
g-DNA Wash Buffer	50 ml	Room Temp.
DNA Elution Buffer	10 ml	Room Temp.
Prep Solution	30 ml	Room Temp.
Zymo-Spin™ III-F Filters	50	Room Temp.
Zymo-Spin™ III-HRC Filters	50	Room Temp.
Zymo-Spin™ IICR Columns	50	Room Temp.
Collection Tubes	200	Room Temp.
Instruction Manual	1	-

1 For optimal performance, add beta-mercaptoethanol to 0.5% (v/v) *i.e.*, 500 µl per 100 ml.
2 A precipitate may have formed in the **DNA Pre-Wash Buffer** during shipping. To completely resuspend the buffer, incubate the bottle at 30–37ºC for 30 minutes and mix by inversion. DO NOT MICROWAVE.

Specifications

- **Format –** Bead Beating, Spin Column.
- **Sample Sources –** Host, bacterial, fungal, algal, protozoan, viral DNA can be isolated from up to 150 mg of feces or up to 250 mg of soil. The amount of soil sample processed will vary depending on the composition of the sample: process more soil material for wet muddy samples and less for dry sandy samples. Additionally, water[1] or 50 – 100 mg (wet weight) fungal/bacterial cells[2] can be isolated.
- **DNA Purity –** High quality, inhibitor-free DNA is eluted with DNA Elution Buffer suitable for the amplification of bacterial, protist, and/or mammalian templates (A_{260}/A_{280} >1.8)
- **DNA Size Limits –** Capable of recovering genomic DNA up to and above 40 kb. In most instances, mitochondrial DNA and viral DNA (if present) will also be recovered.
- **DNA Recovery –** Typically, up to 25 µg total DNA is eluted into 100 µl (50 µl minimum) **DNA Elution Buffer** per sample.
- **Equipment –** – Microcentrifuge, vortex, cell disrupter/disrupter (recommended).

---

1 For water samples, use desired filter to collect sample (not provided) and cut the filter into small pieces before adding to the lysis tube.
2 This equates to approximately 10^9 bacterial cells and 10^8 yeast cells.

Product Description

The ***Quick*-DNA™ Fecal/Soil Microbe Miniprep Kit** is designed for the simple, rapid isolation of inhibitor-free, PCR-quality DNA from a variety of fecal (including humans, birds, rats, mice, cattle, etc.) and soil (including clay, sandy, silty, peaty, chalky, and loamy soils) samples. The kit can be used to successfully isolate DNA from tough-to-lyse Gram-positive and Gram-negative bacteria, fungi, algae, protozoa, etc. that inhabit fecal and soil samples. The procedure is easy and can be completed in as little as 15 minutes: fecal samples (≤150 mg each) or soil samples (≤250 mg each) are added directly to a **ZR BashingBead™ Lysis Tube (0.1 & 0.5 mm)** and rapidly and efficiently lysed by bead beating without the use of organic denaturants or proteinases. Zymo-Spin™ Technology is then used to isolate the DNA, which is subsequently filtered to remove humic acids/polyphenols that inhibit PCR. The DNA is ideal for downstream molecular-based applications including PCR, arrays, genotyping, etc. A schematic of the ***Quick*-DNA™ Fecal/Soil Microbe Miniprep Kit** procedure is shown below.

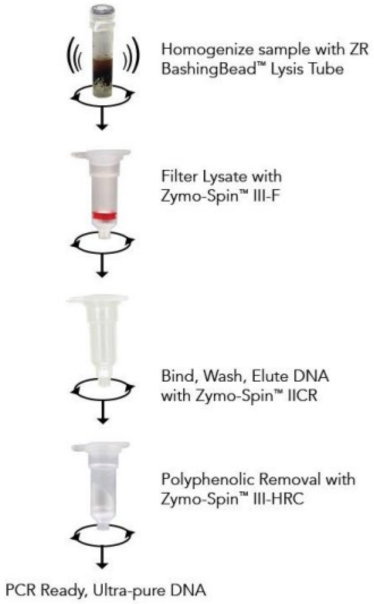

DNA/RNA Shield™ (R1100-50, R1100-250) can be used to stabilize nucleic acids and inactivate infectious agents in a variety of samples, without the need for reagent removal.

For rapid, robust, and simple purification of high quality, inhibitor-free DNA from any sample including feces, soil, water, biofilms, swabs, saliva, body fluids, etc. use the **ZymoBIOMICS™ DNA Miniprep Kit (D4300)**.

Fecal DNA Isolation

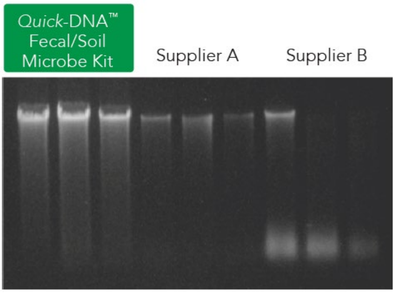

Comparison of DNA yields from rat feces using the ***Quick*-DNA™ Fecal/Soil Microbe Kit** and kits from suppliers A and B. Equivalent amounts of feces were processed using each kit and then equal volumes of eluted DNA were analyzed in a 0.8% (w/v) agarose/ethidium bromide gel. Samples were processed in triplicate.

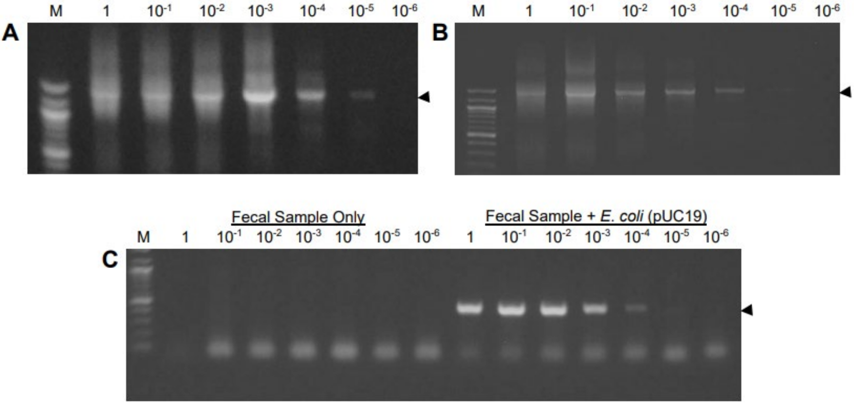

PCR of DNAs from rat and human fecal samples isolated with the ***Quick*-DNA™ Fecal/Soil Microbe Kit**. Panels A and B show the results of PCR with DNA isolated from rat and human fecal samples, respectively, using primers specific for prokaryotic 16S rRNA. Panel C shows the results of PCR of DNA isolated from human feces with and without the addition of *E. coli* containing pUC19 plasmid DNA (indicated at the top of the image) using primers specific for the pUC19 sequence. In each case, amplicons were analyzed in a 1.5% (w/v) agarose / ethidium bromide gel using a UV imager. Numbers above each lane of the gel images are the volumetric equivalent (in µl) of eluted DNA (100 µl) used for PCR. Arrows mark the relative migration of amplicons in the gels, and M is a 100 bp DNA ladder (NEB).

Soil Microbe DNA Isolation

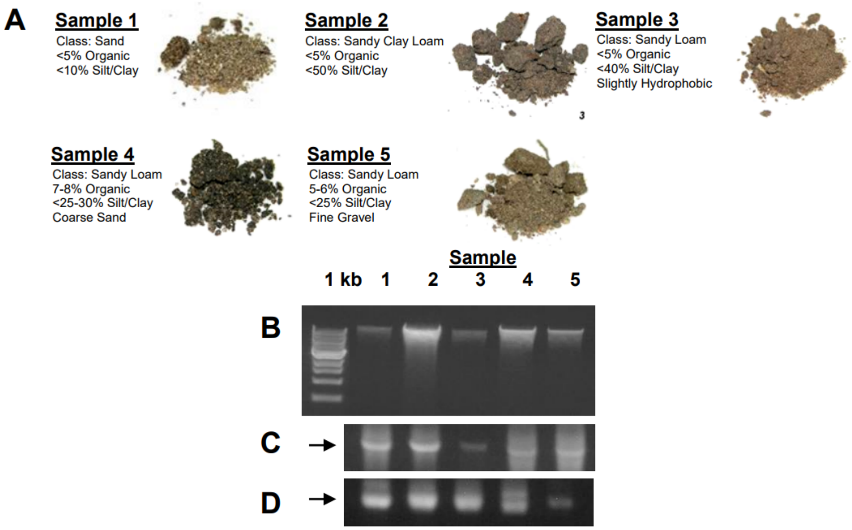

The ***Quick*-DNA™ Fecal/Soil Microbe Kit** can be used to isolate high quality DNA from a variety of soil types which yields robust products following PCR. **Panel A**: Physical characteristics of sampled soils (1-5) (Ref. 1). **Panel B**: Microbial DNA was isolated from soil samples (1-5) using the ***Quick*-DNA™ Fecal/Soil Microbe Kit**. Approximately 10% of the eluted DNA was then separated in a 0.8% (w/v) agarose/ethidium bromide gel. **Panels C** and **D** show the results of PCR of microbial DNA isolated from the samples with primers specific for prokaryotic 16S rRNA (**C**) or eukaryotic rRNA (**D**). In the figures, the 1 kb size marker (NEB) is as indicated and the arrows show the prokaryotic 16S rRNA and eukaryotic rRNA PCR products.

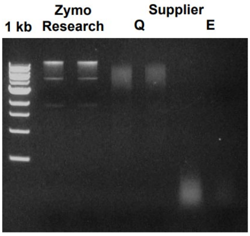

DNA isolated from *Saccharomyces cerevisiae* (strain TMY18) using the ***Quick*-DNA™ Fecal/Soil Microbe Kit** is high-quality and structurally intact. Equivalent amounts of yeast were processed using the ***Quick*-DNA™ Fecal/Soil Microbe Kit** or the kits from suppliers Q and E. Equal volumes of eluted DNA were then analyzed in a 0.8% (w/v) agarose/ethidium bromide gel. The size marker is a 1 kb ladder (NEB).

References:
1. Soil and Plant Laboratory, Inc. P.O. Box 11744, Santa Ana, California 92711

Protocol

For optimal performance, add beta-mercaptoethanol (user supplied) to the **Genomic Lysis Buffer** to a final dilution of 0.5% (v/v) *i.e.*, 500 µl per 100 ml.

1. Add ≤ 150 mg of fecal sample or ≤ 250 mg of soil sample to a **ZR BashingBead™ Lysis Tube (0.1 & 0.5 mm)**. Add 750 µl **BashingBead™ Buffer** to the tube[1].

   Note: *Alternatively, add water sample[2] or 50-100 mg (wet weight) fungal/bacterial cells[3] that have been resuspended in up to 200 µl of water or isotonic buffer (e.g., PBS) to a* ***ZR BashingBead™ Lysis Tube****.*

   Note: *For samples stored in DNA/RNA Shield™, add up to 1 ml to a ZR BashingBead™ Lysis Tube. Do not add BashingBead™ Buffer and proceed to Step 2.*

2. Secure in a bead beater fitted with a 2 ml tube holder assembly and process using optimized beat beating conditions (speed and time) for your device (see Appendix).

3. Centrifuge the **ZR BashingBead™ Lysis Tube (0.1 & 0.5 mm**) in a microcentrifuge at ≥ 10,000 x *g* for 1 minute.

4. Transfer up to 400 µl supernatant to a **Zymo-Spin™ III-F Filter** in a **Collection Tube** and centrifuge at 8,000 x *g* for 1 minute.

5. Add 1,200 µl of **Genomic Lysis Buffer** to the filtrate in the **Collection Tube** from Step 4. Mix well.

6. Transfer 800 µl of the mixture from Step 5 to a **Zymo-Spin™ IICR Column**[4] in a **Collection Tube** and centrifuge at 10,000 x *g* for 1 minute.

7. Discard the flow through from the **Collection Tube** and repeat Step 6.

---

1 Cap tube tightly to prevent leakage.
2 For water samples, filter using desired non-silica based filter (not provided). Cut the filter into small pieces before adding to the lysis tube.
3 This equates to approximately 10^9 bacterial cells and 10^8 yeast cells.
4 The **Zymo-Spin™ IICR Column** has a maximum capacity of 800 µl.

8. Add 200 µl **DNA Pre-Wash Buffer** to the **Zymo-Spin™ IICR Column** in a new **Collection Tube** and centrifuge at 10,000 x *g* for 1 minute.

9. Add 500 µl **g-DNA Wash Buffer** to the **Zymo-Spin™ IICR Column** and centrifuge at 10,000 x *g* for 1 minute.

10. Transfer the **Zymo-Spin™ IICR Column** to a clean 1.5 ml microcentrifuge tube and add 100 µl (50 µl minimum) **DNA Elution Buffer** directly to the column matrix. Centrifuge at 10,000 x *g* for 30 seconds to elute the DNA[5, 6].

11. Place a **Zymo-Spin™ III-HRC Filter** in a clean **Collection Tube** and add 600 µl **Prep Solution**. Centrifuge at 8,000 x *g* for 3 minutes.

12. Transfer the eluted DNA to a prepared **Zymo-Spin™ III-HRC Filter** in a clean 1.5 ml microcentrifuge tube and centrifuge at exactly 16,000 x *g* for 3 minutes.

    The filtered DNA is now suitable for PCR and other downstream applications.

---

5 In some cases a brown-colored pellet may form at the bottom of the tube after centrifugation. Avoid this pellet when collecting the eluted DNA.

6 If fungi or bacterial cultures were sampled, the DNA is now suitable for PCR as well as other downstream applications.

Appendix

Optimized Lysis Protocols for Bead-Beating

The following conditions with different mechanical lysis machines were validated with minimum bias using the **ZymoBIOMICS™ Microbial Community Standard**.

1 — Vortex Genie® with 2 ml BashingBead™ Tubes

Recommended for ease of use and accessibility

Use Microtube Adaptor (Scientific Industries, Inc. Cat. No. S5001-7)

1. 40 minutes of continuous bead beating (max of 18 tubes per adaptor)

2 — Bertin Precellys® Evolution with 2 ml BashingBead™ Tubes

Recommended for ease of use and ultra-high speed

1. 1 minute on at 9,000 rpm
2. 2 minutes rest
3. Repeat cycle 4 times for a total of 4 minutes of bead beating

3 — MP Fastprep®-24 with 2 ml BashingBead™ Tubes

Maximum of 20 tubes. The weight of >20 tubes may cause a system error

1. 1 minute on at max speed
2. 5 minutes rest
3. Repeat cycle 5 times for a total of 5 minutes of bead beating

4 — Omni Bead Ruptor® Elite with 2 ml BashingBead™ Tubes

1. 1 minute on at 6 m/s
2. 5 minutes rest
3. Repeat cycle 3 times for a total of 3 minutes of bead beating

5 — Biospec Mini-BeadBeater-16 with 2 ml BashingBead™ Tubes

1. 1 minute at maximum speed
2. 5 minutes rest
3. Repeat cycle 5 times for a total of 5 minutes of bead beating

6 — Biospec Mini-BeadBeater-96 with 2 ml BashingBead™ Tubes

1. 5 minutes on at Max RPM
2. 5 minutes rest
3. Repeat cycle 4 times for a total of 20 minutes of bead beating

7 — Biospec Mini-BeadBeater-96 with 96 well BashingBead™ Lysis Rack

1. 5 minutes on at Max RPM
2. 5 minutes rest
3. Repeat cycle 8 times for a total of 40 minutes of bead beating

X — TissueLyser II

No tested conditions yielded accurate profiles. This device is not validated by Zymo Research for microbiome research.

X — TissueLyser LT

No tested conditions yielded accurate profiles. This device is not validated by Zymo Research for microbiome research.

X — Retsch Mixer Mill MM 400

No tested conditions yielded accurate profiles. This device is not validated by Zymo Research for microbiome research.

Ordering Information

Product Description	Catalog No.	Size
***Quick*-DNA™ Fecal/Soil Microbe Microprep Kit**	D6012	50 Preps.
***Quick*-DNA™ Fecal/Soil Microbe Miniprep Kit**	D6010	50 Preps.
***Quick*-DNA™ Fecal/Soil Microbe Midiprep Kit**	D6110	25 Preps.
***Quick*-DNA™ Fecal/Soil 96 Kit**	D6011	2 x 96 Preps.

Individual Kit Components	Catalog No.	Amount
**ZR BashingBead™ Lysis Tubes (0.1 & 0.5 mm)**	S6012-50	50 Tubes
**BashingBead™ Buffer**	D6001-3-40	40 ml
**Genomic Lysis Buffer**	D3004-1-100	100 ml
**DNA Pre-Wash Buffer**	D3004-5-15	15 ml
**g-DNA Wash Buffer**	D3004-2-50	50 ml
**DNA Elution Buffer**	D3004-4-10	10 ml
**Prep Solution**	D6035-1-30	30 ml
**Zymo-Spin™ III-F Filters**	C1057-50	50 Pack
**Zymo-Spin™ IICR Columns**	C1078-50	50 Pack
***OneStep*™ PCR Inhibitor Removal Kit**	D6030	50 Preps.
**Collection Tubes**	C1001-50<br>C1001-500<br>C1001-1000	50 Pack<br>500 Pack<br>1,000 Pack

Lysis Instruments	Catalog No.	Amount
**Horizontal Microtube Holder**	S5001-7	1 Unit
**Vortex-Genie® 2, 120V**	S5001	1 Unit
**Vortex-Genie® 2, 230V**	S5002	1 Unit
**Digital Vortex-Genie® 2, 120 V**	S5003	1 Unit
**Digital Vortex-Genie® 2, 230 V**	S5004	1 Unit

The Vortex-Genie® 2 paired with the Horizontal Microtube Holder has been validated for efficient microbial lysis with the ZR BashingBead Lysis Tubes (0.1 & 0.5 mm). See the Appendix for more details.

100% satisfaction guarantee on all Zymo Research products, or your money back.

Zymo Research is committed to simplifying your research with quality products and services. If you are dissatisfied with this product for any reason, please call 1(888) 882-9682.

Integrity of kit components is guaranteed for up to one year from date of purchase.
Reagents are routinely tested on a lot-to-lot basis to ensure they provide the highest performance and reliability.

This product is for research use only and should only be used by trained professionals. It is not for use in diagnostic procedures. Some reagents included with this kit are irritants. Wear protective gloves and eye protection. Follow the safety guidelines and rules enacted by your research institution or facility.

™ Trademarks of Zymo Research Corporation
Other trademarks: Vortex Genie® (Scientific Industries, Inc.), Precellys® (Bertin Instruments), Fastprep-24™ (MP Biomedicals), Bead Ruptor® (Omni International).

ZYMO RESEARCH

The **BEAUTY** *of* **SCIENCE** *is to Make Things* **SIMPLE**®

tech@zymoresearch.com www.zymoresearch.com Toll Free: (888) 882-9682

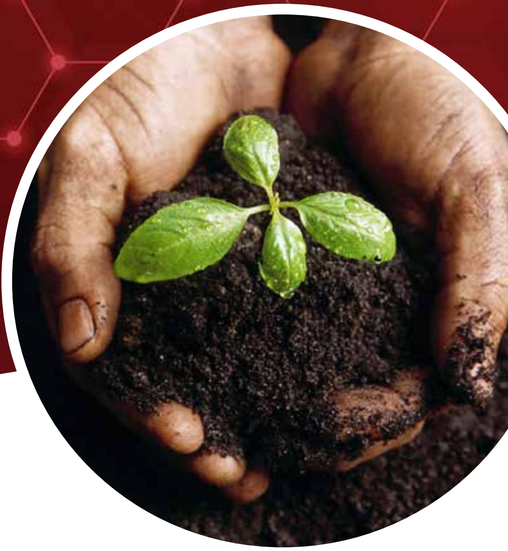

FastDNA™ SPIN Kit for Soil

Rapid isolation of PCR-ready genomic DNA from soil and other environmental samples

Cat. No. **116560200/116560300**

Size: **50 preps/100 preps**

Storage: **Ambient Temperature – 15-30 °C**

Revision Date: **2021-05**

Choose the Best Homogenization and Extraction for Your Application

INSTRUMENTS

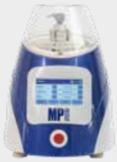

FastPrep-24™ 5G

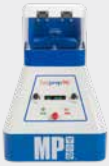

FastPrep-96™

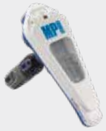

Super FastPrep-2™

ADAPTERS

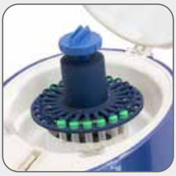

Metal

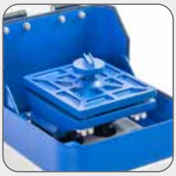

High Throughput

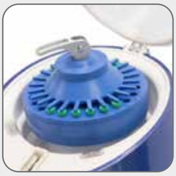

Cryogenic

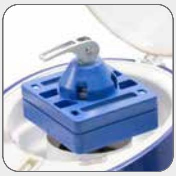

Large Sample Volume

LYSING MATRIX TUBES

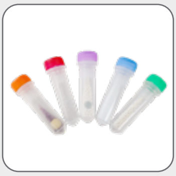

2 mL

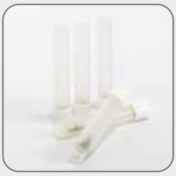

4.5 mL

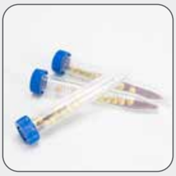

15 mL

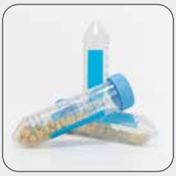

50 mL

EXTRACTION KITS

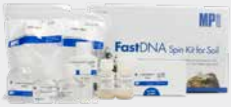

DNA

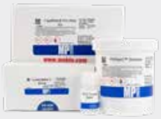

RNA

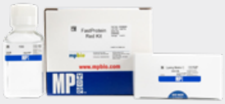

Protein

TABLE OF CONTENTS

1. Introduction to FastDNA™ SPIN Kit for Soil and FastPrep® Instruments 4

2. Kit Components and User Supplied Materials 5

2.1 FastDNA SPIN Kit for Soil Components 5

2.2 User Supplied Materials 5

3. Important Instructions Before Use 6

Sample Lysis with the FastPrep Instrument 6

4. Safety Precautions 6

5. FastDNA SPIN Kit for Soil Typical Workflow 7

6. Troubleshooting Guide 8

7. References 9

8. Related Products 10

9. Product Use Limitation and Warranty 11

1. INTRODUCTION TO FASTDNA™ SPIN KIT FOR SOIL AND FASTPREP® INSTRUMENTS

The FastDNA™ SPIN Kit for Soil quickly and efficiently isolates PCR-ready genomic DNA directly from soil samples in less than 30 minutes. Designed for use with the FastPrep® instruments from MP Biomedicals, plant and animal tissues, bacteria, algae, fungal spores and other members of a soil population are easily lysed within 40 seconds. These benchtop devices use a unique, optimized motion to homogenize samples by multidirectional, simultaneous impaction with lysing matrix particles. FastPrep instruments provide a quick, efficient and highly reproducible homogenization that surpasses traditional extraction methods using enzymatic digestion, sonication, blending, douncing and vortexing.

Samples are placed into 2.0 mL tubes containing Lysing Matrix E, a mixture of ceramic and silica particles designed to efficiently lyse all soil organisms, including historically difficult sources, such as eubacterial spores and endospores, gram positive bacteria, yeast, algae, nematodes and fungi. Homogenization in a FastPrep instrument with Lysing Matrix E takes place in the presence of MT Buffer and Sodium Phosphate Buffer, reagents carefully developed to protect and solubilize nucleic acids and proteins upon cell lysis. These reagents work synergistically to allow extraction of genomic DNA with minimal RNA contamination.

Following lysis, samples are centrifuged to pellet soil, cell debris and lysing matrix. DNA is purified from the supernatant with the Binding Matrix FastDNA procedure using SPIN filters. Eluted DNA is ready for PCR, restriction digest, electrophoresis and any other desired application.

2. KIT COMPONENTS AND USER SUPPLIED MATERIALS

2.1 FastDNA SPIN Kit for Soil Components

	50 preps (116560200)		100 preps (116560300)	
**Product**	**Size**	**Cat. No.**	**Size**	**Cat. No.**
Lysing Matrix E	50 x 2 mL tubes	116914050	100 x 2 mL tubes	116914100
Sodium Phosphate Buffer	60 mL	116560205	120 mL	116560105
MT Buffer	8 mL	116511202	15 mL	116560602
PPS Solution	15 mL	116560203	30 mL	116560403
Binding Matrix	2 x 30 mL	116540408	4 x 30 mL	116540408
SPIN Modules	50 each	116560210	100 each	112080802
Catch Tubes	50 each	116560211	100 each	112080801
Concentrated SEWS-M	12 mL	116540405	12 mL	116540405
DES	20 mL	116540406	20 mL	116540406
BBS Gel Loading Dye	200 µL	116540407	200 µL	116540407
User Manual	1 each	-	1 each	-
Detailed Protocol	1 each	-	1 each	-
Certificate of Analysis	1 each	-	1 each	-

2.2 User Supplied Materials

- FastPrep instrument *(see Section 8)*
- Microcentrifuge to spin 2.0 mL tubes
- Microcentrifuge tubes (2.0 mL and 1.5 mL)
- Clean 15 mL tubes for DNA binding
- Rotator or low-speed vortex

3. IMPORTANT INSTRUCTIONS BEFORE USE

Sample Lysis with the FastPrep Instrument

The fill volume of the lysing matrix tube after addition of Sodium Phosphate and MT Buffers to the sample should allow sufficient air space in the sample tube for efficient FastPrep instrument processing. MP Bio recommends using up to 500 mg of most soil types. Very wet soils or detritus-rich soils may require less sample by mass. Ensure that there is 250–500 µL of empty space in the tube. Sample loss or tube failure may result from overfilling the matrix tube. The matrix tube caps must be secure, but not over-tightened, to prevent sample leakage. If the sample is too large for processing in a single tube, divide the sample and process using multiple tubes.

MP Bio's Lysing Matrix particles and tubes have been rigorously tested and validated in the FastPrep instrument. The use of other products with the FastPrep instrument is not recommended and may result in sample loss or instrument failure. A single 40 second run at a speed setting of 6.0 in the FastPrep instrument is sufficient to lyse almost all samples. If the user experimentally determines that additional processing time is required, the sample should be incubated on ice in the Lysing Matrix E tube for at least 2 minutes between successive FastPrep instrument homogenizations to prevent overheating the sample and tube.

4. SAFETY PRECAUTIONS

Binding Matrix contains components that, when in contact with human tissue, may cause irritation. Wear personal protective equipment to prevent contact with the skin or mucous membranes (gloves, lab coat, and eye protection).

NOTE ▸ *Consult the Material Safety Data Sheet available online at www.mpbio.com.*

5. FASTDNA SPIN KIT FOR SOIL TYPICAL WORKFLOW

Step	Instructions
**1 PREPARE** the sample	**ADD** up to **500 mg** of soil sample, **978 µL** Sodium Phosphate Buffer and **122 µL** MT Buffer to Lysing Matrix E tube
**2 HOMOGENIZE** with the FastPrep *(or similar instrument)*	**LOAD** tube in FastPrep instrument.<br>**PROCESS: 40 s** at a speed setting of **6.0 m/s.**<br>**CENTRIFUGE** at **14,000 x g** for **5-10 mins** to pellet debris
**3 PRECIPITATE** proteins	**TRANSFER** supernatant to a clean 2 mL microcentrifuge tube.<br>**ADD 250 µL** PPS and mix 10 times.<br>**CENTRIFUGE** at **14,000 x g** for **5 mins** to pellet precipitate.
**4 ADJUST** binding conditions	**TRANSFER** supernatant to 15 mL tube.<br>**ADD 1 mL** Binding Matrix Solution. Invert **2 mins** and place tube on a rack for **3 mins.**<br>**DISCARD 500 µL** of supernatant.
**5 BIND** the DNA	**TRANSFER max 600 µL** of DNA Solution to a SPIN Filter Tube.<br>**CENTRIFUGE** at **14,000 x g** for **1 min.** Empty catch tube.<br>Repeat step 5 if the volume of the mixture is higher than 600 µL.
**6 WASH** the SPIN Filter	**ADD 500 µL** prepared SEWS-M Solution.<br>**CENTRIFUGE** at **14,000 x g** for **1 min.**<br>Empty catch tube.
**7 DRY** the SPIN Filter	**CENTRIFUGE** again at **14,000 x g** for **2 mins.**<br>**AIR DRY** SPIN Filter for **5 mins** at room temperature.
**8 ELUTE** the DNA	**ADD 50–100 µL** DES Elution Solution.<br>**CENTRIFUGE** at **14,000 x g** for **1 min.**<br>DNA in the catch tube is ready-to-use.

6. TROUBLESHOOTING GUIDE

Observation	Cause	Solution
**Wet Soil Sample**		Centrifuge sample for 30 seconds at 10,000 x g. Decant most of the liquid, place in the lysing matrix tube and continue with protocol.
**Low DNA Yield in Eluate**	Insufficient Lysis	Add processing cycles with 2 minutes incubation on ice between cycles to avoid excessive heat buildup.
	Insufficient Binding Matrix	Thoroughly mix Binding Matrix before dispensing for each sample.
	Ethanol Not Added to concentrated SEWS-M	Make sure to add 100 mL of 100% Ethanol to concentrated SEWS-M before use.
	DNA Not Eluted Efficiently	After resuspending binding matrix with DES solution, incubate for 5 minutes at 55 °C before centrifuging the final eluate.
**DNA Does Not Amplify**	Excess DNA	Dilute DNA accordingly.
	Non-Specific Bands	Further purification of DNA may be necessary, for example, using one of MP Bio's GENECLEAN® Kits.
	Verify PCR Optimization	Changing reaction conditions or primer selection may be necessary.
**DNA Fragmented**	Use Care with Liquid Transfer	Gently and thoroughly mix the Binding Matrix bound DNA with SEWS-M solution or DES. Use wide-bore pipet tips.
	Optimize Lysis Conditions	Lower the speed and/or duration settings of the FastPrep.
**Low A_{260}/A_{280} Ratios for Purified DNA**	Ethanol Not Added to concentrated SEWS-M	Make sure to add 100 mL of 100% Ethanol to concentrated SEWS-M before use.
	Proteins Not Removed Efficiently	Efficiently mix PPS solution and lysate (step 6 in detailed protocol) by inverting tube at least 10 times or mix by pipetting. Incubate on ice for 5 minutes to further precipitate proteins.
	Contaminants Not Removed Efficiently	Gently and thoroughly mix the Binding Matrix bound DNA with SEWS-M solution or DES. Use wide-bore pipet tips.
**Elevated A_{230} Absorbance**	Proteins Not Removed Efficiently	Efficiently mix PPS solution and lysate (step 6) by inverting tube at least 10 times or mix by pipetting. Incubate on ice for 5 minutes to further precipitate proteins.
	Contaminants Not Removed Efficiently	Repeat PPS treatment (step 6). Gently and thoroughly mix the Binding Matrix bound DNA with SEWS-M solution or DES. Use wide-bore pipet tips.
	Residual Ethanol in the Final Eluate	Centrifuge for an additional 2 minutes (step 14) to remove excess SEWS-M solution from the column. Incubate sample at 60 °C during step 15 to aid in removing residual ethanol.

7. REFERENCES

Kaushal, R.; Peng, L.; Singh, S.S.; Zhang, M.; Zhang, X.; Vílchez, J.I.; Wang, Z.; He, D.; Yang, Y.; Lv, S.; Xu, Z.; Morcillo, R.J.L.; Wang, W.; Huang, W.; Paré, P.W.; Song, C.-P.; Zhu, J.-K.; Liu, R.; Zhong, W.; Ma, P.; Zhang, H. *Microbiome*. **2021**, *9*, 57. DOI 10.1186/s40168-020-00966-y

Xu, T.; Jiang, W.; Qin, D.; Liu, T.; Zhang, J.; Chen, W.; Gao, L. *Sci Rep*. **2021**, *11*, 5773. DOI 10.1038/s41598-021-85281-8

Rüger, L.; Feng, K.; Dumack, K.; Freudenthal, J.; Chen, Y.; Sun, R.; Wilson, M.; Yu, P.; Sun, B.; Deng, Y.; Hochholdinger, F.; Vetterlein, D.; Bonkowski, M. *Front Microbiol*. **2021**, *12*, 614501. DOI 10.3389/fmicb.2021.614501

Juan Song, J.; Min, L.J.; Wu, J.R.; He, Q.; Chen, F.M.; Wang Y. *PLoS One*. **2021**, *16(2)*, e0247309. DOI 10.1371/journal.pone.0247309

Li, Y.; Li, Q.; Chen, S. *Int J Mol Sci*. **2021**, *22(3)*, 1460. DOI 10.3390/ijms22031460

Díaz-Torres, O.; de Anda, J.; Lugo-Melchor, O.Y.; Pacheco, A.; Orozco-Nunnelly, D.A.; Shear, H.; Senés-Guerrero, C.; Gradilla-Hernández, S.M. *Front Microbiol*. **2021**, *12*, 617151. DOI 10.3389/fmicb.2021.617151

Shiozaki, T.; Itoh, F.; Hirose, Y.;Onodera, J.; Kuwata, A.; Harada, N. *PLoS One*. **2021**, *16(2)*, e0245936. DOI1371/journal.pone.0245936

Yekta, S.S.; Liu, T.; Anacleto, T.M.; Bjerg, M.A.; Šafarič, L.; Goux, X.; Karlsson, A.; Björn, A.; Schnürer, A. *Biotechnol Biofuels*. **2021**, *14*, 56. DOI 10.1186/s13068-021-01913-1

Brown, C.L.; Keenum, I.M.; Dai, D.; Zhang, L.; Vikesland, P.J.; Pruden, A. *Sci Rep*. **2021**, *11*, 3753. DOI 10.1038/s41598-021-83081-8

8. RELATED PRODUCTS

Product Name	Cat. No.
**Instruments**	
FastPrep-24™ Classic	116004500
FastPrep-24™ 5G	116005500
FastPrep-96™	116010500
Super FastPrep-2™	116012500
MPure-12™	117002200
**Kits**	
GENECLEAN® SPIN KIT	111101200
GENECLEAN® Kit	111001200
GENECLEAN® II Kit	111001400
GENECLEAN® III Kit	111001600
FastRNA™ Pro Soil-Direct Kit	116070050
FastRNA™ Pro Soil-Indirect Kit	116075050
FastDNA™ SPIN Kit for Soil, 50 mL tubes	116560600
FastDNA™-96 Soil Microbe DNA Kit	119696200
FastPROTEIN™ Blue Matrix	116550400
FastPROTEIN™ Red Matrix	116550600
**Lysing Matrix Tubes**	
Lysing Matrix A, 2 mL	116910050
Lysing Matrix B, 2 mL	116911050
Lysing Matrix C, 2 mL	116912050
Lysing Matrix D, 2 mL	116913050
Lysing Matrix E, 2 mL	116914050

9. PRODUCT USE LIMITATION AND WARRANTY

Unless otherwise indicated, this product is for research use only. Purchase of MP Biomedicals' products does not grant rights to reproduce, modify, or package the products or any derivative thereof to third parties. MP Biomedicals makes no warranty of any kind, expressed or implied, including merchantability or fitness for any particular purpose, except that the products sold will meet our specifications at the time of delivery. Buyer's exclusive remedy and the sole liability of MP Biomedicals hereunder shall be limited to, at our discretion, no replacement or compensation, product credits, refund of the purchase price of, or the replacement of material that do not meet our specifications.

By acceptance of the product, buyer indemnifies and holds MP Biomedicals harmless against, and assumes all liability for, the consequence of its use or misuse by the buyer, its employees or others, including, but not limited to, the cost of handling. Said refund or replacement is conditioned on buyer notification of MP Biomedicals within thirty (30) days of reciept of product. Failure of buyer to give said notice within thirty (30) days shall constitute a waiver by the buyer of all claims hereunder with respect to said material(s).

FastDNA™, FastRNA™, FastPrep®, GENECLEAN® and BIO 101® Systems are trademarks of MP Biomedicals, LLC.

MP Biomedicals

MP BIOMEDICALS

AMERICAS: 800.854.0530 | custserv.na@mpbio.com
EUROPE: 00800.7777.9999 | custserv.eur@mpbio.com
APAC: +65 6775.0008 | custserv.ap@mpbio.com

www.mpbio.com
